# Determining the Control Circuitry of Redox Metabolism at the Genome-Scale

**DOI:** 10.1371/journal.pgen.1004264

**Published:** 2014-04-03

**Authors:** Stephen Federowicz, Donghyuk Kim, Ali Ebrahim, Joshua Lerman, Harish Nagarajan, Byung-kwan Cho, Karsten Zengler, Bernhard Palsson

**Affiliations:** 1 Department of Bioengineering, University of California San Diego, La Jolla, California, United States of America; 2 Bioinformatics and Systems Biology Program, University of California San Diego, La Jolla, California, United States of America; 3 Novo Nordisk Foundation Center for Biosustainability, Technical University of Denmark, Lyngby, Denmark; Institute of Molecular and Cell Biology (IMCB), A*STAR, Singapore

## Abstract

Determining how facultative anaerobic organisms sense and direct cellular responses to electron acceptor availability has been a subject of intense study. However, even in the model organism *Escherichia coli*, established mechanisms only explain a small fraction of the hundreds of genes that are regulated during electron acceptor shifts. Here we propose a qualitative model that accounts for the full breadth of regulated genes by detailing how two global transcription factors (TFs), ArcA and Fnr of *E. coli*, sense key metabolic redox ratios and act on a genome-wide basis to regulate anabolic, catabolic, and energy generation pathways. We first fill gaps in our knowledge of this transcriptional regulatory network by carrying out ChIP-chip and gene expression experiments to identify 463 regulatory events. We then interfaced this reconstructed regulatory network with a highly curated genome-scale metabolic model to show that ArcA and Fnr regulate >80% of total metabolic flux and 96% of differential gene expression across fermentative and nitrate respiratory conditions. Based on the data, we propose a feedforward with feedback trim regulatory scheme, given the extensive repression of catabolic genes by ArcA and extensive activation of chemiosmotic genes by Fnr. We further corroborated this regulatory scheme by showing a 0.71 r^2^ (p<1e-6) correlation between changes in metabolic flux and changes in regulatory activity across fermentative and nitrate respiratory conditions. Finally, we are able to relate the proposed model to a wealth of previously generated data by contextualizing the existing transcriptional regulatory network.

## Author Summary

All heterotrophic organisms must balance the deployment of consumed carbon compounds between growth and the generation of energy. These two competing objectives have been shown, both computationally and experimentally, to exist as the principal dimensions of the function of metabolic networks. Each of these dimensions can also be thought of as the familiar metabolic functions of catabolism, anabolism, and generation of energy. Here we detail how two global transcription factors (TFs), ArcA and Fnr of *Escherichia coli* that sense redox ratios, act on a genome-wide basis to coordinately regulate these global metabolic functions through transcriptional control of enzyme and transporter levels in changing environments. A model results from the study that shows how global transcription factors regulate global dimensions of metabolism and form a regulatory hierarchy that reflects the structural hierarchy of the metabolic network.

## Introduction

Regulation of metabolism in response to shifting availability of electron acceptors is a fundamental process in all of biology and is a critical subject for the understanding of pathogenesis, cancer metabolism, and industrial biotechnology. However, even in the model organism *Escherichia coli*, the regulatory network for this fundamental metabolic function has not been fully elucidated. It has long been known that facultative anaerobes will hierarchically utilize external electron acceptors relative to the free energy change provided by each [1], [2]. Oxygen exists at the top of the hierarchy, electron acceptors like NO_3_ in the middle, and lactate or acetate or other fermentation products are at the bottom [3]–[5]. Many detailed studies have determined that the transcription factors (TFs) ArcA and Fnr are the key players in managing this hierarchy through the activation or repression of the electron transport chain (ETC) machinery specific to an available electron acceptor [6]–[11]. It is also largely understood how ArcA senses redox via the flow of reducing equivalents through the ETC, and how Fnr directly senses levels of dissolved O_2_ [1], [12], [13] and glutathione [14], [15]. However, it is not clear how these two TFs work together and more importantly why they regulate hundreds of gene products that lie outside of the ETC and energy metabolism [3], [5]?

Even though many biochemical details of redox regulation have been elucidated [6], [8], [16], systems level principles for the global regulatory response throughout the anaerobic shift remain elusive. An important missing piece is a clear framework, or design principle, that elucidates how hundreds of transcriptionally regulated gene products are coordinately regulated to produce the necessary quantitative shifts in metabolic flux states. On the purely metabolic side, certain design principles have emerged through the analysis of stoichiometric models that identified growth and energy generation as the two principal dimensions of metabolic network function [17]–[19]. It was further shown that linear combinations of these two dimensions could account for observed flux patterns throughout nutrient limitations and the anaerobic shift [18], [20]. A question now becomes, what are the corresponding global TFs and how do they coordinately regulate all the gene products which enable the metabolic flux map to shift from one optimal state to another?

Here we show how the global TFs ArcA and Fnr coordinately regulate the primary metabolic dimensions of growth and energy generation. We integrated polyomic data sets and used genome-scale metabolic models to enable a mechanistic understanding of hundreds of simultaneous and individual regulatory events. This analysis subsequently provides a link between global regulatory circuits and global optimality in microbial metabolism.

## Results

### Genome-scale identification of TF regulatory events

**Figure 1:**
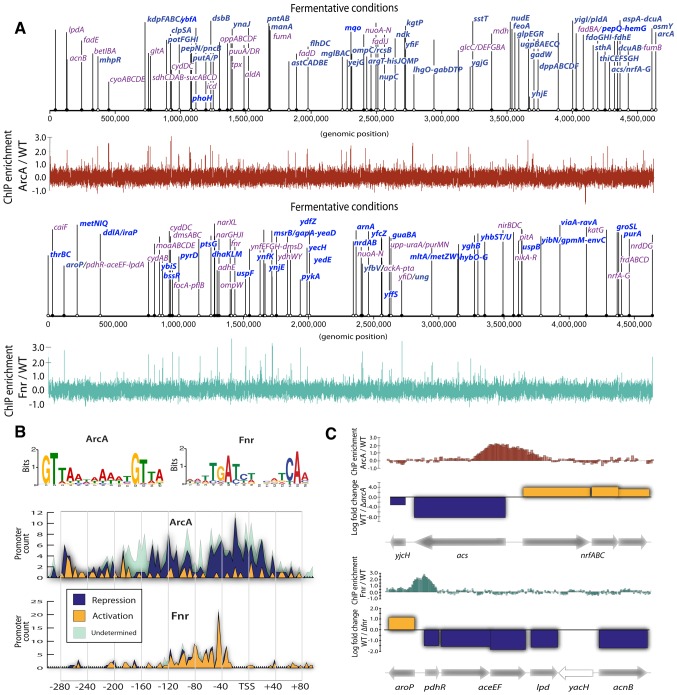
ChIP-chip reveals hundreds of new binding regions and regulatory mechanisms. (A) Triplicate averaged tracks of ChIP-chip intensity plotted along the length of the genome for ArcA and Fnr under fermentation. We show 83% of previously reported regulatory regions are confirmed (purple) and 132 binding regions (bright blue) are newly discovered relative to RegulonDB. All discovered peaks are shown and operon names included when ChIP peaks also corresponded to differential gene expression for a given operon. (B) Binding motifs are recovered from ChIP binding sites. Histograms of the frequency of motif occurrence relative to the transcription start site (TSS) are plotted and overlaid with gene expression data to reveal ArcA repression via blocking of the −35 box and Fnr activation via upstream binding at −41.5. (C) Transcription factor mediated bi-directional transcription is observed in which a single binding region is shown to regulate divergently transcribed transcriptional units.

We first identified individual TF regulatory events at the genome-scale. Side-by-side measurements of RNA transcript abundance and TF binding were carried out to determine the structure and causality in *E. coli*'s transcriptional regulatory network (TRN). ChIP-chip assays for ArcA and Fnr were performed under both fermentative and nitrate respiratory conditions (Figure 1A). Gene expression measurements were then used to determine causality of activation or repression for each ArcA or Fnr binding site under these same two conditions (as detailed in the later heatmap figure legend, Figure S1). We found 102, and 86 (and 143 and 132) binding regions and 58 and 54 (and 95 and 55) causal regulatory events for ArcA and Fnr under fermentation (and nitrate respiration) conditions, respectively (Figure 1A, Tables S1, S2, S3, S4). We then compiled the set of genomic sequences underlying these binding regions for each of the TFs and used the MEME program [21] to recover previously identified binding motifs [22], [23] (Figure 1B, Tables S5, S6). We confirmed 180 of 216 (83%) previously known regulatory events [24] and discovered 132 new binding regions relative to RegulonDB (Figure 1A), representing an increase of 74% over current knowledge of the regulatory functions of these two TFs. We further performed a detailed comparison of our results to recently published works [16], [25] to determine a 78% overlap in ArcA binding sites and a 50% overlap in Fnr binding sites under fermentative conditions (Figures S5, S6, S7). In addition, we report 88 novel binding sites for ArcA and 52 novel binding sites for Fnr under nitrate respiratory conditions highlighting plasticity of the network throughout shifting external electron acceptors.

We then integrated transcription start sites (TSS) [26] with TF binding regions to identify promoter architectures [27]. The location of TF binding motifs within experimentally determined binding regions were used to prepare histograms of the frequency of TF binding relative to the TSS (Figure 1B). This analysis showed that ArcA spans the TSS or −35 box region and represses transcription while Fnr spans the −41.5 or alpha carboxy terminal domain and activates transcription [27]. While each of these regulatory strategies have been shown previously, here can we show that each strategy is ubiquitous at the genome-scale.

### Discovery of transcription factor mediated bidirectional transcription

Novel cases of divergent transcriptional regulation were found in this data. The integration of binding regions with gene expression data revealed 42 regions where two divergent transcriptional units (TUs) were simultaneously regulated by a single binding event. Divergent transcriptional regulation has been observed previously [28] and is known to be mediated by transcription factors in certain cases. However, systematic regulation by global TFs has only been observed in limited cases [29]. We observe a total of 19 inverse, 16 dual activation, and 13 dual repression events for a total of 48 events spread across the 42 regions as some recur under different experimental conditions.

Two examples (Figure 1C) highlight this ‘hard coupling’ of the transcriptional regulation of seemingly unrelated but contextually dependent pathways. The *acs-nrfABCDE* system represents a lowest common denominator coupling between acetyl-coA synthetase (*acs*) acetate scavenging to acetyl-coA and usage of acetyl-coA via the TCA cycle and *nrfABCDE* nitrite reductase. Similarly the *aroP-pdhR* system couples the transport of aromatic amino acids to the regulation of pyruvate that acts as their principal precursor molecule.

The link between the *acs* and *nrfABCD* systems has been inferred/suggested in previous work which attempted to understand how *E. coli* could survive on acetate as a sole carbon source under anaerobic conditions [30]. In particular, *E. coli* cannot utilize acetate under fully anaerobic conditions because acetate must be scavenged into acetyl-coA via *acs* and then utilized by the TCA cycle. Anaerobically the TCA cycle cannot be used unless there is an electron acceptor in the ETC to enable oxidative phosphorylation. Thus, some usage of the TCA cycle via an alternative electron acceptor such as nitrite or nitrate is necessary for *E. coli* to utilize acetate and acetyl-coA anaerobically. This metabolic feature is physiologically crucial in the gut environment that is rich in fatty acids that cannot be used if *E.coli* does not utilize alternative electron acceptors like nitrite. Hence, the direct coupling of *acs* and *nrfABCD* through bidirectional transcriptional regulation is consistent with the necessity of a flux through the *nrfABCD* system in order for the acetyl-coA formed by *acs* to be utilized. The transcriptional coupling acts as bidirectional gate controlled by ArcA and the redox state of the cell to coordinate this evolutionarily crucial metabolic capability.

**Figure 2:**
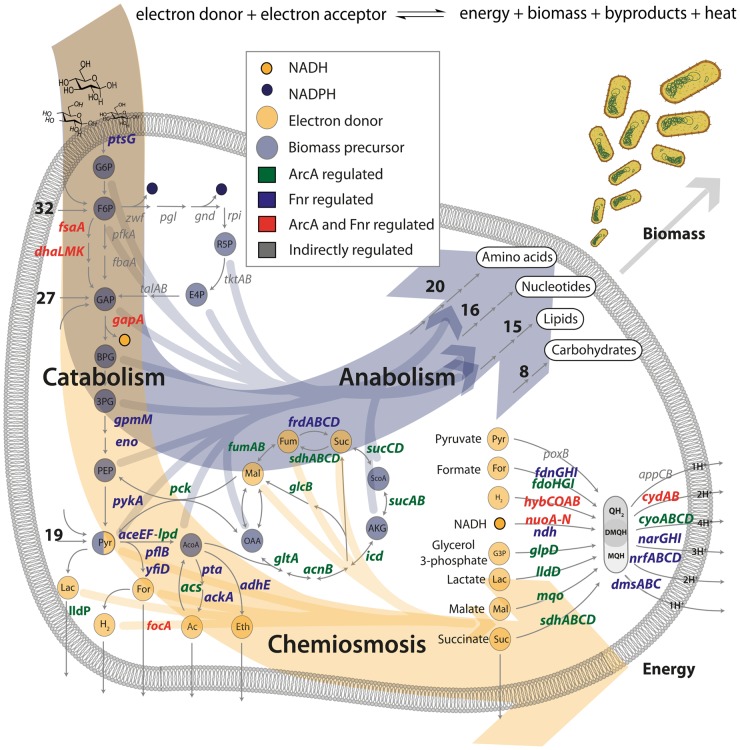
ArcA and Fnr ubiquitously regulate the three branches of metabolism. Transcription factor regulated gene products are shown in terms of their biological context in the metabolic network. The principal dimensions of metabolism are shown as two large arrows for the formation of biomass or energy. All of the 12 biomass precursors (10/12 regulated) and 9 primary electron donors (9/9 regulated) are shown with arrows flowing into biomass formation or chemiosmosis. The anabolic process is pictorialized with the number of genes regulated in each of the biosynthetic pathways and the chemiosmotic process is shown primarily via the electron transport chain. Numbers indicate the number of regulated genes upstream or downstream of key precursors (e.g. 19 genes encoding reactions for transport and secondary catabolism pathways are regulated upstream of pyruvate).

Similarly the *aroP-pdhR* system couples the transport of aromatic amino acids to the regulation of pyruvate that acts as their principal precursor molecule through the action of Fnr. To understand the network level connection between the aromatic amino acid transporter (*aroP*) and the pyruvate dehydrogenase repressor TF (*pdhR*) one can examine Figure 2, which shows the connection between catabolic biomass precursors and biosynthetic pathways. Tyrosine and tryptophan are both made directly from PEP that is rapidly dephosphorylated into pyruvate. The corresponding activation of *aroP* and repression of *pdhR* is consistent with an increased need for amino acid transport when the precursors for biosynthesis (PEP) are critical to maintain cellular energy levels. This characteristic is supported by a dampening of the switch upon the transition to nitrate respiration, resulting in decreased transporter expression when less pyruvate is needed for fermentation and can thus be shuttled to amino acid biosynthesis. In general, *pdhR* acts as a classic repressor that “pops off” of its binding site in the presence of pyruvate and hence allows expression of pyruvate dehydrogenase and other oxidative enzymes. Anaerobically pyruvate dehydrogenase (*aceEF-lpd*) is repressed regardless of *pdhR* by ArcA and Fnr and given that there is also a higher concentration of pyruvate it would presumably not be active. Thus, while this switch is highlighted anaerobically in that full repression of *pdhR* is concomitant with *aroP* activation its physiological significance is more prevalent under nitrate or even fully aerobic conditions in which it can function to directly couple and balance the catabolic and anabolic demands around pyruvate which acts as a critical second messenger in the aerobic-anaerobic shift [6]. It is very insightful to view such a switch as it is ramped fully up under anaerobic conditions and then turned down under nitrate respiration to maintain a physiologically crucial metabolic balance.

### Ubiquitous regulation of the principal dimensions of metabolism by ArcA and Fnr

**Figure 3:**
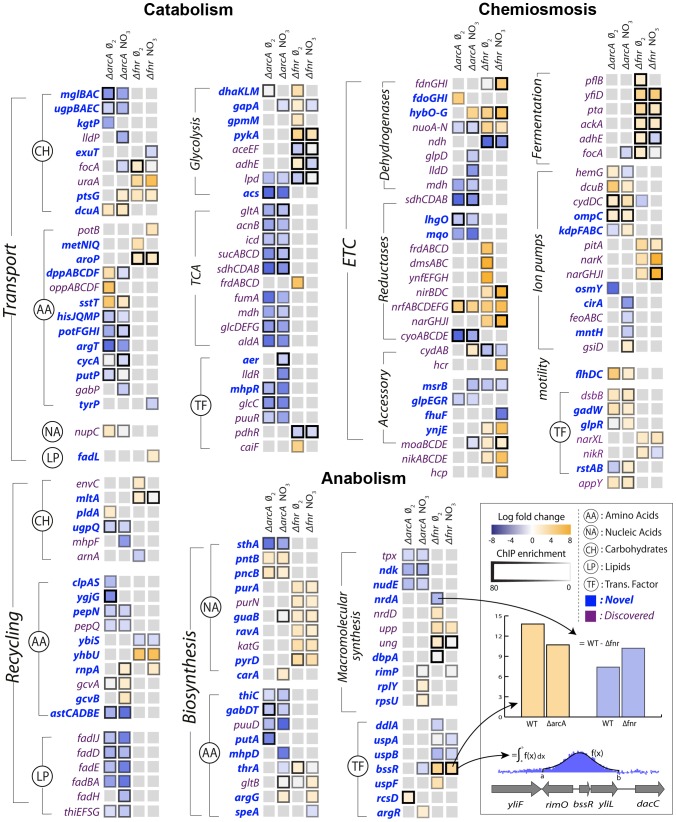
Integration of ChIP-chip, gene expression, and biological context. Specific regulation of each gene product by ArcA or Fnr under strictly anaerobic and nitrate respiratory conditions are shown as columns. Each box is the result of integration between ChIP-chip and gene expression data in which a TF binding peak was identified and gene expression microarrays showed differential expression upon knockout of the transcription factor in matched conditions. The genes are grouped biologically according to the principal dimensions described in figure 2. Immediate broad trends that emerge are catabolic repression by ArcA and chemiosmotic and anabolic activation by Fnr.

Previous work has identified biomass production and energy production as the two principal dimensions characterizing the overall function of metabolic networks [17]–[19]. This duality in function is conceptually equivalent to considering heterotrophic metabolism as the standard combustion equation (Figure 2) in which an electron donor (glucose) is broken apart with an electron acceptor (oxygen, nitrate, etc.) to form biomass, energy, waste and heat. Here we use the terms catabolism to describe oxidation of the electron donor, anabolism to describe biomass formation, and chemiosmosis to describe energy generation. The genes in each of these categories were determined by a manual curation of the *E. coli* metabolic model [31] and associated literature sources [4], [26]. Catabolic genes correspond to nutrient transporters, recycling machinery, and central catabolic machinery. Anabolic genes correspond to biosynthetic and macromolecular synthesis pathways. Chemiosmotic genes correspond to the electron transport chain (ETC), fermentation pathways, and ion pumps (Figure 3).

From the data sets described above, the regulation of these three classes of genes by ArcA and Fnr can be analyzed using their metabolic functions as context. ArcA and Fnr directly regulate a total of 127 catabolic genes including 49 transporter genes, 38 recycling or secondary catabolic enzymes, 33 central metabolic genes, and 7 associated TFs (Figures 2,3). In particular, recovery of all of the classic targets of ArcA and Fnr is complemented by the simultaneous discovery of transporter genes and recycling enzymes like peptidases and proteases (Figure 3). It can also be recognized that there existed many classically unknown glycolytic targets along with generally unrecognized activation of the glucose transporter *ptsG*. Activation of *ptsG* by Fnr is consistent with the fact that cells nearly double their uptake of carbon during fermentative growth compared with aerobic growth.

In anabolism, ArcA and Fnr directly regulate 54 genes including 34 metabolite synthesis genes, 14 macromolecular synthesis genes, and 6 TFs. Broad trends of nucleotide biosynthesis activation and amino acid biosynthetic activation of nucleotide precursors is consistent with redox related demands. However, perhaps the most important of these findings is the regulation of both transhydrogenases (*sthA*, *pntAB*) in *E. coli*. Previous work has shown that a large portion of the NADPH used for biosynthetic reactions comes from the membrane bound transhydrogenase PntAB [32] and that the soluble SthA is used for re-oxidation of NADPH under aerobic growth with excess glucose. Our data shows that ArcA activates *pntAB* and represses *sthA* in a redox-dependent fashion consistent with an increased need for NADPH under nitrate respiration relative to fermentation (Figure 3). This regulatory shuttling of reduction equivalents thus plays a critical role in maintaining the balance between growth and energy generation by increasing growth only once when energy demands are satisfied.

In the chemiosmotic category we observe regulation of 120 genes including 83 genes of the ETC, 6 for fermentation, 21 for ion pumps, 2 for motility, and 8 TFs. Nearly all of the regulation can be shown to coincide with redox related demands including regulation of ion pumps which coincides with an increased need to maintain a positive electrical gradient across the inner membrane to make up for the diminished proton gradient. We also observed strong regulation of the *flhDC*, *gadW*, and *appY* transcription factors. The *flhDC* system is a master regulator for the motility and flagellum apparatus of the cell that feeds off the chemiosmotic gradient in search of nutrients. *appY* and *gadW* are key regulators of cytochromes and acidic tolerance, respectively. After including regulation through *appY* we can conclude that ArcA and Fnr exhibit control either directly or indirectly over 15 out of the 16 known dehydrogenase and oxidoreductase reactions in *E. coli* [4] (Figures 2,3).

### High-level architecture of the metabolic-regulatory network

**Figure 4:**
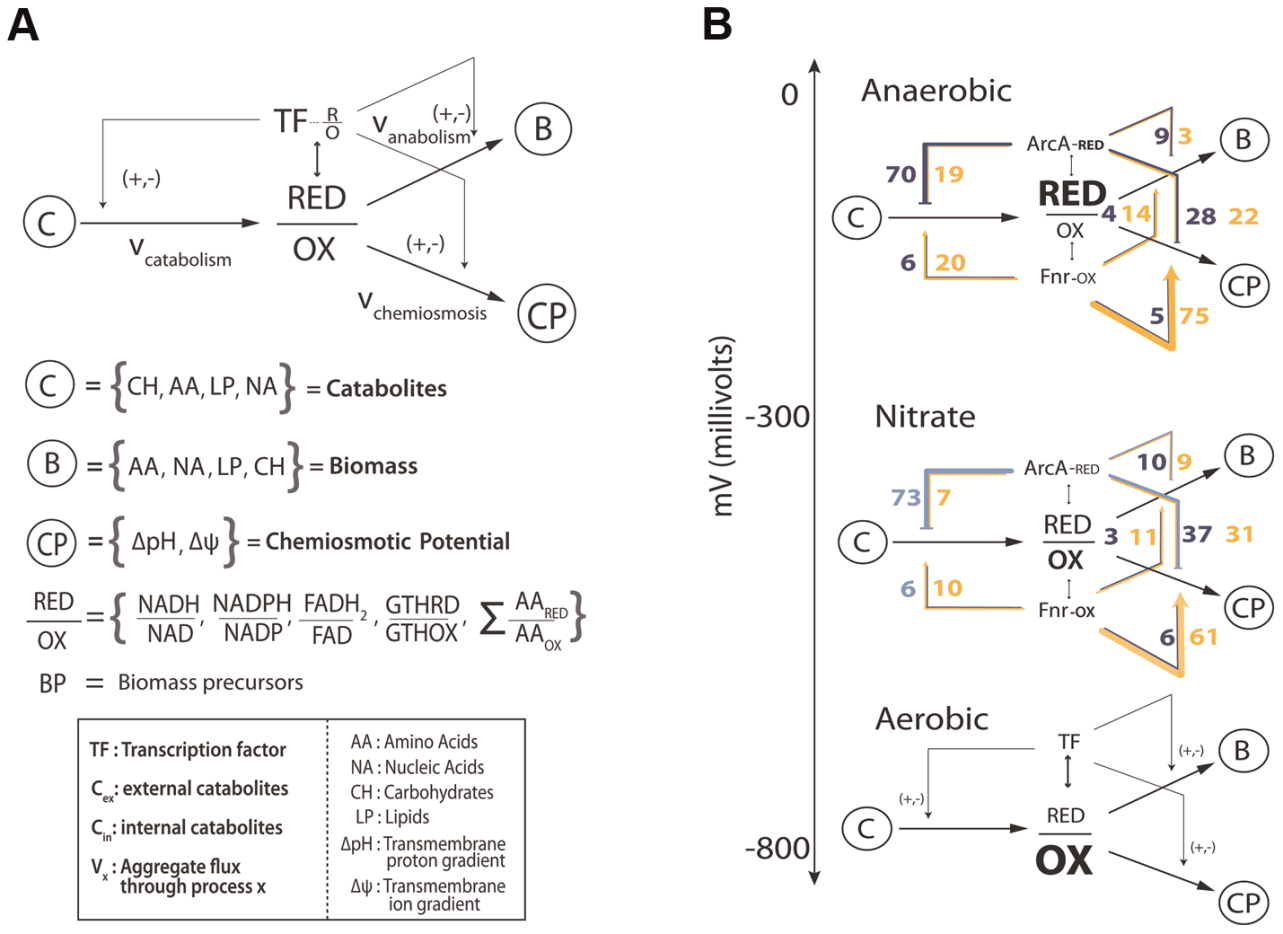
Flow based model of the metabolic-regulatory network explains regulation throughout the anaerobic shift. (A) Considering a mass balance around the ratio of reduced to oxidized molecules allows the unification of catabolism, anabolism, and chemiosmosis into a single process. The ratio of reduced to oxidized molecules is then sensed by ArcA and Fnr to elicit corresponding feedforward and feedback regulatory circuitry which allows the cell to maintain this critical ratio. (B) Mapping of the regulation of gene products (Figure 3) for each branch of the circuit reveals a broad trend of feedforward with feedback-trim regulation. Under fermentative conditions the redox ratio is high and the observed regulation works to lower the input and activate the output to bring the ratio down. Under nitrate respiration, the ratio drops and the circuit maintains a similar number of connections but is shown to decrease in gross activity levels.

Enumerating regulatory events is informative, but how do they all together form a coherent regulatory logic that produces meaningful physiological states? Network analysis of these regulatory interactions reveals a qualitative feedforward and feedback flow-based model of the primary metabolic dimensions (Figure 4A). The model input is the total set of catabolites (glucose or electron donor) available to the cell that are oxidized based on the availability of an electron acceptor into a ratio of reduced to oxidized components. These components (primarily NADH/NAD and NADPH/NADP) are then used by the anabolic machinery to generate biomass, or by the chemiosmotic machinery to generate energy as outputs. The ratio of reduced-to-oxidized components is sensed by ArcA and Fnr [1], and they can feedback and feedforward regulate the catabolic, anabolic, and chemiosmotic processes in a coordinated fashion to maintain the ratio. Consistent with this schema, it has been shown that TFs are ideal flux sensors [33].

### Feedforward with feedback-trim architecture regulates the anaerobic shift

Analyzing the regulatory events within the context of the qualitative flow-based model reveals a feedforward with feedback-trim architecture of the overall regulatory logic. Counting the number of genes that are activated or repressed (Figure 3) provides a measure of the extent of feedforward or feedback regulation exerted (Figure 4B). Under fermentation ArcA represses 70 catabolic genes and Fnr activates 75 chemiosmotic genes. Under nitrate respiration ArcA represses 73 catabolic genes and Fnr activates 61 chemiosmotic output genes. A similar trend is observed for regulation of the anabolic circuitry in which Fnr activates 14 and 11 genes under fermentation and nitrate respiration. This circuitry is consistent with fast sensing of oxygen by Fnr and slow but continuous sensing of redox flow through the ETC by ArcA [34].

The regulatory architecture revealed by this qualitative model is comprehensive and novel, but primarily topological. To more quantitatively assess the functions of the observed transcriptional regulatory architecture on the metabolic network that it regulates we sampled all allowable network flux states of a highly curated genome-scale metabolic model of *E. coli* metabolism [31] under both fermentative and nitrate respiratory conditions. This sampling of allowable flux states of the metabolic network was then integrated with the experimentally determined regulatory architecture to discern the amount of total flux (sum of flux loads across all reactions) regulated by ArcA and Fnr under each of the conditions studied. This calculation revealed that 60% and 57% (and 88% and 80%) of all metabolic flux is directly (and indirectly) controlled by ArcA and Fnr under fermentative and nitrate respiratory conditions respectively (Tables S7, S8). We further show that 69% and 62% of the catabolic fluxes producing each of the redox molecules and biomass precursors along with 71% and 69% of the downstream anabolic and chemiosmotic fluxes are directly regulated under fermentative and nitrate respiratory conditions respectively (Figure S3, Table S9, S10). From a gene level we find that 246 genes are differentially expressed (fdr<.05, fold change >2) between fermentative and nitrate respiratory conditions and that 236/246 or ∼96% of the genes are directly (73) or indirectly (163) regulated by ArcA or Fnr (Table S12). Taken together, these measurements quantify the global metabolic regulation of flux by ArcA and Fnr and provide further evidence towards the proposed feedforward with feedback-trim regulatory architecture.

**Figure 5:**
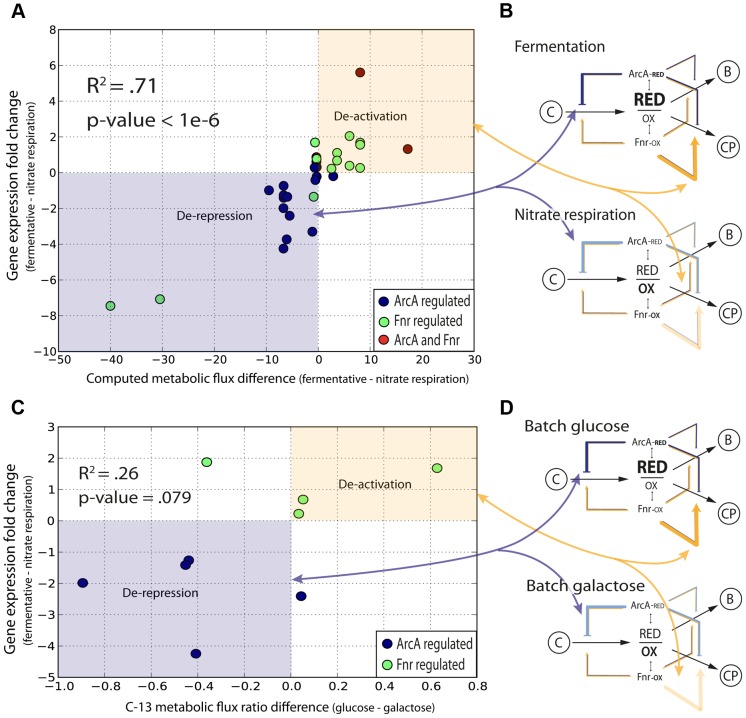
Quantitative correlation of shifts in regulatory strength between experimental conditions with shifts in flux through regulated enzymes. (A) The decrease in activity levels from anaerobic to nitrate is quantified by calculating a correlation between the change in flux for all altered reactions across nitrate and anaerobic conditions with the change in level of regulation across the same conditions. (B) Overlaying information for the specific regulators shows that ArcA is involved in the derepression of key reactions going from fermentation to nitrate respiration and Fnr is involved in deactivation. (C) The shift between glucose and galactose under batch growth mirrors the respiratory shift from fully fermentative to nitrate respiratory conditions. C-13 labeled fluxomic data generated for wild type cells under both glucose and galactose batch conditions is used to generate the same plot as in (A) and is even plotted against the same regulatory strengths between fully fermentative anaerobic cultures and nitrate respiring cultures. (D) One can again see that key ArcA regulated genes are de-repressed whereas Fnr regulated genes are de-activated.

To provide more validation for the feedforward with feedback-trim architecture at the genome-scale we first assessed the set of 91 reactions that significantly differed (flux cutoff of 0.25 mmol/gDW-1 -h-1) between fermentation and nitrate respiration; gDW is denotes grams dry weight. We were then able to show that 89 of the 91 reactions were regulated directly (40 reactions) or indirectly (49 reactions) by ArcA or by Fnr (Table S11). We then calculated the change in flux for each of these 89 reactions between the two conditions along with the change in regulatory strength for the genes encoding these 89 reactions across the same conditions (Table S11). We plotted the change in flux versus the change in regulation (Figure 5A) and calculated an *r^2^* correlation value of 0.71 (*p*<1e-6) for the directly regulated genes. This correlation provides quantitative evidence for the logic of the regulatory circuit in the transition from fermentation to nitrate respiration. The linear positive slope shows not only that the reactions responsible for the redox shift are regulated, but also that these reactions are quantitatively regulated to help minimize the redox ratio in concert with the quantitative model predictions. Most of the ArcA regulated reactions are de-repressed, as indicated by the lightening shade of blue under nitrate respiration (Figure 5B). Most of the Fnr regulated reactions are de-activated as highlighted by the lightening shade of yellow under nitrate respiration (Figure 5B). The broad repression of crucial catabolic genes by ArcA and activation of chemiosmotic genes by Fnr is also shown through analysis of C-13 MFA data generated between wild type and ***Δ*** *fnr* or ***Δ*** *arcA* strains (Figure S8). This trend of redox ratio minimization was so strong that the only outliers resulted in identification of new biology in the form of transport-coupled redox balancing for allosterically regulated amino acid biosynthetic reactions (Figure S4, Text S1).

We then sought to show that this quantitative regulatory model was truly redox dependent and not just fermentative/nitrate respiration specific. We thus took C-13 measured flux data [35] for *E. coli* grown aerobically in batch under either fully respiratory galactose conditions or partially fermentative glucose conditions. Even though both conditions are aerobic, we hypothesized that a similar shift in the redox ratio as observed between fully fermentative and nitrate respiration would occur given the comparison between a partially fermentative and fully respiratory condition. We made the same plot (Figure 5C) as in Figure 5a and even used regulatory strengths taken from the fermentative/nitrate shift. Only 16 flux measurements could be mapped of which only 9 showed any difference between glucose and galactose conditions. Of those 9 fluxes we were able to see a clear correlation for 7 and an overall weak but significant *r^2^* correlation value of .26 (p = .079). This plot again shows genes regulated by ArcA being de-repressed and genes regulated by Fnr being de-activated upon the shift to more oxidative conditions (Figure 5D).

### Hierarchy of the joint metabolic-regulatory network

**Figure 6:**
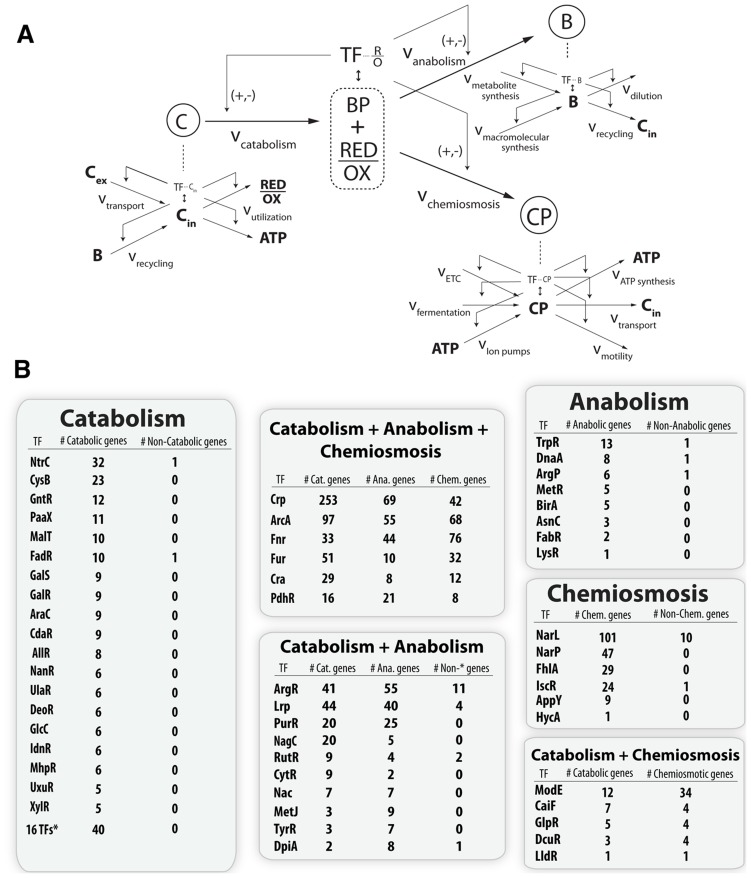
Topological organization of the joint metabolic-regulatory network. (A) Qualitative model where levels of the hierarchy represent a coarse graining of the total metabolic network around pools of key metabolites. Each metabolite has mass balanced production and consumption fluxes and often a corresponding TF sensor that can regulate the input and output fluxes. (B) Quantitative assessment of this regulatory scheme done by curation and classification of the regulatory targets for every TF known to sense a metabolite in the iJO1366 model (Table S13). The classification of a gene product into the catabolic, anabolic, or chemiosmotic metabolic pathways was done identically to the classification of figure 3. The main point is that the hierarchy of the regulatory network does in fact mirror the hierarchical dimensionality of the metabolic network. Regulators which sense a catabolite only regulate catabolic genes, regulators which sense an anabolite only regulate anabolic genes, and regulators which sense a chemiosmotic component only regulate chemiosmotic genes. However, metabolites that exist as both a catabolite and anabolite, or as both a catabolite and chemiosmotic component, tend to have regulators which regulate genes in each of the given categories. Similarly, TFs which sense molecules that are biomass precursors and energy precursors will necessarily globally regulate metabolic genes in all three categories. All curation results are available in a functional form at http://nbviewer.ipython.org/gist/steve-federowicz/f3a1ad0f86158d3f672e.

An expansion of the top-level of the flow-based model contextualizes the function of the hundreds of individual gene products and provides a window into the structure of the full metabolic-regulatory network (Figure 6A). Each different type of catabolite (Figure 3, Figure 4A, Figure 6A) is maintained via production fluxes (transport or recycling) and consumption fluxes (secondary catabolism or central catabolism). The catabolism specific production set consists of genes for amino acid, carbohydrate, lipid, and nucleic acid transport and recycling. The same expansion can be performed for anabolism and chemiosmosis. For anabolism, the total biomass is a result of the sum of the rate of metabolite biosynthesis plus the rate of macromolecular synthesis [36] minus the rate of dilution and recycling. For chemiosmosis, the total gradient is a sum of protons pumped across the inner membrane via the ETC, proton equivalents pumped across the inner membrane via fermentation, and ions translocated across the inner membrane minus the usage of the gradient for ATP production, nutrient transport, and motility [37].

This expansion also accounts for the classically observed hierarchy [38] of the TRN via sensing of lower level metabolites and subsequent regulatory control of the TFs themselves or of the production or consumption pathways for sensed metabolites (Figure 6B). A full tracing of the TRN to explain the effects of the global TF deletion is consistent with 69% of observed differential expression (Figure S2).

## Discussion

This work presents a systems level and genome-scale mechanism for the coordinate action of global transcription factors throughout an electron acceptor shift. Our mechanism accounts for the previously unexplained genes regulated by ArcA and Fnr, it predicts changes in flux patterns, and perhaps most importantly shows that the classically observed hierarchy of transcriptional regulation mirrors the hierarchy of dimensions in the metabolic network. By basing our work off of the extensive body of detailed biological literature and the more recent work of principal dimensionality in metabolic networks we are able to present a systematic and remarkably consistent genome-scale mechanism.

At the local level, we first greatly expanded the number of cases of promoter architectures [39]. This validates and highlights the importance of understanding initiation mechanisms, as they may be extendable to a systems level in future development of computational models. We were then able to make the novel discovery that 42 regions across the genome contained divergently transcribed TUs controlled by a single global TF binding region. We recognize that due to ChIP-chip resolution it is possible (and even likely) that multiple binding sites exist under the larger ChIP peak, however the local proximity still affords the same hard-coupling within the regulon. This hard coupling suggests switch like mechanisms in which sets of seemingly unrelated genes are jointly regulated to obey non-obvious systems level constraints. We identify two such cases of this in the *acs-nrfABCDE* operon and the *aroP-pdhR* operon.

To understand systems level mechanisms of transcriptional regulation we turned to previous work that showed the principal dimensions of a metabolic space were biomass and energy generation. We hypothesized that global regulators must play a role in regulating globally decisive metabolic dimensionality. This hypothesis is supported by broad regulation across all of these main categories and the abilities of ArcA and Fnr to sense the molecules that govern the branch point between the two dimensions.

Although we were able to make an unbiased characterization of the genes in each of the categories using the *iJO*1366 model we were still unsatisfied with such a coarse grained approach and sought to understand the composition of each of the categories. This led us to a hierarchical expansion and classification of pathways around key metabolic intermediates. Going on in this fashion led us to realize that the global transcriptional regulatory hierarchy plays out not only on the level of TF-TF regulation, but perhaps more importantly at the level of global TFs regulating the production or consumption fluxes of lower level metabolites which are correspondingly sensed by other intermediate regulators. In essence, the regulatory network is shaped by the underlying metabolite pools and vice versa.

After determining the broad circuitry of the metabolic-regulatory network we mapped our data onto it and discovered that a strong feedforward with feedback trim architecture dominates at the genome scale. This occurs via ArcA's strong repression of input catabolic circuits coupled with Fnr's strong activation of downstream chemiosmotic and anabolic circuitry. This circuit is corroborated by Fnr's ability to sense oxygen [13] which will diffuse quickly whereas ArcA will more continuously sense the flow of reducing equivalents through the ETC by sensing of the ratio of reduced to oxidized quinones [12]. This pattern of a fast component feeding forward for downstream “planning” coupled with a slower but continuous feedback sensor is a common pattern in basic process control schemes [40]. If coupled with other common process control patterns such as hierarchical and PID control one can envision a process control based model for the entire joint metabolic-regulatory network.

This work presents a formal integration and reconstruction of over 50 years of research on *E. coli* metabolism and its transcriptional regulation. The result is a detailed and coherent hierarchical view of the regulation of the principal dimensions of metabolism through a critical environmental shift. We find that the mathematical notions of optimality in metabolic functions are in line with our observations of global regulation. TRNs are not just TF-gene networks but rather TF-gene-enzyme-reaction flux networks, that are tightly integrated as levels or ratios of metabolites can drive TF activity [41], [42]. The full elucidation of an electron acceptor response in the important model organism, *E. coli*, may have implications for similar metabolic responses in other organisms. For cancer, recent focus has shifted towards an understanding of the metabolic drivers and Warburg effect, where the hypoxia inducible factor (HIF) [43] senses the redox ratio and feedforward or feedback regulates genes producing or consuming reduction potential.

Taken together, we are able to show how the two principal dimensions of metabolism are controlled in a shifting environment by global TFs through the use of polyomic data sets and genome-scale metabolic models. This study is likely to be useful as a guide for similar studies in other organisms where the same tools for experimentation and analysis are available.

## Methods

### Bacterial strains and growth conditions

All strains used in this study were *E. coli* K-12 MG1655 and its derivatives. The *E. coli* strains harboring Fnr-8myc and ArcA-8myc were generated as described previously [44]. The deletion mutants (***Δ*** *fnr and* ***Δ*** *arcA*) were constructed by a **λ** red and FLP-mediated site-specific recombination method. Glycerol stocks of E. coli strains were inoculated into M9 minimal medium containing 0.2% (w/v) carbon source (glucose) and 0.1% (w/v) nitrogen source (NH4Cl), and cultured overnight at 37°C with constant agitation. The cultures were diluted 1∶100 into fresh minimal medium and then cultured at 37°C to an appropriate cell density with constant agitation. For the anaerobic cultures, the minimal medium were flushed with nitrogen and then continuously monitored using a polarographic-dissolved oxygen probe (Cole-Parmer Instruments) to ensure anaerobicity. For nitrate respiration 20 mmol potassium nitrate was added.

### ChIP-chip

To identify Fnr and ArcA binding regions in vivo, we used the ChIP-chip approach as described previously [44], [45]. Briefly, cells at appropriate cells density were cross-linked by 1% formaldehyde at ∼20°C for 25 min. Following the quenching of the unused formaldehyde with a final concentration of 125 mM glycine at ∼20°C for 5 min, the cross-linked cells were harvested and washed three times with 50 ml of ice-cold Trisbuffered saline. The washed cells were resuspended in 0.5 ml lysis buffer composed of 50 mM Tris-HCl (pH 7.5), 100 mM NaCl, 1 mM EDTA, 1 µg/ml RNaseA, protease inhibitor cocktail (Sigma) and 1 kU Ready-Lyse lysozyme Epicentre). The cells were incubated at 37°C for 30 min and then treated with 0.5 ml of 2 Å∼IP buffer with the protease inhibitor cocktail. The lysate was then sonicated four times for 20 s each in an ice bath to fragment the chromatin complexes using a Misonix sonicator 3000 (output level, 2.5). The range of the DNA size resulting from the sonication procedure was 300–1,000 base pairs (bp). The specific antibodies that specifically recognizes myc tag (9E10, Santa Cruz Biotech) were used to immunoprecipitate each chromatin complex, respectively. For the control (mock-IP), 2 µg of normal mouse IgG (Upstate) was added into the cell extract. The remaining ChIP-chip procedures were performed as described previously [44], [45]. The high-density oligonucleotide tiling arrays used to perform ChIP-chip analysis consisted of 371,034 oligonucleotide probes spaced 25 bp apart (25 bp overlap between two probes) across the E. coli genome (Roche NimbleGen). After hybridization and washing steps, the arrays were scanned on an Axon GenePix 4000B scanner and features were extracted as a pair format by using NimbleScan 2.4 software (RocheNimbleGen).

### qPCR

To monitor the enrichment of promoter regions, 1 µL immunoprecipitated DNA was used to carry out gene-specific qPCR. The quantitative real-time PCR of each sample was performed in triplicate using iCycler (Bio-Rad Laboratories) and SYBR green mix (Qiagen). The real-time qPCR conditions were as follows: 25 µL SYBR mix (Qiagen), 1 µL of each primer (10 pM), 1 µL of immunoprecipitated or mock-immunoprecipitated 3DNA and 22 µL of ddH2O. All real-time qPCR reactions were done in triplicates. The samples were cycled to 94°C for 15 s, 52°C for 30 s and 72°C for 30 s (total 40 cycles) on a LightCycler (Bio-Rad). The threshold cycle values were calculated automatically by the iCycler iQ optical system software (Bio-Rad Laboratories). Any primer sequences used were described previously [44].

### Transcriptome analysis

Samples for transcriptome analysis were taken from exponentially growing cells. From the cells treated by RNAprotect Bacteria Reagent (Qiagen), total RNA samples were isolated using RNeasy columns (Qiagen) in accordance with manufacturer's instruction. Total RNA yields were measured using a spectrophotometer (A260), and quality was checked by visualization on agarose gels and by measuring the sample A260/A280 ratio (>1.8). Affymetrix GeneChip E. coli Genome 2.0 arrays were used for genome-scale transcriptional analyses. cDNA synthesis, fragmentation, end-terminus biotin labeling, and array hybridization were performed as recommended by Affymetrix standard protocol. Raw CEL files were analyzed using robust multi-array average for normalization and calculation of probe intensities. The processed probe signals derived from each microarray were averaged for both the wild type and deletion mutant strains.

#### ChIP-chip and expression data analysis

To identify TF-binding regions, we used the peak finding algorithm built into the NimbleScan software. Processing of ChIP-chip data was performed in three steps: normalization, IP/mock-IP ratio computation (log base 2), and enriched region identification. The log2 ratios of each spot in the microarray were calculated from the raw signals obtained from both Cy5 and Cy3 channels, and then the values were scaled by Tukey bi-weight mean. The log2 ratio of Cy5 (IP DNA) to Cy3 (mock-IP DNA) for each point was calculated from the scanned signals. Then, the bi-weight mean of this log2 ratio was subtracted from each point. Each log ratio dataset from duplicate samples was used to identify TF-binding regions using the software (width of sliding window = 300 bp). Our approach to identify the TF-binding regions was to first determine binding locations from each data set and then combine the binding locations from at least five of six datasets to define a binding region using the MetaScope software (http://sbrg.ucsd.edu/Downloads/MetaScope). Raw gene expression CEL files were normalized using background corrected robust multi-array average implemented in the R affy package. To detect differential expression between the wild type and TF deletion strains we applied a two-tailed unpaired students t-test between the experimental triplicates for the wild type and gene deletion strains. This was followed by a false discovery rate adjustment. Before performing the FDR correction we removed all genes that exhibited an expression level below the background across all experiments. The background level was calculated as the average expression level across all intergenic probes. We then only considered genes meeting a 5% FDR (false discovery rate)-adjusted P-value cut-off to be differentially expressed. ChIP binding tracks for Figure 1a and the heatmap for Figure 3 were generated using D3 [46]. Related code is available at http://nbviewer.ipython.org/gist/steve-federowicz/7cceedba73982c0ae995. All raw and processed data have been deposited in NCBI/GEO under accession number GSE55367 (http://www.ncbi.nlm.nih.gov/geo/query/acc.cgi?acc=GSE55367).

### Motif searching

The ArcA and Fnr binding motif analysis was completed using the MEME and FIMO tools from the MEME software suite [21]. We first determined the proper binding motif and then scanned the full genome for its presence. The elicitation of the motif was done using the MEME program on the set of sequences defined by the ArcA and Fnr binding regions respectively. Using default settings the previously determined ArcA and Fnr motifs were recovered and then tailored to the correct size by setting the width parameter to 18-bp and 16-bp respectively. We then used these motifs and the PSPM (position specific probability matrix) generated for each by MEME to rescan the entire genome with the FIMO program.

### Promoter architecture determination

We integrated transcription start sites (TSS) with our TF binding regions to identify promoter architectures genome wide [27], [47]. We first determined the location of motif binding sites within experimentally determined binding regions. We then calculated the distance between motif center position and previously determined TSS locations [26]. Finally, we prepared a histogram of the number of motifs that occur at varying distances relative to the TSS (Figure 1B) and included the gene expression data to determine the regulatory outcome of each binding event. The results showed that ArcA spans the TSS or −35 box region and represses transcription while Fnr spans the −41.5 or alpha carboxy terminal domain [47] and activates transcription. The histograms also reveal the previously reported trend [48] of motif frequency oscillation at a roughly 10.5 bp interval consistent with helical phasing of the DNA strand.

### Genome-scale metabolic sampling

To perform sampling we first generated pFBA [49] constrained models of the iJO1366 [31] metabolic model corresponding to fermentative and nitrate respiratory conditions. Fermentative conditions were simulated by setting the lower bound of the oxygen exchange reaction (EX_o2) to zero. Nitrate respiratory conditions were simulated by setting the lower bound for nitrate uptake (EX_no3) to −20 mmol gDW^−1^ h^−1^ (mirroring experimental addition of 20 mmol KNO_3_) along with the lower bound of EX_o2 set to zero. pFBA constrained models were generated by first using the convertToIrreversible() function of the COBRA toolbox [50] followed by a standard FBA for growth rate. This growth rate was then imposed as a constraint in a subsequent optimization that found the minimum sum of flux able to achieve that growth rate. Finally, using the gpSampler() [50] method we sampled each of the pFBA constrained models. All sampling runs were for a full 24 hours to ensure a mixing fraction below .55. After sampling was performed we took the average across the 7046 sampling points (2n where n = 3,523 reactions in the metabolic model). Sampling results were then interfaced with the regulatory network and metabolic model via the COBRApy project (http://opencobra.sourceforge.net/openCOBRA), iPython notebook [51], and in-house databases.

## Supporting Information

Figure S1 Workflow overview of the experimental and computational analysis process. An integrated and iterative loop was used to generate the integrated regulatory and metabolic analysis.
(PDF)

Figure S2 We performed a detailed comparison of the discrepancies between the ChIP data for ArcA and Fnr generated in this study vs. the ChIP data generated in the studies by Park et al. and Myers et al. This comparison is only performed for data generated under fermentative conditions as no other comparable conditions were assayed in the Park et al. and Myers et al. studies. The overall conclusion can be seen that our ArcA data is very similar but our Fnr data has significant differences. All of the code and results for this curation can be viewed at http://nbviewer.ipython.org/gist/steve-federowicz/aa44c9d8add955f4ada7 for Fnr and http://nbviewer.ipython.org/gist/steve-federowicz/1c5017c6ce419234019a for ArcA.
(PDF)

Figure S3 We performed a detailed comparison of the expression data for ***Δ*** *fnr* and ***Δ*** *arcA* strains compared to a wild type strain that were generated in our study versus that generated by the studies of Park et al. and Myers et al. This comparison is only performed for data generated under fermentative conditions as no other comparable conditions were assayed in the Park et al. and Myers et al. studies. The overall conclusion here is that most of the differences in each case were due to genes that were either not expressed or lowly expressed in our data. These differences can be primarily attributed to different measurement technologies used for gene expression measurement. We used affymetrix arrays throughout this study which generally do not have as high of a dynamic range as RNAseq or Nimblegen tiling arrays used in the studies of Park et al. and Myers et al. However, there is still a slight bias towards our ArcA data having reasonably similarity but our Fnr showing noticeable differences. All of the code and results for this curation can be viewed at http://nbviewer.ipython.org/gist/steve-federowicz/8c0e96ac208264e623b9 for Fnr and http://nbviewer.ipython.org/gist/steve-federowicz/05659c90b49abc049a42 for ArcA.
(PDF)

Figure S4 We performed a detailed comparison of the direct regulatory targets for ArcA and Fnr between our study and the studies of Park et al. and Myers et al. Direct regulatory targets are defined as genes that contain an upstream ChIP binding region along with significant differential gene expression between the knockout TF strain and a wild type strain. These comparisons show that for ArcA, the 21 discrepancies can be almost uniformly attributed to noise in highthroughput data in which some solid information exists, but ultimately falls below stringent cutoffs. A similar picture also emerges for Fnr with almost every discrepancy containing some type of comparable data in our study. All of the code and results for this curation can be viewed at http://nbviewer.ipython.org/gist/steve-federowicz/1cbb68842ab0a0571ff0 for Fnr and http://nbviewer.ipython.org/gist/steve-federowicz/f2b3d25f114914147c81 for ArcA.
(PDF)

Figure S5 Regulation of fluxes around key metabolic intermediates is quantified via the integration of computational sampling of the iJ01366 metabolic network and experimentally generated regulation data. Twenty-four different metabolites are profiled, including 12/13 biomass precursors, 9/9 primary electron donors, and the three primary electron carriers, H+, NADH, and NADPH. Each node map diagram shows the split between the amount of regulated vs. unregulated flux that goes into the production or consumption of each metabolite. The notable pattern is repression of the consumption and often production upon a shift to nitrate respiratory conditions. This occurs primarily as a means of negative feedback on the flux through these core nodes. In fact these diagrams fail to show that under fermentative conditions these same fluxes through core nodes are even more highly repressed. This occurs because the metabolic network at optimality is already in line with the regulation, and hence does not carry flux through many of the reactions that are shown to be repressed under nitrate respiratory conditions. This result led us to make the scatter plot of Figure 5A which more clearly displays the higher degree of repression in fermentation vs. nitrate respiratory conditions along with deactivation through the shift. All data tables and associated code is available at http://nbviewer.ipython.org/ea455904c0d7cda4bfba.
(PDF)

Figure S6 We compared C-13 MFA derived flux values [36] gathered for wild type strains and ***Δ*** *fnr* or ***Δ*** *arcA* strains under partially fermentative glucose batch growth. It can be seen that deletion of *arcA* does cause de-repression of the key catabolic fluxes of the TCA cycle. This causes less flux to be directed towards the fermentative chemiosmotic pathways ultimately wasting energy.
(PDF)

Figure S7 Transport coupled redox balancing. After sampling the metabolic model and determining all reactions that produce or consume NADH, we identified only 5 reactions that carried flux and were not regulated by ArcA of Fnr. We found that one encoded *fre*, a constitutively expressed NAD generation enzyme, and the other four, *serA*, *tyrA*, *metF*, and *hisD* all encode amino acid biosynthetic enzymes. We then took into consideration a puzzling finding of newly discovered and highly significant regulation of amino acid transporters for serine, tyrosine, methionine and histidine. We noticed that for *serA* and *tyrA* in particular, the NADH generating reactions were the subject of end product inhibition by serine and tyrosine. Thus we can hypothesize that activation of the uptake transporters for these amino acids will cause feedback inhibition of the enzymes and thus maintain the expression of critical metabolic enzymes while simultaneously modulating their redox related contributions.
(PDF)

Figure S8 Causative classification of genes differentially expressed log two fold between a wild type and ***Δ*** *arcA* or ***Δ*** *fnr* strain under fully fermentative conditions. After deletion of the *arcA* and *fnr* transcription factor genes, 148 and 169 genes are differentially expressed under anaerobic conditions. We then trace the regulatory network to explain the regulation of these genes. 63 and 47 are shown to be directly regulated through binding of the TFs in the ChIP-chip data. Another 48 and 60 genes are indirectly regulated via secondary network effects (Regulation by a local TF that is directly regulated by ArcA or Fnr). Finally the last three categories represent genes involved in the stress response, genes of unknown function, and other metabolic genes. Differentially regulated genes that are primarily stress response genes may represent variability in culture conditions or unknown regulatory interactions. Uncharacterized and metabolic genes likely represent unknown regulatory links.
(PDF)

Table S1 ArcA-associated regions under fermentative conditions identified by ChIP-chip analysis and its regulatory effect on the target operons determined by expression profiles. This table summarizes the results of ChIP-chip experiments to determine the genome-wide locations of DNA targets for ArcA binding in exponential phase E. coli cells growing in strictly anaerobic minimal media conditions. First and second columns indicate identified ArcA-binding peaks (Start: left-end peak position, End: right-end peak position). The third column indicates the log2 ratio of each ArcA-binding peak.
(PDF)

Table S2 ArcA-associated regions under nitrate respiratory conditions identified by ChIP-chip analysis and its regulatory effect on the target operons determined by expression profiles. This table summarizes the results of ChIP-chip experiments to determine the genome-wide locations of DNA targets for ArcA binding in exponential phase *E. coli* cells growing in strictly anaerobic minimal media with the addition of 20 mm KNO3. First and second columns indicate identified ArcA-binding peaks (Start: left-end peak position, End: right-end peak position). The third column indicates the log2 ratio of each ArcA-binding peak.
(PDF)

Table S3 Fnr-associated regions under fermentative conditions identified by ChIP-chip analysis and its regulatory effect on the target operons determined by expression profiles. This table summarizes the results of ChIP-chip experiments to determine the genome-wide locations of DNA targets for Fnr binding in exponential phase E. coli cells growing in strictly anaerobic minimal media conditions. First and second columns indicate identified Fnr-binding peaks (Start: left-end peak position, End: right-end peak position). The third column indicates the log2 ratio of each Fnr-binding peak.
(PDF)

Table S4 Fnr-associated regions under nitrate respiratory conditions identified by ChIP-chip analysis and its regulatory effect on the target operons determined by expression profiles. This table summarizes the results of ChIP-chip experiments to determine the genome-wide locations of DNA targets for Fnr binding in exponential phase *E. coli* cells growing in strictly anaerobic minimal media with the addition of 20 mm KNO3. First and second columns indicate identified Fnr-binding peaks (Start: left-end peak position, End: right-end peak position). The third column indicates the log2 ratio of each Fnr-binding peak.
(PDF)

Table S5 ArcA motifs found underneath experimentally determined ChIP binding regions. First column is the center position of the peak (averaged for peaks occuring under both fermentative and nitrate respiratory conditions).
(PDF)

Table S6 Fnr motifs found underneath experimentally determined ChIP binding regions. First column is the center position of the peak (averaged for peaks occuring under both fermentative and nitrate respiratory conditions).
(PDF)

Table S7 Mean flux values above .1 mmol/GDWH across all sampling points under anaerobic conditions. This table shows all reactions, whether or not they are directly regulated by ArcA or Fnr, their mean flux values, the percent of the total flux that this flux values corresponds too, and the list of genes associated with the reaction. For each reaction the regulation column is TRUE if at least one gene is directly regulated by ArcA or Fnr. The total percent of flux regulated can then be calculated by summing across all flux values which are regulated and dividing by the total.
(PDF)

Table S8 Mean flux values above .1 mmol/GDWH across all sampling points under nitrate conditons. This table shows all reactions, whether or not they are directly regulated by ArcA or Fnr, their mean flux values, the percent of the total flux that this flux values corresponds too, and the list of genes associated with the reaction. For each reaction the regulation column is TRUE if at least one gene is directly regulated by ArcA or Fnr. The total percent of flux regulated can then be calculated by summing across all flux values which are regulated and dividing by the total.
(PDF)

Table S9 Regulation of fluxes around key metabolites in fermentative conditions. This table shows all of the biomass precursor, electron donor, and electron carrier molecules along with the associated flux amounts in which they are produced or consumed and the amount of this flux which is activated or repressed by ArcA and Fnr.
(PDF)

Table S10 Regulation of fluxes around key metabolites in nitrate respiratory conditions. This table shows all of the biomass precursor, electron donor, and electron carrier molecules along with the associated flux amounts in which they are produced or consumed and the amount of this flux which is activated or repressed by ArcA and Fnr.
(PDF)

Table S11 This table shows the relative flux levels and differential transcriptional regulation values for reactions that differed by at least .25 mmol/GDWh mean sampled flux units between fermentative and nitrate respiratory conditions. This table contains 91 gene-associated reactions of which 89 are regulated by ArcA or Fnr. The first column is the COBRA reaction ID followed by the mean flux value in fermentative (fermen.) and nitrate respiratory (nitrate) conditions. The columns for Fermentative regulation and Nitrate regulation are the max absolute value levels of regulation (Fig. 3) cause by ArcA or Fnr under that condition across all genes associated with the metabolic reaction. The flux difference and regulation difference is always the value of the nitrate condition minus the value of the fermentation condition. The plot in figure 5c is between the last two columns of this table.
(PDF)

Table S12 Regulation of altered reactions based on sampling of flux solutions between fermentative and nitrate respiratory conditions. After calculating the set of reactions which differ in their flux values between fermentative and nitrate respiratory conditions we sought to understand how many of these reactions were regulated by ArcA and Fnr. Altered reactions describes the total number of reactions which differ between the conditions by the flux cutoff (e.g. 91 reactions differ between the two conditions by at least .25 mmol/GDW-h). Of these 91 reactions, 40 are directly regulated by ArcA or Fnr and another 49 are indirectly regulated.
(PDF)

Table S13 List of all transcription factors found in RegulonDB that map to metabolites in the iJO1366 metabolic model.
(PDF)

Text S1 Transport coupled redox balancing as shown in Fig. S4 is explained in greater detail. Briefly, only 5 genes are found that encode for reactions which produce NAD(P)H and are not regulated by ArcA or Fnr. Interestingly, 4/5 of these genes are amino acid biosynthetic enzymes. Two of these enzymes in particular, *serA* and *tyrA*, are feedback inhibited by serine and tyrosine respectively. Thus, as shown in Figure S4 we are able to corroborate dramatic regulation of the *sstT* serine transporter and the *aroP* tyrosine transporter with feedback inhibition of these critical biosynthetic enzymes. Under this regulatory scheme, serine and tyrosine would be produced at the expense of critical redox potential but immediately shut down if any serine or tyrosine can be scavenged exogenously.
(DOC)
