# Supplementary figures and images for: Determining the Control Circuitry of Redox Metabolism at the Genome-Scale
Source: PLoS Genet. 2014 Apr 3;10(4):e1004264. doi: 10.1371/journal.pgen.1004264 (PMC3974632; doi:10.1371/journal.pgen.1004264)

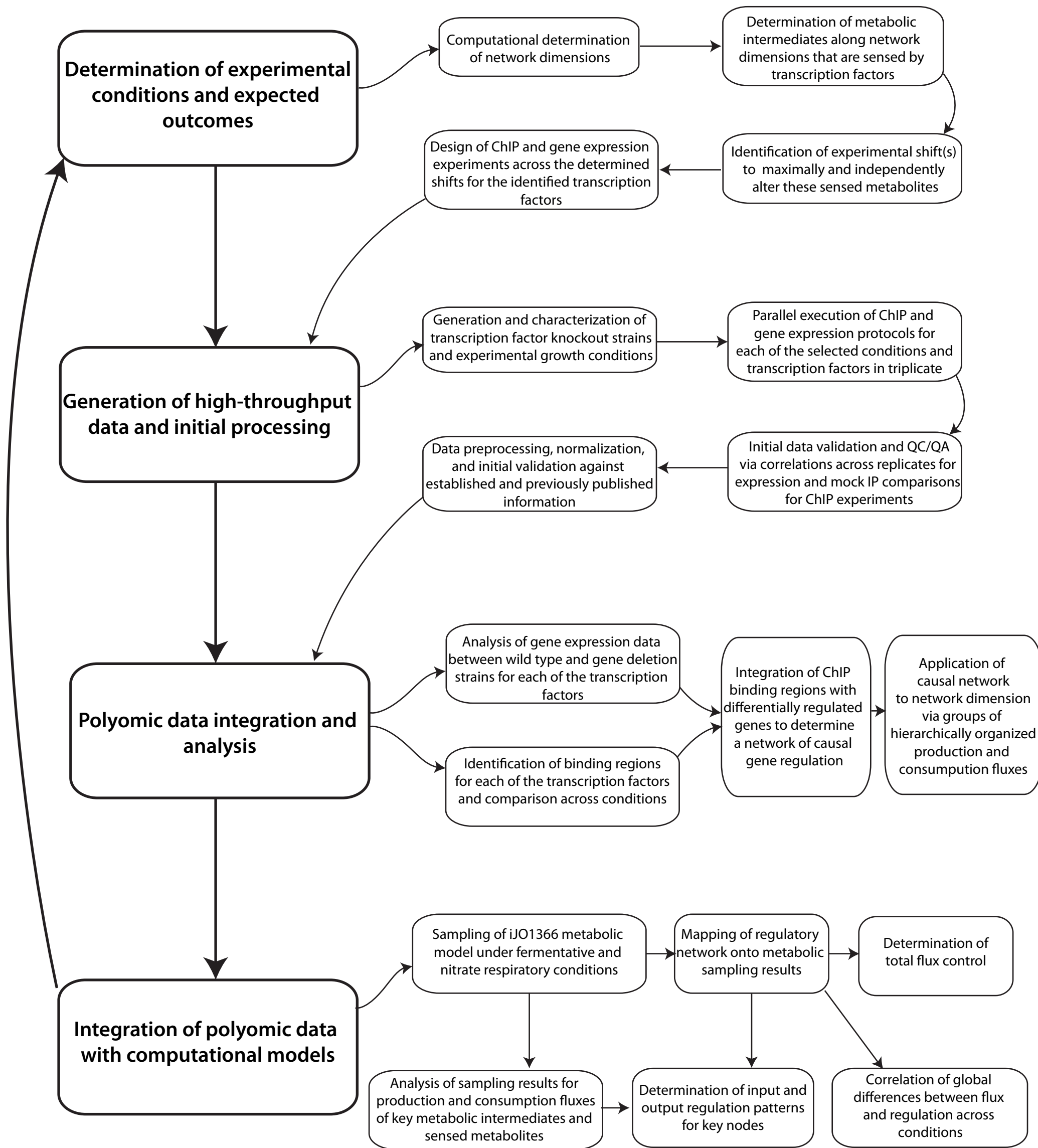

Supplement: Figure S1 — Workflow overview of the experimental and computational analysis process. An integrated and iterative loop was used to generate the integrated regulatory and metabolic analysis. (PDF) [file pgen.1004264.s001.pdf]

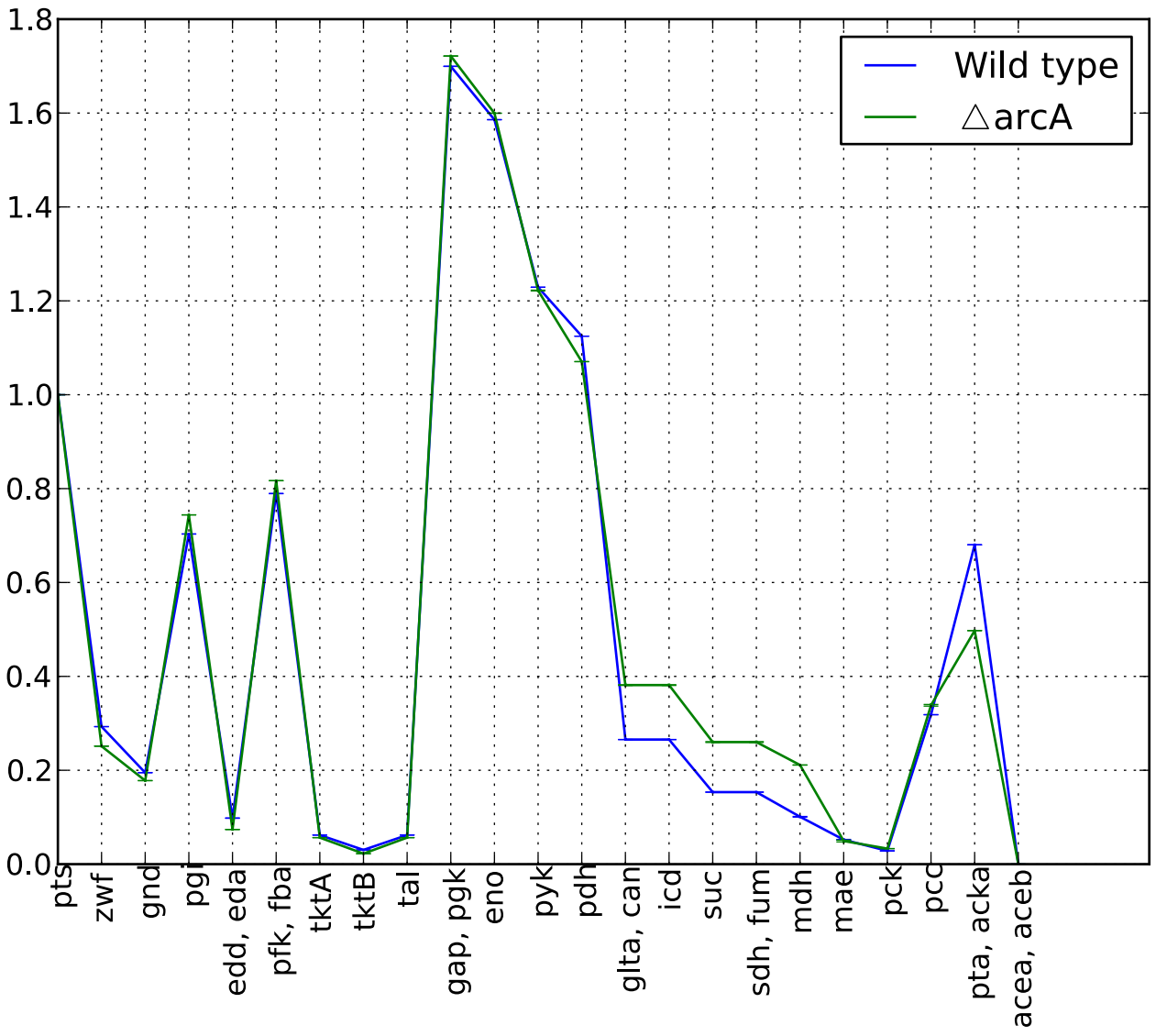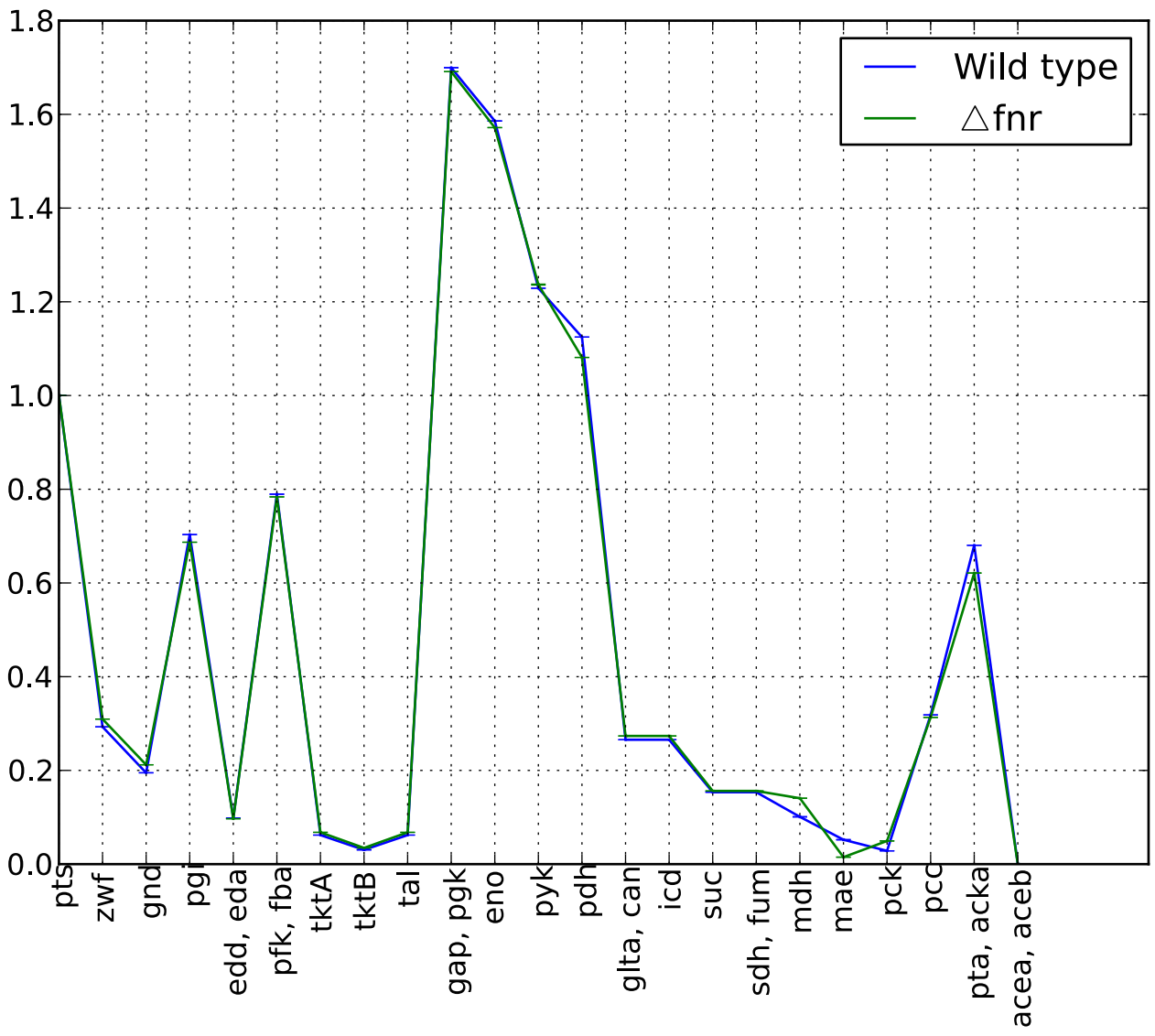

Supplement: Figure S6 — We compared C-13 MFA derived flux values [36] gathered for wild type strains and Δ fnr or Δ arcA strains under partially fermentative glucose batch growth. It can be seen that deletion of arcA does cause de-repression of the key catabolic fluxes of the TCA cycle. This causes less flux to be directed towards the fermentative chemiosmotic pathways ultimately wasting energy. (PDF) [file pgen.1004264.s006.pdf]

# Cytoplasm

## Periplasm

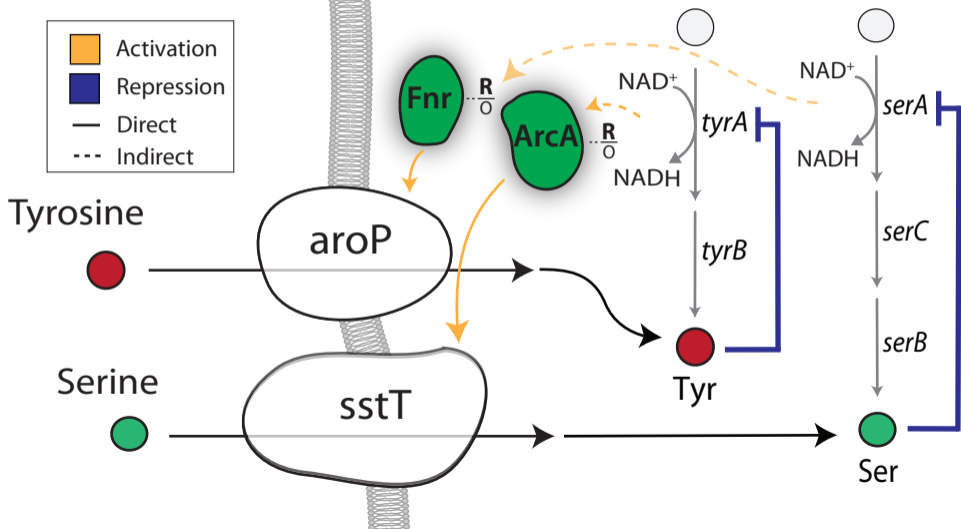

Supplement: Figure S7 — Transport coupled redox balancing. After sampling the metabolic model and determining all reactions that produce or consume NADH, we identified only 5 reactions that carried flux and were not regulated by ArcA of Fnr. We found that one encoded fre, a constitutively expressed NAD generation enzyme, and the other four, serA, tyrA, metF, and hisD all encode amino acid biosynthetic enzymes. We then took into consideration a puzzling finding of newly discovered and highly significant regulation of amino acid transporters for serine, tyrosine, methionine and histidine. We noticed that for serA and tyrA in particular, the NADH generating reactions were the subject of end product inhibition by serine and tyrosine. Thus we can hypothesize that activation of the uptake transporters for these amino acids will cause feedback inhibition of the enzymes and thus maintain the expression of critical metabolic enzymes while simultaneously modulating their redox related contributions. (PDF) [file pgen.1004264.s007.pdf]
