# Supplementary material for: Determining the Control Circuitry of Redox Metabolism at the Genome-Scale
Source: PLoS Genet. 2014 Apr 3;10(4):e1004264. doi: 10.1371/journal.pgen.1004264 (PMC3974632; doi:10.1371/journal.pgen.1004264)
Supplement: Figure S2 — We performed a detailed comparison of the discrepancies between the ChIP data for ArcA and Fnr generated in this study vs. the ChIP data generated in the studies by Park et al. and Myers et al. This comparison is only performed for data generated under fermentative conditions as no other comparable conditions were assayed in the Park et al. and Myers et al. studies. The overall conclusion can be seen that our ArcA data is very similar but our Fnr data has significant differences. All of the code and results for this curation can be viewed at http://nbviewer.ipython.org/gist/steve-federowicz/aa44c9d8add955f4ada7 for Fnr and http://nbviewer.ipython.org/gist/steve-federowicz/1c5017c6ce419234019a for ArcA. (PDF) [file pgen.1004264.s002.pdf]

ArcA ChIP peak discrepancies

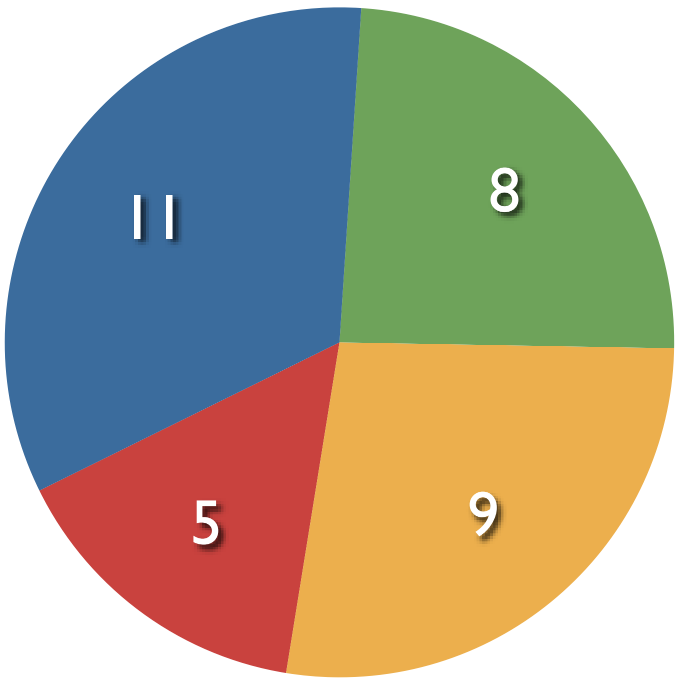

- 33/146 ChIP peaks not found in our data

ChIP peak (one replicate)
Weak peaks
Phage/sRNA/rRNA related
No signal

Fnr ChIP peak discrepancies

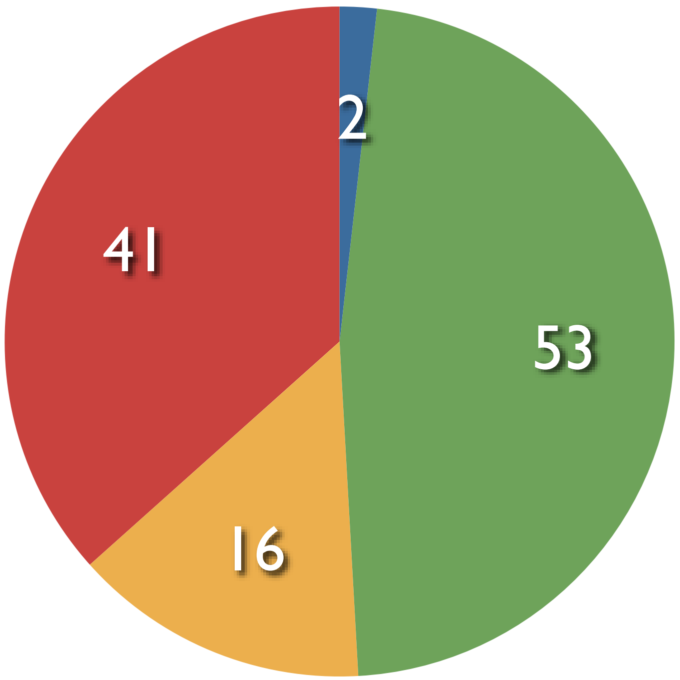

- 112/226 ChIP peaks not found in our data

CHIP peak (one replicate)
Weak peaks
Phage/sRNA/rRNA related
No signal
