# Supplementary material for: Determining the Control Circuitry of Redox Metabolism at the Genome-Scale
Source: PLoS Genet. 2014 Apr 3;10(4):e1004264. doi: 10.1371/journal.pgen.1004264 (PMC3974632; doi:10.1371/journal.pgen.1004264)
Supplement: Figure S3 — We performed a detailed comparison of the expression data for Δ fnr and Δ arcA strains compared to a wild type strain that were generated in our study versus that generated by the studies of Park et al. and Myers et al. This comparison is only performed for data generated under fermentative conditions as no other comparable conditions were assayed in the Park et al. and Myers et al. studies. The overall conclusion here is that most of the differences in each case were due to genes that were either not expressed or lowly expressed in our data. These differences can be primarily attributed to different measurement technologies used for gene expression measurement. We used affymetrix arrays throughout this study which generally do not have as high of a dynamic range as RNAseq or Nimblegen tiling arrays used in the studies of Park et al. and Myers et al. However, there is still a slight bias towards our ArcA data having reasonably similarity but our Fnr showing noticeable differences. All of the code and results for this curation can be viewed at http://nbviewer.ipython.org/gist/steve-federowicz/8c0e96ac208264e623b9 for Fnr and http://nbviewer.ipython.org/gist/steve-federowicz/05659c90b49abc049a42 for ArcA. (PDF) [file pgen.1004264.s003.pdf]

Fnr expression discrepancies

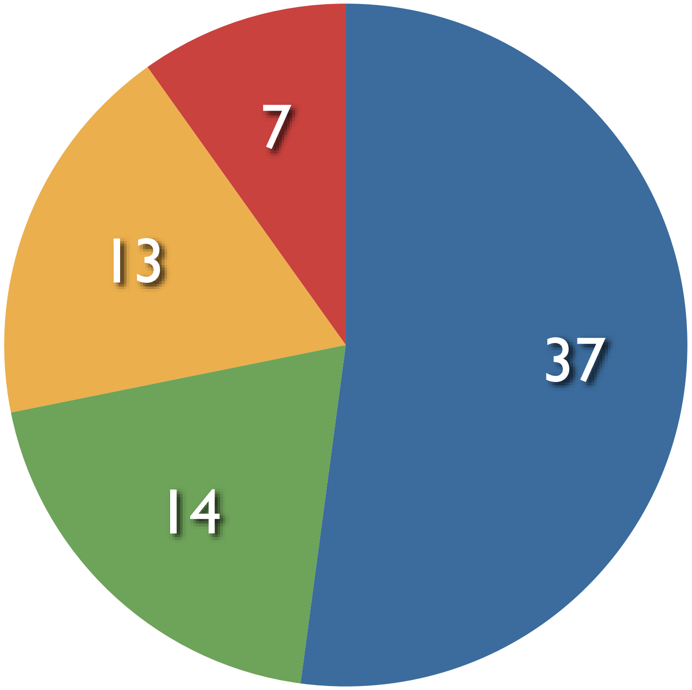

•71/258 differential expression measurements not found in our data

- Not expressed or lowly expressed
- Not measured on array
- Not significant (missed)
- Just above cutoff

ArcA expression discrepancies

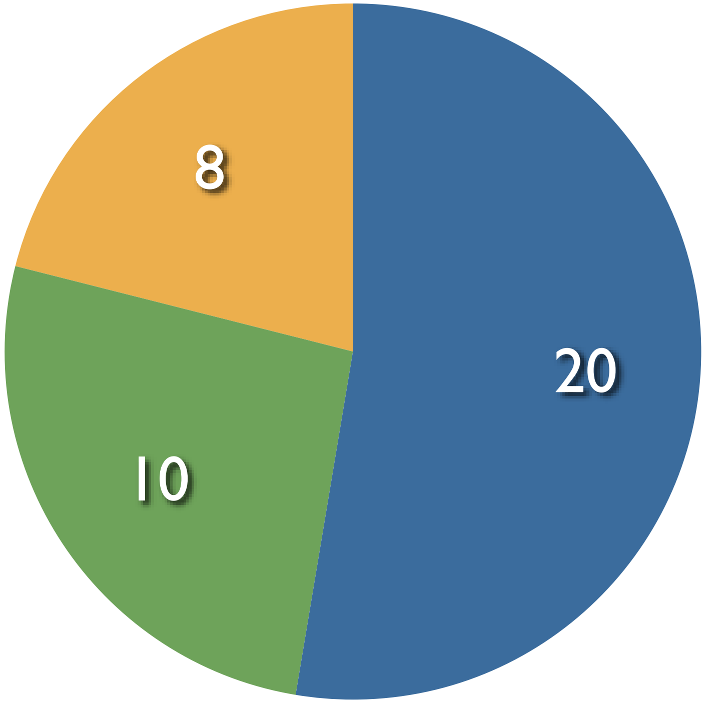

•38/229 differential expression measurements not found in our data

- Not expressed or lowly expressed
- Not measured on array
- Not significant (missed)
