# Supplementary material for: Determining the Control Circuitry of Redox Metabolism at the Genome-Scale
Source: PLoS Genet. 2014 Apr 3;10(4):e1004264. doi: 10.1371/journal.pgen.1004264 (PMC3974632; doi:10.1371/journal.pgen.1004264)
Supplement: Figure S4 — We performed a detailed comparison of the direct regulatory targets for ArcA and Fnr between our study and the studies of Park et al. and Myers et al. Direct regulatory targets are defined as genes that contain an upstream ChIP binding region along with significant differential gene expression between the knockout TF strain and a wild type strain. These comparisons show that for ArcA, the 21 discrepancies can be almost uniformly attributed to noise in highthroughput data in which some solid information exists, but ultimately falls below stringent cutoffs. A similar picture also emerges for Fnr with almost every discrepancy containing some type of comparable data in our study. All of the code and results for this curation can be viewed at http://nbviewer.ipython.org/gist/steve-federowicz/1cbb68842ab0a0571ff0 for Fnr and http://nbviewer.ipython.org/gist/steve-federowicz/f2b3d25f114914147c81 for ArcA. (PDF) [file pgen.1004264.s004.pdf]

Fnr direct regulation discrepancies

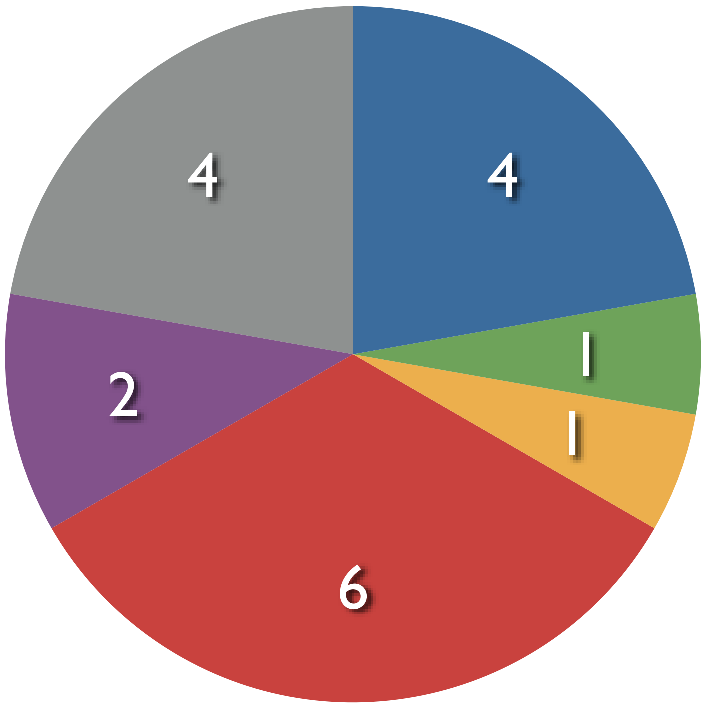

- 18/53 direct regulatory targets not found in our data

ArcA direct regulation discrepancies

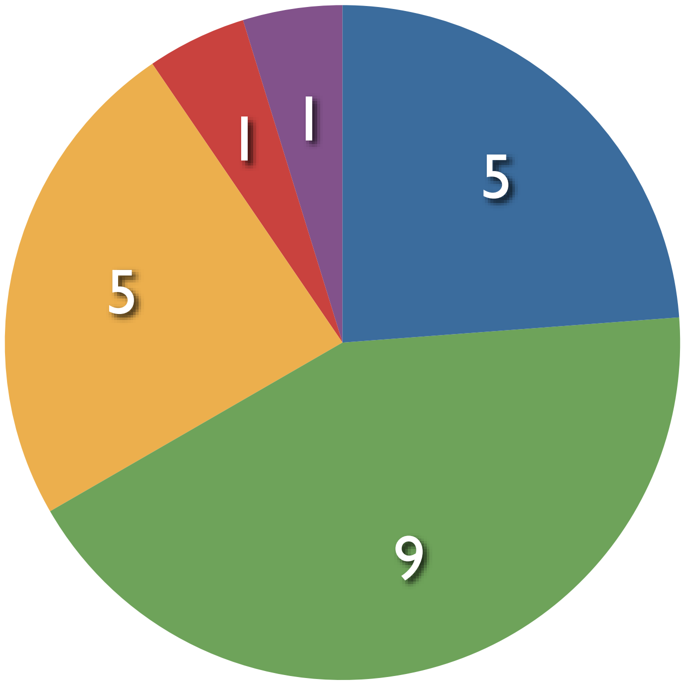

- 21/85 direct regulatory targets not found in our data
