# Supplementary material for: Determining the Control Circuitry of Redox Metabolism at the Genome-Scale
Source: PLoS Genet. 2014 Apr 3;10(4):e1004264. doi: 10.1371/journal.pgen.1004264 (PMC3974632; doi:10.1371/journal.pgen.1004264)
Supplement: Figure S5 — Regulation of fluxes around key metabolic intermediates is quantified via the integration of computational sampling of the iJ01366 metabolic network and experimentally generated regulation data. Twenty-four different metabolites are profiled, including 12/13 biomass precursors, 9/9 primary electron donors, and the three primary electron carriers, H+, NADH, and NADPH. Each node map diagram shows the split between the amount of regulated vs. unregulated flux that goes into the production or consumption of each metabolite. The notable pattern is repression of the consumption and often production upon a shift to nitrate respiratory conditions. This occurs primarily as a means of negative feedback on the flux through these core nodes. In fact these diagrams fail to show that under fermentative conditions these same fluxes through core nodes are even more highly repressed. This occurs because the metabolic network at optimality is already in line with the regulation, and hence does not carry flux through many of the reactions that are shown to be repressed under nitrate respiratory conditions. This result led us to make the scatter plot of Figure 5A which more clearly displays the higher degree of repression in fermentation vs. nitrate respiratory conditions along with deactivation through the shift. All data tables and associated code is available at http://nbviewer.ipython.org/ea455904c0d7cda4bfba. (PDF) [file pgen.1004264.s005.pdf]

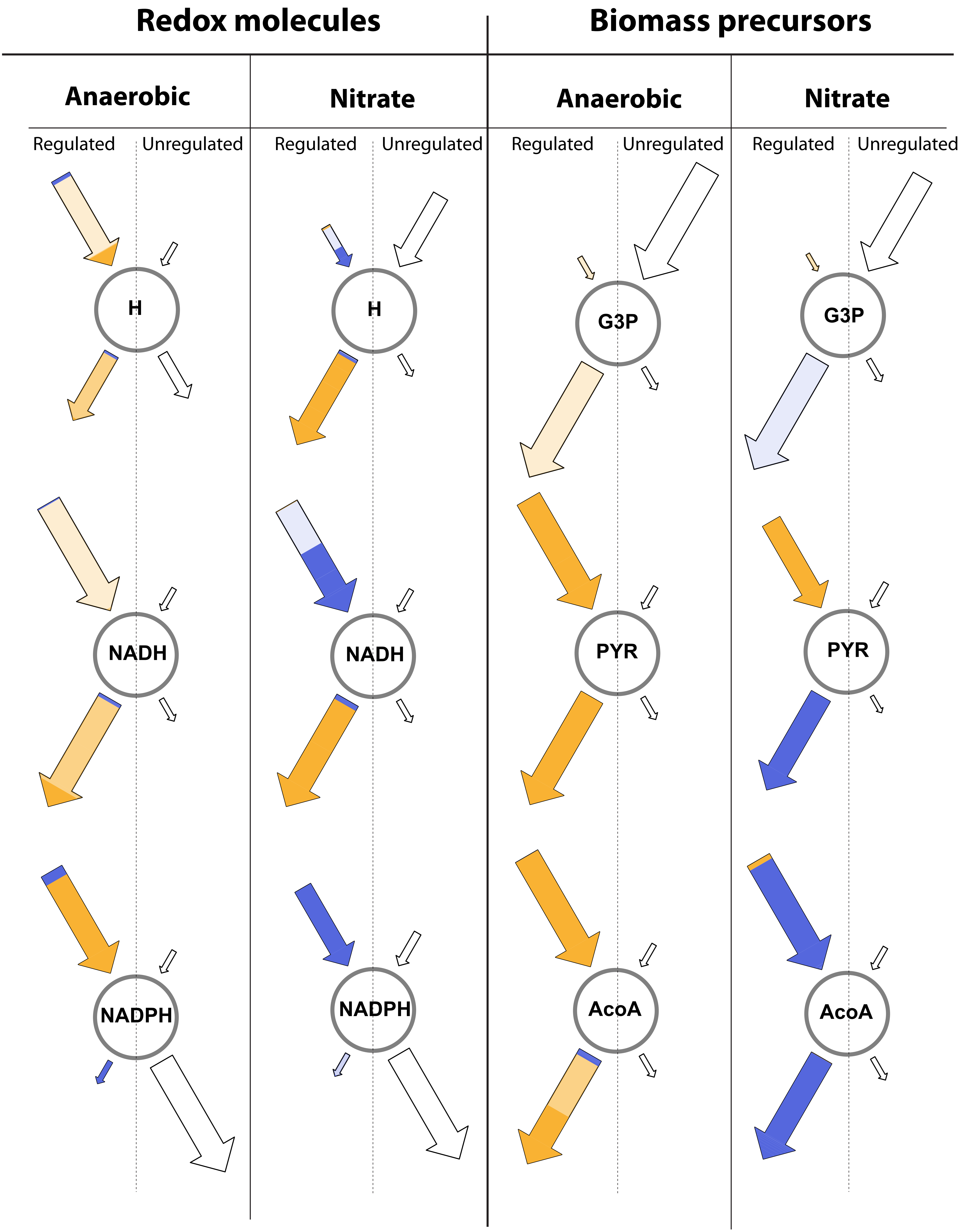

Redox molecules
Biomass precursors
Anaerobic
Nitrate
Anaerobic
Nitrate
Regulated
Unregulated
Regulated
Unregulated
Regulated
Unregulated
Regulated
Unregulated
H
H
G3P
G3P
NADH
NADH
PYR
PYR
NADPH
NADPH
AcoA
AcoA

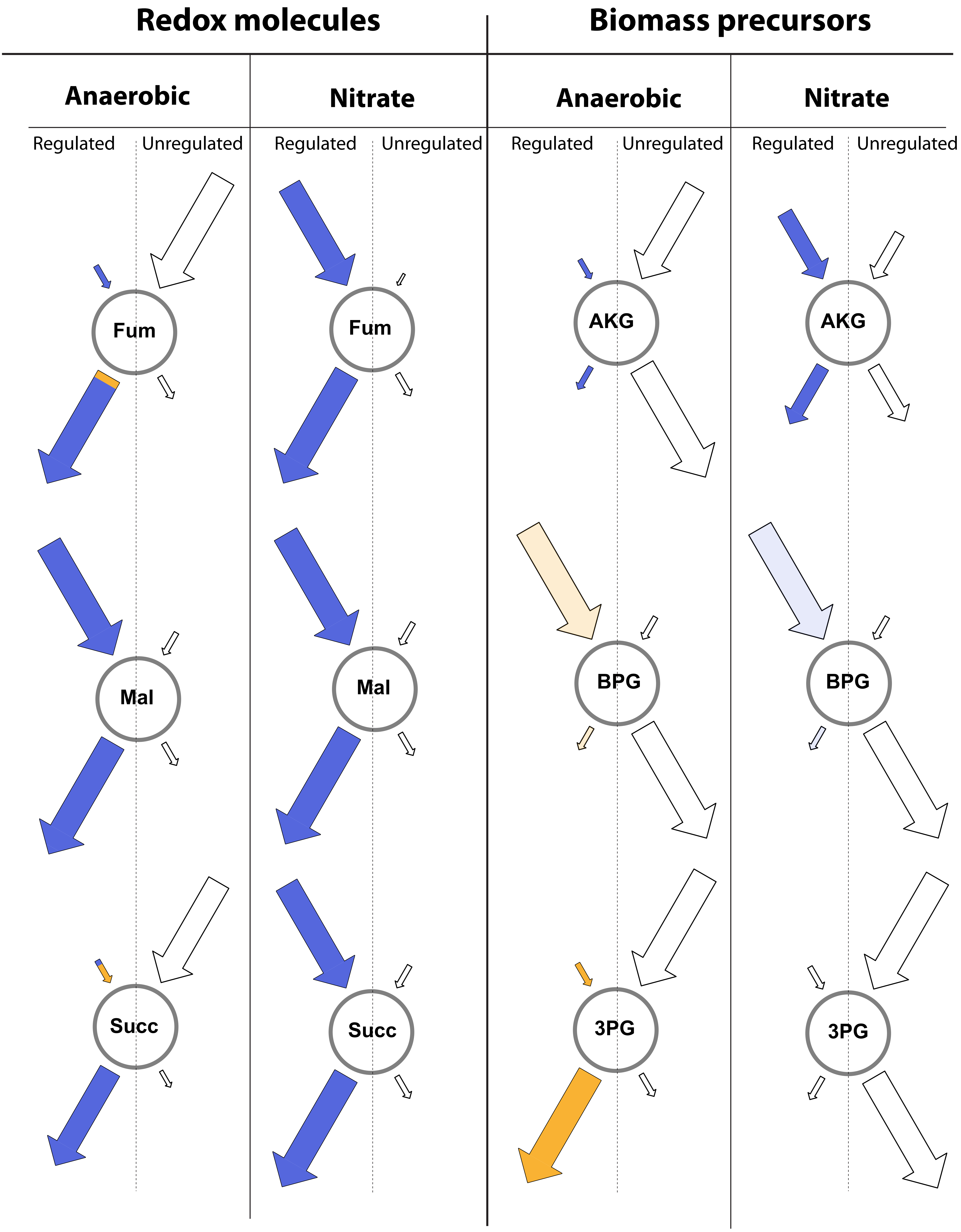

Redox molecules
Biomass precursors
Anaerobic
Nitrate
Anaerobic
Nitrate
Regulated
Unregulated
Regulated
Unregulated
Regulated
Unregulated
Regulated
Unregulated
Fum
Fum
AKG
AKG
Mal
Mal
BPG
BPG
Succ
Succ
3PG
3PG

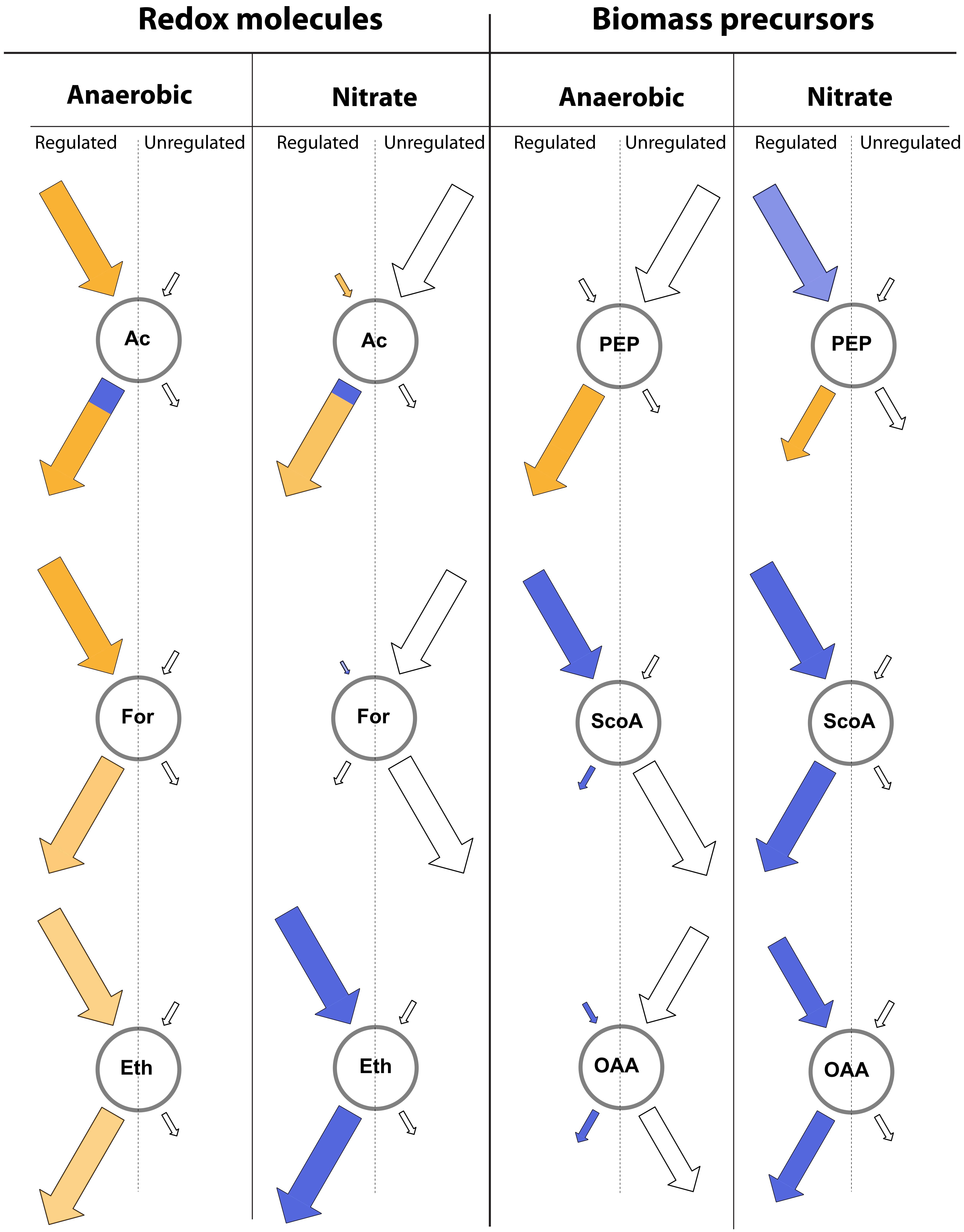
Redox molecules
Biomass precursors
Anaerobic
Nitrate
Anaerobic
Nitrate
Regulated
Unregulated
Regulated
Unregulated
Regulated
Unregulated
Regulated
Unregulated
Ac
Ac
PEP
PEP
For
For
ScoA
ScoA
Eth
Eth
OAA
OAA
