# Supplementary material for: Determining the Control Circuitry of Redox Metabolism at the Genome-Scale
Source: PLoS Genet. 2014 Apr 3;10(4):e1004264. doi: 10.1371/journal.pgen.1004264 (PMC3974632; doi:10.1371/journal.pgen.1004264)
Supplement: Figure S8 — Causative classification of genes differentially expressed log two fold between a wild type and Δ arcA or Δ fnr strain under fully fermentative conditions. After deletion of the arcA and fnr transcription factor genes, 148 and 169 genes are differentially expressed under anaerobic conditions. We then trace the regulatory network to explain the regulation of these genes. 63 and 47 are shown to be directly regulated through binding of the TFs in the ChIP-chip data. Another 48 and 60 genes are indirectly regulated via secondary network effects (Regulation by a local TF that is directly regulated by ArcA or Fnr). Finally the last three categories represent genes involved in the stress response, genes of unknown function, and other metabolic genes. Differentially regulated genes that are primarily stress response genes may represent variability in culture conditions or unknown regulatory interactions. Uncharacterized and metabolic genes likely represent unknown regulatory links. (PDF) [file pgen.1004264.s008.pdf]

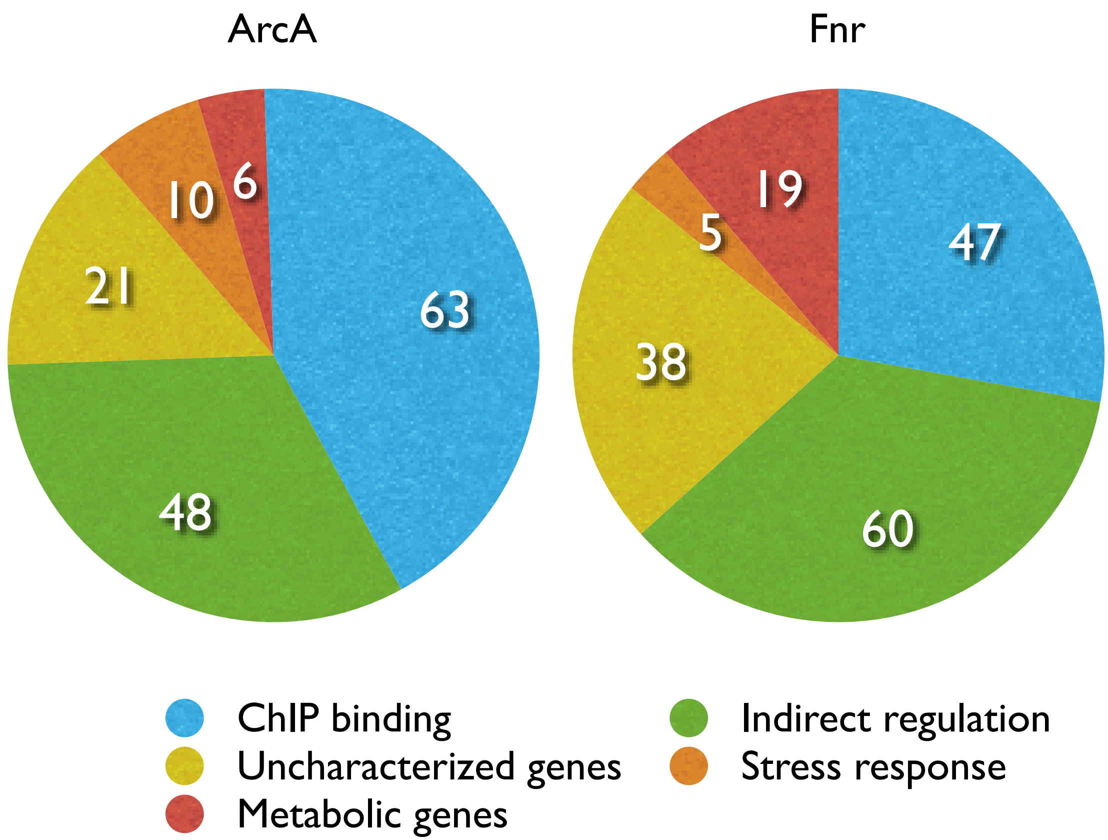
ArcA
Fnr
63
48
21
10
6
47
60
38
5
19
ChIP binding
Uncharacterized genes
Metabolic genes
Indirect regulation
Stress response
