# Supplementary material for: Determining the Control Circuitry of Redox Metabolism at the Genome-Scale
Source: PLoS Genet. 2014 Apr 3;10(4):e1004264. doi: 10.1371/journal.pgen.1004264 (PMC3974632; doi:10.1371/journal.pgen.1004264)
Supplement: Table S1 — ArcA-associated regions under fermentative conditions identified by ChIP-chip analysis and its regulatory effect on the target operons determined by expression profiles. This table summarizes the results of ChIP-chip experiments to determine the genome-wide locations of DNA targets for ArcA binding in exponential phase E. coli cells growing in strictly anaerobic minimal media conditions. First and second columns indicate identified ArcA-binding peaks (Start: left-end peak position, End: right-end peak position). The third column indicates the log2 ratio of each ArcA-binding peak. (PDF) [file pgen.1004264.s009.pdf]

Supplementary Table 1. ArcA-associated regions under fermentative conditions identified by ChIP-chip analysis and its regulatory effect on the target operons determined by expression profiles. This table summarizes the results of ChIP-chip experiments to determine the genome-wide locations of DNA targets for ArcA binding in exponential phase E. coli cells growing in strictly anaerobic minimal media conditions. First and second columns indicate identified ArcA-binding peaks (Start: left-end peak position, End: right-end peak position). The third column indicates the log2 ratio of each ArcA-binding peak.

Peak start	Peak end	Occupancy	Strand	Operon	Regulation	Regulon DB
41673	42043	6.21	-	[caiA, caiB, caiC, caiD, caiE, caiT]	N/D	Known
127592	127887	3.17	+	[lpd]	Repression	Known
131051	131535	14.86	+	[acnB]	Repression	Known
243102	243589	9.24	-	[fadE]	Repression	Known
328313	328569	7.48	-	[betA, betB, betI]	Repression	Known
367666	368120	15	-	[lacI, mhpR]	Repression	
450610	451407	42.28	-	[cyoA, cyoB, cyoC, cyoD, cyoE]	Repression	Known
727996	728351	4.57	-	[kdpA, kdpB, kdpC, kdpF]	Repression	
727996	728351	4.57	+	[ybfA]	Activation	
754078	754422	12.18	+	[sdhA, sdhB, sdhC, sdhD, sucA, sucB, sucC, sucD]	Repression	Known
754078	754422	12.18	-	[gltA]	Repression	Known
770022	770651	20.42	+	[cydA, cydB]	N/D	Known
858148	858715	15.04	-	[ybiU]	N/D	
892526	892922	10.03	+	[potF, potG, potH, potI]	Repression	
922324	922705	4.74	+	[clpA, clpS]	Repression	
930002	930806	35.11	-	[cydC, cydD]	Activation	Known
989731	990093	9.93	-	[pncB]	Activation	
989731	990093	9.93	+	[pepN]	Repression	
1072984	1073676	26.12	+	[rutR]	N/D	
1072984	1073676	26.12	-	[rutA, rutB, rutC, rutD, rutE, rutF, rutG]	N/D	Known
1077976	1078513	16.17	+	[putP]	Repression	
1077976	1078513	16.17	-	[putA]	Repression	
1083942	1084376	9.1	+	[phoH]	Repression	
1118029	1118304	4.38	-	[yceI, yceJ]	N/D	
1165381	1165681	8.52	+	[ndh]	N/D	Known
1194055	1194546	11.94	+	[icd]	Repression	Known
1232191	1232571	4.11	-	[dsbB]	Activation	
1249988	1250500	13.65	+	[dhaR]	N/D	
1249988	1250500	13.65	-	[dhaK, dhaL, dhaM]	N/D	
1298414	1299077	9.99	+	[oppA, oppB, oppC, oppD, oppF]	Activation	Known
1358916	1359404	10.18	-	[puuA]	Repression	Known
1358916	1359404	10.18	+	[puuD, puuR]	Repression	Known
1386680	1387030	5.38	-	[tpx]	Repression	Known
1395321	1395688	3.95	+	[ynaJ]	Repression	
1445043	1445306	5.92	-	[feaR]	N/D	
1445043	1445306	5.92	+	[feaB]	N/D	
1451593	1452006	6.74	+	[paaA, paaB, paaC, paaD, paaE, paaF, paaG, paaH, paaI, paaJ, paaK]	N/D	
1451593	1452006	6.74	-	[maoC]	N/D	
1485972	1486451	8.72	+	[aldA]	Repression	Known
1520897	1521355	5.49	-	[yncD]	N/D	
1520897	1521355	5.49	+	[yncE]	N/D	
1560974	1561381	4.54	-	[ddpA, ddpB, ddpC, ddpD, ddpF, ddpX]	N/D	
1608401	1608967	7.33	-	[uxaB]	N/D	
1675896	1676296	3.44	-	[pntA, pntB]	Activation	
1679651	1680231	6.65	-	[ydgC]	N/D	
1679651	1680231	6.65	+	[rstA, rstB]	N/D	

1684671	1685000	4.13	-	[fumC]	N/D	Known
1686175	1686721	6.87	+	[manA]	Activation	
1686175	1686721	6.87	-	[fumA]	Repression	Known
1829769	1830203	14.16	-	[astA, astB, astC, astD, astE]	Repression	
1887540	1888069	13.12	-	[fadD]	Repression	Known
1976188	1976458	7.36	-	[flhC, flhD]	Activation	
1977346	1977771	13.36	+	[uspC]	N/D	
1977346	1977771	13.36	-	[insA-5, insB-5]	N/D	
1985963	1986293	4.31	-	[azuC, isrB, yecJ]	N/D	
1985963	1986293	4.31	+	[yecR]	N/D	
2238314	2238893	18.72	-	[mglA, mglB, mglC]	Repression	
2263947	2264426	6.57	+	[yeiQ]	N/D	
2275985	2276585	18.4	-	[yejG]	Repression	
2304730	2305072	4.37	-	[mqo]	Repression	
2310839	2311659	24.17	-	[ompC]	Activation	
2310839	2311659	24.17	+	[micF, rcsB, rcsD]	Activation	
2384877	2385185	6.48	+	[yfbM]	N/D	
2403052	2403517	8.02	-	[nuoA, nuoB, nuoC, nuoE, nuoF, nuoG, nuoH, nuoI, nuoJ, nuoK, nuoL, nuoM, nuoN]	Repression	Known
2425640	2426190	15.61	-	[argT, hisJ, hisM, hisP, hisQ]	Repression	
2458450	2458955	11.21	-	[fadI, fadJ, yfcZ]	Repression	
2496255	2496563	3.36	-	[yfdZ]	N/D	
2496255	2496563	3.36	+	[ypdA, ypdB, ypdC]	N/D	
2511143	2511538	6.89	+	[nupC]	Activation	
2561262	2561604	4.49	+	[yffQ, yffR]	N/D	
2642690	2643090	7.15	-	[ndk]	Repression	
2666749	2667128	8.22	-	[hcaR]	N/D	
2666749	2667128	8.22	+	[hcaB, hcaC, hcaD, hcaE, hcaF]	N/D	
2716446	2716865	5.62	-	[yfiF]	Activation	
2723572	2723872	3.48	-	[kgtP]	Repression	
2786701	2787319	25.66	+	[csiD, gabD, gabP, gabT, lhgO]	Repression	
2940428	2940878	11.1	+	[gcvB]	N/D	
2940428	2940878	11.1	-	[gcvA]	N/D	
3125935	3126447	15.57	+	[glcC]	Repression	
3125935	3126447	15.57	-	[glcA, glcB, glcD, glcE, glcF, glcG]	Repression	Known
3216895	3217770	38.1	+	[ygjG]	Repression	
3229662	3230012	8.17	+	[fadH]	N/D	Known
3237433	3237862	4.8	+	[sstT]	Activation	
3242567	3243093	5.39	+	[exuT]	N/D	
3242567	3243093	5.39	-	[uxaA, uxaC]	N/D	
3382293	3382689	5.46	-	[mdh]	Repression	Known
3523980	3524692	6.25	-	[nudE]	Repression	
3537747	3538156	5.09	+	[feoA, feoB, feoC]	N/D	
3559590	3560232	7.54	-	[glpE, glpG, glpR]	Repression	
3590034	3590717	13.69	-	[ugpA, ugpB, ugpC, ugpE, ugpQ]	Repression	
3663707	3664344	7.12	-	[gadW, gadX]	Activation	Known
3672095	3673091	58.62	+	[yhjE]	Repression	
3705835	3706180	5.51	-	[dppA, dppB, dppC, dppD, dppF]	Activation	
3710036	3710417	8.83	-	[yhjX]	N/D	
3737340	3737681	5.16	+	[avtA]	N/D	
3775016	3775341	4.38	+	[lldD, lldP, lldR]	N/D	Known
3839670	3839963	4.71	-	[nepI]	N/D	
3891618	3891908	3.95	+	[yieE, yieF]	N/D	
4001099	4001479	8.02	-	[yigF, yigG]	N/D	
4002474	4002970	4.49	-	[yigI]	Repression	
4002474	4002970	4.49	+	[pldA]	Activation	
4028847	4029206	6.02	+	[hemG, pepQ, trkH, yigZ]	conflict	

4028847	4029206	6.02	-	[fadA, fadB]	Repression	Known
4083326	4083676	3.15	-	[fdhE, fdoG, fdoH, fdoI]	Activation	
4158619	4159036	9.69	-	[sthA]	Repression	
4194200	4194700	7.06	-	[thiC, thiE, thiF, thiG, thiH, thiS]	Repression	
4250255	4250645	9.92	+	[ubiA, ubiC]	N/D	Known
4285048	4285577	16.88	+	[nrfA, nrfB, nrfC, nrfD, nrfE, nrfF, nrfG]	Activation	
4285048	4285577	16.88	-	[acs, actP, yjcH]	Repression	
4323037	4323628	7.91	-	[phnC, phnD, phnE, phnF, phnG, phnH, phnI, phnJ, phnK, phnL, phnM, phnN, phnO, phnP]	N/D	
4346670	4347185	6.82	-	[dcuB, fumB]	Activation	
4364658	4365208	5.87	-	[dcuA]	Activation	
4427459	4427784	8.55	+	[cycA]	N/D	
4560494	4560948	13.42	-	[yjiJ]	N/D	
4569465	4569782	6.6	+	[yjiS]	N/D	
4569465	4569782	6.6	-	[yjiR]	N/D	
4608883	4609308	4.19	+	[osmY]	Repression	
4638782	4638982	3.22	-	[arcA]	Activation	
