# Supplementary material for: Determining the Control Circuitry of Redox Metabolism at the Genome-Scale
Source: PLoS Genet. 2014 Apr 3;10(4):e1004264. doi: 10.1371/journal.pgen.1004264 (PMC3974632; doi:10.1371/journal.pgen.1004264)
Supplement: Table S2 — ArcA-associated regions under nitrate respiratory conditions identified by ChIP-chip analysis and its regulatory effect on the target operons determined by expression profiles. This table summarizes the results of ChIP-chip experiments to determine the genome-wide locations of DNA targets for ArcA binding in exponential phase E. coli cells growing in strictly anaerobic minimal media with the addition of 20 mm KNO3. First and second columns indicate identified ArcA-binding peaks (Start: left-end peak position, End: right-end peak position). The third column indicates the log2 ratio of each ArcA-binding peak. (PDF) [file pgen.1004264.s010.pdf]

Supplementary Table 2. ArcA-associated regions under nitrate respiratory conditions identified by ChIP-chip analysis and its regulatory effect on the target operons determined by expression profiles. This table summarizes the results of ChIP-chip experiments to determine the genome-wide locations of DNA targets for ArcA binding in exponential phase E. coli cells growing in strictly anaerobic minimal media with the addition of 20mm KNO3. First and second columns indicate identified ArcA-binding peaks (Start: left-end peak position, End: right-end peak position). The third column indicates the log2 ratio of each ArcA-binding peak.

Peak start	Peak end	Occupancy	Strand	Operon	Regulation	Regulon DB
22	205	2.63	+	[thrA, thrB, thrC, thrL]	Repression	
21121	21465	5.86	+	[fkpB, ileS, ispH, lspA, ribF]	Activation	
21121	21465	5.86	-	[rpsT]	Activation	
29489	30064	8.02	+	[carA, carB]	Activation	
41664	42181	7.87	-	[caiA, caiB, caiC, caiD, caiE, caiT]	Activation	Known
83468	83931	9.42	-	[leuA, leuB, leuC, leuD, leuL]	Activation	
127633	128251	6.51	+	[lpd]	Repression	Known
131272	131772	7.19	+	[acnB]	Repression	Known
131272	131772	7.19	-	[yacH]	N/D	
243079	243744	8.38	-	[fadE]	Repression	Known
243079	243744	8.38	+	[lpcA]	N/D	
367853	368107	5.65	-	[lacI, mhpR]	Repression	
367853	368107	5.65	+	[mhpA, mhpB, mhpC, mhpD, mhpE, mhpF]	Repression	
450221	451684	77.87	-	[cyoA, cyoB, cyoC, cyoD, cyoE]	Repression	Known
727885	728489	5.78	-	[kdpA, kdpB, kdpC, kdpF]	N/D	
727885	728489	5.78	+	[ybfA]	Activation	
753888	754426	19.63	+	[sdhA, sdhB, sdhC, sdhD, sucA, sucB, sucC, sucD]	Repression	Known
753888	754426	19.63	-	[gltA]	Repression	Known
769905	770563	14.61	+	[cydA, cydB]	Activation	Known
815916	816683	13.7	+	[moaA, moaB, moaC, moaD, moaE]	Repression	
815916	816683	13.7	-	[ybhK]	Activation	
840676	841126	7.62	-	[fiu, mcbA]	Repression	
857915	858999	49.88	-	[ybiU]	Repression	
862364	862943	8.59	-	[ybiW, ybiY]	N/D	
862364	862943	8.59	+	[fsaA]	Activation	
865743	866455	13.12	+	[gsiA, gsiB, gsiC, gsiD, iaaA]	Activation	
877089	877801	7.1	+	[bssR]	Repression	
877089	877801	7.1	-	[rimO]	Activation	
892368	893009	24.98	+	[potF, potG, potH, potI]	Repression	
917844	918319	7.05	+	[macA, macB]	Repression	
917844	918319	7.05	-	[ybjX]	Activation	
924976	925842	13.99	-	[infA, serW]	Activation	
930102	930635	12.21	-	[cydC, cydD]	Activation	Known
953235	954107	10.37	-	[focA, pflB]	Repression	Known
989551	989881	8.78	+	[pepN]	Repression	
989551	989881	8.78	-	[pncB]	N/D	
1014840	1015615	8.1	+	[rmf]	N/D	
1030559	1031021	8.71	-	[serT, yccA]	N/D	
1072946	1073509	12.52	-	[rutA, rutB, rutC, rutD, rutE, rutF, rutG]	N/D	Known
1072946	1073509	12.52	+	[rutR]	N/D	
1078134	1079013	11.09	+	[putP]	N/D	
1084046	1084376	4	+	[phoH]	N/D	
1145843	1146734	14.94	+	[fabD, fabG, fabH, plsX, psrD, rpmF, yceD]	Activation	
1150735	1151219	7.77	+	[acpP, fabF]	Activation	
1156739	1157531	9.54	+	[ptsG]	Activation	Known
1165276	1165541	3.22	+	[ndh]	N/D	Known
1194146	1194605	9.34	+	[icd]	Repression	Known
1194146	1194605	9.34	-	[nudJ, rluE]	N/D	

1232163	1232721	9.94	-	[dsbB]	Activation	
1358699	1359349	6.29	+	[puuD, puuR]	Repression	Known
1358699	1359349	6.29	-	[puuA]	N/D	Known
1386238	1387113	6.73	-	[tpx]	Repression	Known
1386238	1387113	6.73	+	[ycjG]	N/D	
1451672	1452114	4.29	+	[paaA, paaB, paaC, paaD, paaE, paaF, paaG, paaH, paaI, paaJ, paaK]	Repression	
1451672	1452114	4.29	-	[maoC]	N/D	
1485851	1486522	7.2	+	[aldA]	Repression	Known
1520872	1521293	5.53	+	[yncE]	Repression	
1520872	1521293	5.53	-	[yncD]	Repression	
1608517	1608984	9.41	-	[uxaB]	N/D	
1675706	1676221	7.42	-	[pntA, pntB]	Activation	
1679542	1680354	9	-	[ydgC]	Repression	
1679542	1680354	9	+	[rstA, rstB]	Activation	
1686396	1686946	3.93	-	[fumA]	Repression	Known
1686396	1686946	3.93	+	[manA]	N/D	
1829936	1830386	10.65	-	[astA, astB, astC, astD, astE]	Repression	
1829936	1830386	10.65	+	[xthA]	Repression	
1860590	1861003	9.56	+	[gapA, yeaD]	Repression	
1860590	1861003	9.56	-	[msrB]	Repression	
1887394	1888236	11.08	-	[fadD]	Repression	Known
1976263	1976504	6.64	-	[flhC, flhD]	Activation	
1977375	1977654	8.13	+	[uspC]	N/D	
1977375	1977654	8.13	-	[insA-5, insB-5]	N/D	
2238472	2238897	6.42	-	[mglA, mglB, mglC]	Repression	
2241347	2241935	5.71	+	[yeiG]	N/D	
2241347	2241935	5.71	-	[folE, yeiB]	Repression	
2244605	2245268	8.28	-	[cirA]	Repression	
2263985	2264339	5.96	+	[yeiQ]	Repression	
2276039	2276443	10.69	-	[yejG]	Repression	
2280151	2280914	8.61	+	[rplY]	Activation	
2284173	2284656	5.86	+	[proL]	N/D	
2304509	2304868	4.77	-	[mqo]	Repression	
2310743	2311405	16.34	+	[micF]	Activation	
2310743	2311405	16.34	-	[ompC]	Activation	
2384706	2385289	9.64	+	[yfbM]	N/D	
2402959	2403487	14.09	-	[nuoA, nuoB, nuoC, nuoE, nuoF, nuoG, nuoH, nuoI, nuoJ, nuoK, nuoL, nuoM, nuoN]	Repression	Known
2425948	2426273	4.79	-	[argT, hisJ, hisM, hisP, hisQ]	Repression	
2458400	2458764	5.48	-	[fadI, fadJ]	Repression	Known
2496347	2496888	12.8	+	[ypdA, ypdB, ypdC]	Activation	
2496347	2496888	12.8	-	[yfdZ]	N/D	
2510813	2511330	7.2	-	[mntH]	Repression	
2510813	2511330	7.2	+	[nupC]	Activation	
2561187	2561595	4.1	+	[yffQ, yffR]	N/D	
2631812	2632525	13.65	+	[xseA]	Activation	
2631812	2632525	13.65	-	[guaA, guaB]	N/D	
2642597	2643118	10.23	-	[ndk]	Repression	
2661207	2661749	6.87	+	[suhB]	N/D	
2661207	2661749	6.87	-	[trmJ]	N/D	
2753425	2754054	12.09	+	[ssrA]	N/D	
2786581	2787102	9.1	+	[csiD, gabD, gabP, gabT, lhgO]	Repression	
2922199	2922704	9.25	-	[csrB, truC, yqcA, yqcC]	Repression	
2940499	2940974	8.93	-	[gcvA]	N/D	
2940499	2940974	8.93	+	[gcvB]	Activation	
3125852	3126560	9.5	+	[glcC]	Repression	

3125852	3126560	9.5	-	[glcA, glcB, glcD, glcE, glcF, glcG]	Repression	Known
3144302	3144752	3.03	-	[hybA, hybB, hybC, hybD, hybE, hybF, hybG, hybO, yghW]	Activation	
3208503	3209120	9.59	-	[ygjD]	N/D	
3208503	3209120	9.59	+	[dnaG, rpoD, rpsU]	Activation	
3216816	3217970	56.79	+	[ygjG]	N/D	
3216816	3217970	56.79	-	[aer]	Repression	
3229492	3229925	11.27	+	[fadH]	Repression	Known
3237429	3238167	12.24	+	[sstT]	Activation	
3242529	3242792	4.06	+	[exuT]	N/D	
3242529	3242792	4.06	-	[uxaA, uxaC]	Repression	
3306347	3306826	8.51	-	[nlpI]	Activation	
3309193	3309930	9.77	-	[pnp, rpsO]	N/D	
3309193	3309930	9.77	+	[psrO]	N/D	
3316172	3316576	8.7	+	[argG]	N/D	
3316172	3316576	8.7	-	[infB, metY, nusA, pnp, rbfA, rimP, rpsO, truB]	Activation	
3320118	3320918	18.9	-	[leuU, secG]	Activation	
3331135	3331514	7.54	-	[rplU, rpmA]	Activation	
3351922	3352747	14.07	-	[yhcC]	N/D	
3351922	3352747	14.07	+	[gltB, gltD, gltF]	N/D	
3382354	3383246	10.32	+	[argR]	Activation	
3382354	3383246	10.32	-	[mdh]	Repression	Known
3524059	3524713	7.17	-	[nudE]	Repression	
3524059	3524713	7.17	+	[yrfF]	Activation	
3530384	3530763	3.76	+	[pck]	N/D	
3530384	3530763	3.76	-	[yhgE]	Activation	
3537768	3538389	6.28	+	[feoA, feoB, feoC]	Repression	
3559725	3560000	3.37	+	[glpD]	Repression	Known
3559725	3560000	3.37	-	[glpE, glpG, glpR]	conflict	
3590129	3590604	9.42	-	[ugpA, ugpB, ugpC, ugpE, ugpQ]	Repression	
3628633	3629217	6.86	-	[rbbA, yhhJ, yhiI]	Activation	
3663691	3664091	6.23	-	[gadW, gadX]	Activation	Known
3671224	3673895	203.32	+	[yhjE]	Repression	
3705383	3706208	11.88	-	[dppA, dppB, dppC, dppD, dppF]	Repression	
3710141	3710558	8.83	-	[yhjX]	N/D	
3717470	3718158	12.02	+	[cspA, yiaG]	Repression	
3774832	3775395	11.22	+	[lldD, lldP, lldR]	Repression	Known
3809457	3810128	10.37	-	[mutM, rpmB, rpmG]	Activation	
3882060	3882705	13.25	+	[rnpA, rpmH]	Activation	
3882060	3882705	13.25	-	[dnaA, dnaN, recF]	N/D	
3891506	3892056	5.19	+	[yieE, yieF]	Repression	
3963762	3964341	11.45	-	[rhlB]	Repression	
3963762	3964341	11.45	+	[rho, rhoL, trxA]	Activation	
4028951	4029389	6.33	+	[hemG, pepQ, trkH, yigZ]	Repression	
4028951	4029389	6.33	-	[fadA, fadB]	Repression	Known
4158474	4159090	10.7	+	[fabR, yijD]	Activation	
4158474	4159090	10.7	-	[sthA]	Repression	
4194017	4194596	9.12	-	[thiC, thiE, thiF, thiG, thiH, thiS]	Repression	
4285290	4285644	8.49	+	[nrfA, nrfB, nrfC, nrfD, nrfE, nrfF, nrfG]	Activation	
4285290	4285644	8.49	-	[acs, actP, yjcH]	Repression	
4346724	4347078	5.33	-	[dcuB, fumB]	N/D	
4364658	4365071	12.53	-	[dcuA]	Activation	
4427296	4427950	26.83	+	[cycA]	Repression	
4569507	4569903	12.53	+	[yjiS]	N/D	
4569507	4569903	12.53	-	[yjiR]	Repression	
4637994	4639032	23.97	+	[yjjY]	N/D	
4637994	4639032	23.97	-	[arcA]	Activation	
