# Supplementary material for: Determining the Control Circuitry of Redox Metabolism at the Genome-Scale
Source: PLoS Genet. 2014 Apr 3;10(4):e1004264. doi: 10.1371/journal.pgen.1004264 (PMC3974632; doi:10.1371/journal.pgen.1004264)
Supplement: Table S3 — Fnr-associated regions under fermentative conditions identified by ChIP-chip analysis and its regulatory effect on the target operons determined by expression profiles. This table summarizes the results of ChIP-chip experiments to determine the genome-wide locations of DNA targets for Fnr binding in exponential phase E. coli cells growing in strictly anaerobic minimal media conditions. First and second columns indicate identified Fnr-binding peaks (Start: left-end peak position, End: right-end peak position). The third column indicates the log2 ratio of each Fnr-binding peak. (PDF) [file pgen.1004264.s011.pdf]

Supplementary Table 3. Fnr-associated regions under fermentative conditions identified by ChIP-chip analysis and its regulatory effect on the target operons determined by expression profiles. This table summarizes the results of ChIP-chip experiments to determine the genome-wide locations of DNA targets for Fnr binding in exponential phase E. coli cells growing in strictly anaerobic minimal media conditions. First and second columns indicate identified Fnr-binding peaks (Start: left-end peak position, End: right-end peak position). The third column indicates the log2 ratio of each Fnr-binding peak.

Peak start	Peak end	Occupancy	Strand	Operon	Regulation	Regulon
22	426	14.1	+	[thrA, thrB, thrC, thrL]	Activation	
33664	34016	4.36	+	[caiF]	Activation	Known
121581	122493	37.33	+	[aceE, aceF, lpd, pdhR]	Repression	Known
121581	122493	37.33	-	[aroP]	Activation	
222754	222983	2.31	-	[metI, metN, metQ]	Activation	
222754	222983	2.31	+	[gmhB]	N/D	
400018	400376	5.4	+	[iraP]	Repression	
400018	400376	5.4	-	[ddlA]	Repression	
579629	579977	5.72	+	[nohB]	N/D	
770097	770680	21.85	+	[cydA, cydB]	Repression	Known
815945	816229	6.18	-	[ybhK]	N/D	
815945	816229	6.18	+	[moaA, moaB, moaC, moaD, moaE]	Activation	Known
854877	855223	5.81	+	[ybiT]	N/D	
854877	855223	5.81	-	[ybiS]	Repression	
862561	863006	4.06	-	[ybiW, ybiY]	N/D	
862561	863006	4.06	+	[fsaA]	N/D	
877230	877764	18.86	+	[bssR]	Activation	
877230	877764	18.86	-	[rimO]	N/D	
915184	915551	4.9	-	[aqpZ]	N/D	
915184	915551	4.9	+	[ybjD]	N/D	
929843	930127	2.35	-	[cydC, cydD]	Repression	Known
939760	940277	8.66	+	[dmsA, dmsB, dmsC]	Activation	Known
953406	954037	25.12	-	[focA, pflB]	Activation	Known
1003698	1004086	11.66	+	[pyrD]	Activation	
1156969	1157306	4.58	+	[ptsG]	Activation	
1249958	1250388	8.05	-	[dhaK, dhaL, dhaM]	Activation	
1249958	1250388	8.05	+	[dhaR]	N/D	
1276714	1276968	2.47	+	[narK]	N/D	Known
1276714	1276968	2.47	-	[narL, narX]	Activation	Known
1278930	1279184	3.95	+	[narG, narH, narI, narJ]	Activation	Known
1297064	1298014	43.41	-	[adhE]	Activation	Known
1297064	1298014	43.41	+	[ychE]	N/D	
1311573	1312065	9.82	+	[ompW]	N/D	Known
1311573	1312065	9.82	-	[yciB, yciC]	N/D	
1397425	1397801	5.97	-	[fnr]	Activation	Known
1406606	1407751	57.16	+	[dbpA, fnrS]	N/D	
1433809	1434164	4.34	-	[uspF]	Activation	
1514693	1515851	55.69	+	[ydcX, ydcY]	N/D	
1514693	1515851	55.69	-	[yncL]	N/D	
1545268	1545543	6.22	+	[fdnG, fdnH, fdnI]	N/D	Known
1545268	1545543	6.22	-	[yddG]	N/D	
1608609	1608992	7.06	-	[uxaB]	N/D	
1627076	1627334	7.04	+	[ydfZ]	Activation	
1634523	1634804	5.29	-	[nohA, tfaQ, ydfN]	N/D	
1634523	1634804	5.29	+	[ydfO, ynfO]	N/D	
1655846	1656200	3.67	+	[dmsD, ynfE, ynfF, ynfG, ynfH]	Activation	Known
1664954	1665609	25.27	-	[ynfK]	Activation	
1717589	1717980	5.18	-	[anmK]	N/D	
1717589	1717980	5.18	+	[slyB]	N/D	
1718564	1719676	55.45	-	[slyA]	N/D	

1718564	1719676	55.45	+	[ydhI, ydhJ, ydhK]	N/D	
1752229	1752712	5.61	-	[ydhT, ydhU, ydhV, ydhW, ydhX, ydhY]	Activation	Known
1830936	1831736	8.95	+	[ydjX, ydjY, ydjZ, ynjA, ynjB, ynjC, ynjD]	N/D	
1837249	1837857	7.7	+	[ynjE]	Activation	
1860528	1860849	7.43	-	[msrB]	Repression	
1860528	1860849	7.43	+	[gapA, yeaD]	Activation	
1868390	1868661	5.34	+	[yeaI]	N/D	
1935038	1936071	49.24	+	[pykA]	Activation	
1987416	1987641	5.43	-	[yecH]	Activation	
1987416	1987641	5.43	+	[tyrP]	N/D	
2006399	2006691	2.74	+	[yedE, yedF]	Repression	
2263835	2264310	8.31	+	[yeiQ]	N/D	
2342209	2342718	9.16	-	[yfaL]	N/D	
2342209	2342718	9.16	+	[nrdA, nrdB, yfaE]	Repression	
2363905	2364372	5.67	+	[arnA, arnB, arnC, arnD, arnE, arnF, arnT]	Repression	
2403298	2403613	8.35	-	[nuoA, nuoB, nuoC, nuoE, nuoF, nuoG, nuoH, nuoI, nuoJ, nuoK, nuoL, nuoM, nuoN]	Activation	Known
2411077	2411786	29.13	+	[ackA, pta]	Activation	Known
2411077	2411786	29.13	-	[yfbV]	Activation	
2414873	2415198	7.73	+	[yfcC]	N/D	
2458993	2459343	6.04	-	[yfcZ]	Activation	
2458993	2459343	6.04	+	[fadL]	N/D	
2558286	2558519	4	+	[yffL]	N/D	
2561320	2561654	5.81	+	[yffQ, yffR]	N/D	
2562241	2562791	18.73	+	[yffS]	Repression	
2618825	2619146	6.78	+	[purM, purN]	Activation	Known
2618825	2619146	6.78	-	[upp, uraA]	Activation	Known
2632029	2632437	6.67	-	[guaA, guaB]	Activation	
2632029	2632437	6.67	+	[xseA]	N/D	
2713973	2715190	58.62	+	[ung]	Activation	
2713973	2715190	58.62	-	[yfiD]	Activation	Known
2890562	2890817	5.1	+	[ygcN, ygcO, ygcP]	N/D	
2945028	2945524	14.13	+	[metV, metW, metZ]	Activation	
2945028	2945524	14.13	-	[mltA]	Activation	
3144260	3144589	11.19	-	[hybA, hybB, hybC, hybD, hybE, hybF, hybG, hybO, yghW]	Activation	
3151464	3151697	4.26	+	[yghB]	Activation	
3242629	3243193	19.34	-	[uxaA, uxaC]	N/D	Known
3242629	3243193	19.34	+	[exuT]	N/D	
3265477	3265748	3.64	+	[tdcR, yhaB, yhaC]	N/D	
3273154	3273500	5.94	-	[garK, garL, garP, garR, rnpB]	N/D	Known
3273154	3273500	5.94	+	[garD]	N/D	
3299318	3299760	7.37	+	[yhbU, yhbV]	Activation	
3299318	3299760	7.37	-	[yhbS, yhbT]	Activation	
3351968	3352343	6.32	+	[gltB, gltD, gltF]	N/D	Known
3351968	3352343	6.32	-	[yhcC]	N/D	
3491858	3492242	5.75	+	[cysG, nirB, nirC, nirD]	Activation	Known
3572961	3573386	7.17	-	[asd]	N/D	
3572961	3573386	7.17	+	[yhgN]	N/D	
3611421	3611733	6.6	+	[nikA, nikB, nikC, nikD, nikE, nikR]	Activation	Known
3635513	3635921	4.74	+	[pitA]	Activation	Known
3635513	3635921	4.74	-	[yhiN]	N/D	
3637554	3638025	5.57	+	[uspA]	N/D	
3637554	3638025	5.57	-	[uspB]	Repression	
3782782	3783328	6.56	-	[yibN]	Activation	
3782782	3783328	6.56	+	[envC, gpmM, yibQ]	Activation	

3929133	3929421	3.21	-	[ravA, viaA]	Activation	
3929133	3929421	3.21	+	[kup]	N/D	
4131553	4131899	4.29	+	[katG]	Activation	Known
4285636	4285952	3.78	-	[acs, actP, yjcH]	N/D	
4285636	4285952	3.78	+	[nrfA, nrfB, nrfC, nrfD, nrfE, nrfF, nrfG]	Activation	Known
4368267	4368629	4.39	+	[groL, groS]	Repression	
4368267	4368629	4.39	-	[yjeH]	N/D	
4380104	4380617	6.2	-	[frdA, frdB, frdC, frdD]	Activation	Known
4380104	4380617	6.2	+	[poxA]	N/D	
4402547	4402859	5.1	+	[purA, yjeT]	Activation	
4460936	4461436	5.15	-	[nrdD, nrdG]	Activation	Known
4638482	4638719	3.06	-	[arcA]	N/D	Known
4638482	4638719	3.06	+	[yjjY]	N/D	
