# Supplementary material for: Determining the Control Circuitry of Redox Metabolism at the Genome-Scale
Source: PLoS Genet. 2014 Apr 3;10(4):e1004264. doi: 10.1371/journal.pgen.1004264 (PMC3974632; doi:10.1371/journal.pgen.1004264)
Supplement: Table S4 — Fnr-associated regions under nitrate respiratory conditions identified by ChIP-chip analysis and its regulatory effect on the target operons determined by expression profiles. This table summarizes the results of ChIP-chip experiments to determine the genome-wide locations of DNA targets for Fnr binding in exponential phase E. coli cells growing in strictly anaerobic minimal media with the addition of 20 mm KNO3. First and second columns indicate identified Fnr-binding peaks (Start: left-end peak position, End: right-end peak position). The third column indicates the log2 ratio of each Fnr-binding peak. (PDF) [file pgen.1004264.s012.pdf]

Supplementary Table 4. Fnr-associated regions under nitrate respiratory conditions identified by ChIP-chip analysis and its regulatory effect on the target operons determined by expression profiles. This table summarizes the results of ChIP-chip experiments to determine the genome-wide locations of DNA targets for Fnr binding in exponential phase E. coli cells growing in strictly anaerobic minimal media with the addition of 20mm KNO3. First and second columns indicate identified Fnr-binding peaks (Start: left-end peak position, End: right-end peak position). The third column indicates the log2 ratio of each Fnr-binding peak.

Peak start	Peak end	Occupancy	Strand	Operon	Regulation	Regulon DB
26	226	4.97	+	[thrA, thrB, thrC, thrL]	Activation	
121564	122410	30.6	-	[aroP]	Activation	
121564	122410	30.6	+	[aceE, aceF, lpd, pdhR]	N/D	Known
236760	237227	5.33	+	[aspV, yafT]	N/D	
579604	579842	3.82	+	[nohB]	N/D	
695756	696406	9.89	-	[glnU, glnV, glnW, glnX, leuW, metT, metU]	N/D	
770355	770567	5.48	+	[cydA, cydB]	Repression	Known
815320	816641	59.73	-	[ybhK]	N/D	
815320	816641	59.73	+	[moaA, moaB, moaC, moaD, moaE]	Activation	Known
855031	855452	5.87	-	[ybiS]	Repression	
855031	855452	5.87	+	[ybiT]	Activation	
862543	862976	4.08	-	[ybiW, ybiY]	N/D	
862543	862976	4.08	+	[fsaA]	N/D	
876814	878039	50.27	+	[bssR]	Activation	
876814	878039	50.27	-	[rimO]	Activation	
913001	913501	7.74	-	[hcp, hcr]	Activation	Known
915063	915834	8.71	-	[aqpZ]	N/D	
915063	915834	8.71	+	[ybjD]	N/D	
953498	953789	7.98	-	[focA, pflB]	N/D	Known
1003869	1004148	5.92	+	[pyrD]	Activation	
1156535	1157177	7.77	+	[ptsG]	Activation	
1184805	1185388	4.58	+	[pepT]	Activation	Known
1184805	1185388	4.58	-	[potA, potB, potC, potD]	Activation	
1276906	1277366	6.31	+	[narK]	Activation	Known
1276906	1277366	6.31	-	[narL, narX]	Activation	Known
1278180	1279764	73.67	+	[narG, narH, narI, narJ]	Activation	Known
1296968	1298072	47.48	+	[ychE]	N/D	
1296968	1298072	47.48	-	[adhE]	Repression	Known
1311760	1312152	6.02	-	[yciB, yciC]	N/D	
1311760	1312152	6.02	+	[ompW]	N/D	Known
1397471	1397939	8.53	-	[fnr]	Activation	Known
1406401	1407831	63.7	+	[fnrS]	N/D	
1514839	1515743	32.76	+	[ydcX, ydcY]	Activation	
1514839	1515743	32.76	-	[yncL]	N/D	
1544314	1546189	106.67	-	[yddG]	N/D	
1544314	1546189	106.67	+	[fdnG, fdnH, fdnI]	Activation	Known
1608517	1608938	6.67	-	[uxaB]	N/D	
1627005	1627617	18.59	+	[ydfZ]	Activation	
1634571	1634863	5.26	+	[ydfO, ynfO]	Activation	
1634571	1634863	5.26	-	[nohA, tfaQ, ydfN]	N/D	
1664692	1665946	53.84	-	[ynfK]	Activation	
1718130	1720026	100.59	-	[slyA]	N/D	
1718130	1720026	100.59	+	[ydhI, ydhJ, ydhK]	Activation	
1831161	1831557	5.56	+	[ydjX, ydjY, ydjZ, ynjA, ynjB, ynjC, ynjD]	Activation	
1837153	1837632	4.05	+	[ynjE]	Activation	
1860499	1860832	8.55	+	[gapA, yeaD]	Activation	
1860499	1860832	8.55	-	[msrB]	N/D	
1934600	1936308	88.12	+	[pykA]	Activation	

1987357	1987603	6.06	+	[tyrP]	Repression	
1987357	1987603	6.06	-	[yecH]	Activation	
2066085	2066718	10.16	+	[yoeA]	N/D	
2263926	2264272	4.83	+	[yeiQ]	N/D	
2403344	2403602	6.48	-	[nuoA, nuoB, nuoC, nuoE, nuoF, nuoG, nuoH, nuoI, nuoJ, nuoK, nuoL, nuoM, nuoN]	Activation	Known
2410977	2412073	46.04	-	[yfbV]	N/D	
2410977	2412073	46.04	+	[ackA, pta]	Activation	Known
2414940	2415461	6.67	+	[yfcC]	Activation	
2458905	2459376	4.29	+	[fadL]	N/D	
2458905	2459376	4.29	-	[yfcZ]	Activation	
2558440	2558665	5.59	+	[yffL]	Repression	
2562237	2562725	11.83	+	[yffS]	Activation	
2618942	2619273	3.23	+	[purM, purN]	Activation	Known
2618942	2619273	3.23	-	[upp, uraA]	Activation	Known
2632000	2632387	8.93	-	[guaA, guaB]	Activation	
2632000	2632387	8.93	+	[xseA]	Activation	
2713953	2715153	48.39	-	[yfiD]	Activation	Known
2713953	2715153	48.39	+	[ung]	N/D	
2890525	2890817	4.66	+	[ygcN, ygcO, ygcP]	N/D	
2907727	2908047	4.22	-	[eno, pyrG]	Repression	
2922168	2922822	8.51	-	[csrB, truC, yqcA, yqcC]	Repression	
2945182	2945724	16.82	-	[mltA]	N/D	
2945182	2945724	16.82	+	[metV, metW, metZ]	N/D	
3084255	3084560	4.53	+	[metK, yqgC]	Activation	
3084255	3084560	4.53	-	[speA, speB, yqgB, yqgD]	N/D	
3144135	3144760	21.44	-	[hybA, hybB, hybC, hybD, hybE, hybF, hybG, hybO, yghW]	Activation	
3147210	3147781	3.95	+	[yghA]	Repression	
3147210	3147781	3.95	-	[yqhA]	N/D	
3151364	3151772	9.42	+	[yghB]	N/D	
3242534	3242884	9.62	-	[uxaA, uxaC]	Activation	Known
3242534	3242884	9.62	+	[exuT]	N/D	
3299330	3299672	6.43	+	[yhbU, yhbV]	Activation	
3299330	3299672	6.43	-	[yhbS, yhbT]	Activation	
3316235	3316635	6.15	-	[infB, metY, nusA, pnp, rbfA, rimP, rpsO, truB]	Activation	
3316235	3316635	6.15	+	[argG]	N/D	
3320151	3320668	6.24	-	[leuU, secG]	Activation	
3331189	3331701	6.94	-	[rplU, rpmA]	N/D	
3331189	3331701	6.94	+	[ispB]	N/D	
3352038	3352480	7.62	+	[gltB, gltD, gltF]	N/D	Known
3352038	3352480	7.62	-	[yhcC]	N/D	
3491479	3492542	40.58	+	[cysG, nirB, nirC, nirD]	Activation	Known
3611367	3611767	3.6	+	[nikA, nikB, nikC, nikD, nikE, nikR]	Activation	Known
3635392	3635729	7.54	-	[yhiN]	N/D	
3635392	3635729	7.54	+	[pitA]	Activation	Known
3637692	3638108	5.71	-	[uspB]	Repression	
3637692	3638108	5.71	+	[uspA]	Repression	
3882047	3882601	7.49	-	[dnaA, dnaN, recF]	Activation	
3882047	3882601	7.49	+	[rnpA, rpmH]	Activation	
3929016	3929641	8.36	-	[ravA, viaA]	Activation	
3929016	3929641	8.36	+	[kup]	N/D	
4131332	4131662	6.68	+	[katG]	Activation	Known
4285448	4285982	4.94	+	[nrfA, nrfB, nrfC, nrfD, nrfE, nrfF, nrfG]	Activation	Known
4285448	4285982	4.94	-	[acs, actP, yjcH]	N/D	
4402434	4402838	7.42	+	[purA, yjeT]	Activation	
4584625	4584929	3.65	-	[hsdM, hsdR, hsdS]	Activation	

4584625	4584929	3.65	+	[mrr]	N/D	
4603825	4604367	9.28	+	[yjjZ]	N/D	
4603825	4604367	9.28	-	[fhuF, leuP, leuQ, leuV]	Repression	
4638344	4639019	11.39	+	[yjjY]	N/D	
4638344	4639019	11.39	-	[arcA]	Activation	Known
