# Supplementary material for: Determining the Control Circuitry of Redox Metabolism at the Genome-Scale
Source: PLoS Genet. 2014 Apr 3;10(4):e1004264. doi: 10.1371/journal.pgen.1004264 (PMC3974632; doi:10.1371/journal.pgen.1004264)
Supplement: Table S5 — ArcA motifs found underneath experimentally determined ChIP binding regions. First column is the center position of the peak (averaged for peaks occuring under both fermentative and nitrate respiratory conditions). (PDF) [file pgen.1004264.s013.pdf]

Supplementary Table 5. ArcA motifs found underneath experimentally determined ChIP binding regions. First column is the center position of the peak (averaged for peaks occuring under both fermentative and nitrate respiratory conditions).

Peak center	Operon	Motif start	Motif end	Sequence	P-val	Strand
150	[thrA, thrB, thrC, thrL]	99	113	ATTAAAATTTTATTG	6.28E-05	-
29420	[carA, carB]	29341	29355	GTTGTTTTAATGTAA	9.60E-06	-
41890	[caiA, caiB, caiC, caiD, caiE, caiT]	41979	41993	GTTTTATTTTTGTTA	8.94E-06	-
41890	[caiA, caiB, caiC, caiD, caiE, caiT]	41992	42006	TAACATTTAATATAA	2.72E-05	+
121924	[aceE, aceF, aroP, lpd, pdhR]	121624	121638	TATTAATTTGTTTAC	1.63E-05	+
121924	[aceE, aceF, aroP, lpd, pdhR]	121870	121884	GTTTAATAATCGTTA	1.42E-06	-
121924	[aceE, aceF, aroP, lpd, pdhR]	121924	121938	GTTACAACTTTGAAA	7.43E-05	-
121924	[aceE, aceF, aroP, lpd, pdhR]	121925	121939	TTACAACTTTGAAAC	5.12E-05	+
121924	[aceE, aceF, aroP, lpd, pdhR]	122009	122023	GTGAATACTTTGTTA	7.22E-06	-
127841	[lpd]	127681	127695	GTTTAAAAATTGTTA	1.52E-07	-
127841	[lpd]	127683	127697	TTAAAAATTGTTAAC	2.22E-05	+
127841	[lpd]	127694	127708	TAACAATTTTGTAAA	2.81E-06	+
131448	[acnB, yacH]	131399	131413	TAACGAAGTTTTTAC	8.40E-06	+
131448	[acnB, yacH]	131464	131478	GTTGCTTTTTTGTAA	3.07E-05	-
243379	[fadE, lpcA]	243376	243390	TAGCAATATGTTTAC	1.31E-05	+
243379	[fadE, lpcA]	243387	243401	TTACAATATAATTAC	5.35E-06	+
450990	[cyoA, cyoB, cyoC, cyoD, cyoE]	450897	450911	GTTACATTACTGTAA	3.87E-05	-
450990	[cyoA, cyoB, cyoC, cyoD, cyoE]	451070	451084	AAACAATTATTTAAC	2.63E-07	+
450990	[cyoA, cyoB, cyoC, cyoD, cyoE]	451079	451093	TTTAACAAATAATTA	7.46E-06	-
450990	[cyoA, cyoB, cyoC, cyoD, cyoE]	451081	451095	TAACAAATAATTATC	9.56E-05	+
579763	[nohB]	579712	579726	GTTGTTTTTACGTTA	2.00E-05	-
728180	[kdpA, kdpB, kdpC, kdpF, ybfA]	728128	728142	GTAAAAAAAGTATAA	8.15E-05	-
728180	[kdpA, kdpB, kdpC, kdpF, ybfA]	728161	728175	TTTAACTAATGTTTA	8.88E-05	-
728180	[kdpA, kdpB, kdpC, kdpF, ybfA]	728185	728199	TAACTTTTGTGTAAC	1.44E-05	+
754402	[sdhA, sdhB, sdhC, sdhD, sucA, sucB, sucC, sucD]	753963	753977	GTTAATCTTAGGTGA	9.10E-05	-
754307	[gltA, sdhA, sdhB, sdhC, sdhD, sucA, sucB, sucC, sucD]	754029	754043	GTAACAACTTTGTTG	3.97E-05	-
754307	[gltA, sdhA, sdhB, sdhC, sdhD, sucA, sucB, sucC, sucD]	754332	754346	TAACAGAAAGTTAAC	9.43E-06	+
770285	[cydA, cydB]	770047	770061	TAACAAAATAATAAC	5.44E-07	+
770285	[cydA, cydB]	770084	770098	TAATTATAAGTTAAC	1.46E-05	+
770285	[cydA, cydB]	770093	770107	GTTAACTAAATGTTA	1.34E-08	-
770285	[cydA, cydB]	770095	770109	TAACTAAATGTTAAT	8.40E-06	+
770355	[cydA, cydB]	770264	770278	TCACACATTTTAAAC	1.03E-05	+
770355	[cydA, cydB]	770275	770289	AAACATAAATGTCAC	8.23E-05	+
770355	[cydA, cydB]	770294	770308	GTTACCTTATTGAAA	6.03E-05	-
770355	[cydA, cydB]	770295	770309	TTACCTTATTGAAAC	5.41E-05	+
770355	[cydA, cydB]	770496	770510	GTAATATATACGTGG	7.43E-05	-
770355	[cydA, cydB]	770525	770539	TTTATTAACATGTTT	9.56E-05	-
770355	[cydA, cydB]	770530	770544	TAACATGTTTGCAAC	1.54E-05	+
858444	[ybiU]	858342	858356	GTTATAAATAAGTTA	7.38E-07	-
892706	[potF, potG, potH, potI]	892722	892736	TAACCTTTTCTTAAA	4.59E-05	+
892706	[potF, potG, potH, potI]	892792	892806	TAAAAATTTGTTAAA	7.46E-06	+
930386	[cydC, cydD]	930291	930305	GTTACTGATTTGTAA	2.60E-06	-
953676	[focA, pflB]	953817	953831	GTAAATATTTGATTA	1.63E-05	-
953676	[focA, pflB]	953895	953909	AAACAGTTAATTAAA	7.82E-05	+
953671	[focA, pflB]	953964	953978	TTACCGATATTTAAC	3.07E-05	+
953671	[focA, pflB]	953975	953989	TAACCTTATAATTAC	9.60E-06	+

953671	[focA, pflB]	953986	954000	TTACAATTATTTTAT	3.29E-05	+
989814	[pepN, pncB]	989724	989738	GTTATAAAAATGTAG	1.30E-06	-
1015227	[rmf]	1015286	1015300	GTTAACAATGCGTTT	1.03E-05	-
1015227	[rmf]	1015288	1015302	TAACAATGCGTTTAA	1.62E-06	+
1073279	[rutA, rutB, rutC, rutD, rutE, rutF, rutG, rutR]	1073279	1073293	GTTTTTAAAAAGTTA	2.21E-06	-
1073279	[rutA, rutB, rutC, rutD, rutE, rutF, rutG, rutR]	1073296	1073310	GTTATTAATCTGTTA	2.21E-06	-
1073279	[rutA, rutB, rutC, rutD, rutE, rutF, rutG, rutR]	1073298	1073312	TATTAATCTGTTAAC	1.63E-05	+
1073279	[rutA, rutB, rutC, rutD, rutE, rutF, rutG, rutR]	1073307	1073321	GTTAACATTACGTTA	1.56E-06	-
1078409	[putA, putP]	1078378	1078392	GTAAATGGTGTGTTA	5.26E-05	-
1084185	[phoH]	1084107	1084121	GTTAAATATAAGTAA	1.42E-06	-
1084185	[phoH]	1084118	1084132	GTAAATATATTGTTG	4.24E-06	-
1150977	[acpP, fabF]	1150777	1150791	CAACATTTTATACAC	1.26E-05	+
1194219	[icd, nudJ, rluE]	1194236	1194250	TTACAAATCATTAAC	4.86E-07	+
1232411	[dsbB]	1232395	1232409	GTTATCAAAGCGTTG	1.26E-05	-
1276988	[narK, narL, narX]	1277094	1277108	GTTATCTTATCGTTT	2.41E-05	-
1276988	[narK, narL, narX]	1277101	1277115	TATCGTTTGATTTAC	7.82E-05	+
1279014	[narG, narH, narI, narJ]	1278696	1278710	GTAAAATATTTGATT	4.34E-05	-
1297536	[adhE, ychE]	1297635	1297649	GTTAATAAATTGTAG	4.04E-07	-
1298745	[oppA, oppB, oppC, oppD, oppF]	1298726	1298740	TAATCAATTGTTAAA	4.11E-06	+
1298745	[oppA, oppB, oppC, oppD, oppF]	1298883	1298897	TAACAATTTTGCAAA	3.45E-05	+
1311888	[ompW, yciB, yciC]	1312011	1312025	TCACGTTTTTATAAC	1.71E-06	+
1359092	[puuA, puuD, puuR]	1358963	1358977	GTTATCAAAGCGTAA	6.70E-06	-
1386765	[tpx, ycjG]	1386849	1386863	GTTTACATATAGTTA	1.18E-05	-
1386765	[tpx, ycjG]	1386851	1386865	TTACATATAGTTAAC	2.63E-07	+
1395504	[ynaJ]	1395345	1395359	TTACGTTTAGGTAAC	3.34E-06	+
1397659	[fnr]	1397666	1397680	TATCTATTTGAAAAC	6.70E-05	+
1451846	[maoC, paaA, paaB, paaC, paaD, paaE, paaF, paaG, paaH, paaI, paaJ, paaK]	1451738	1451752	TTTAACGAAATGTTA	5.29E-06	-
1451846	[maoC, paaA, paaB, paaC, paaD, paaE, paaF, paaG, paaH, paaI, paaJ, paaK]	1451740	1451754	TAACGAAATGTTAAC	1.43E-06	+
1451846	[maoC, paaA, paaB, paaC, paaD, paaE, paaF, paaG, paaH, paaI, paaJ, paaK]	1451869	1451883	TAACTATTGTGTAAC	1.87E-05	+
1486199	[aldA]	1486208	1486222	TAACAATGTATTCAC	3.90E-06	+
1486199	[aldA]	1486230	1486244	AAACATATAAATCAC	1.44E-05	+
1515281	[ydcX, ydcY, yncL]	1515738	1515752	ATGAACGAATTATTA	6.86E-05	-
1521104	[yncD, yncE]	1521128	1521142	GTAATGAAATTGTAA	1.26E-05	-
1521104	[yncD, yncE]	1521171	1521185	GTTAAGTAATTGAGA	4.10E-05	-
1545328	[fdnG, fdnH, fdnI, yddG]	1545241	1545255	TAGCATTTTTTTAAC	9.86E-07	+
1561177	[ddpA, ddpB, ddpC, ddpD, ddpF, ddpX]	1561204	1561218	TAACTTTTGATCAAC	2.00E-05	+
1634690	[nohA, tfaQ, ydfN, ydfO, ynfO]	1634722	1634736	TAACGTAAAAACAAC	2.00E-05	+
1656023	[dmsD, ynfE, ynfF, ynfG, ynfH]	1655892	1655906	TAACACATTATCGAC	5.71E-05	+
1656023	[dmsD, ynfE, ynfF, ynfG, ynfH]	1656056	1656070	AAACTTTTATATAAC	4.59E-06	+
1665276	[ynfK]	1665358	1665372	TTAAAAAATGTTAAC	4.36E-05	+
1676106	[pntA, pntB, ydgH]	1676104	1676118	TCGCAAAATATTAAC	4.10E-05	+
1676106	[pntA, pntB, ydgH]	1676227	1676241	TAACATTAAATTAAC	6.01E-08	+
1679945	[rstA, rstB, ydgC]	1679735	1679749	CAGCATATAATCAAC	6.36E-05	+
1679945	[rstA, rstB, ydgC]	1680065	1680079	TAACACTTCCATAAC	1.18E-05	+

1684835	[fumC]	1684675	1684689	TCACTTATTATTTAC	7.22E-06	+
1684835	[fumC]	1684686	1684700	TTACCATTTGATAAC	3.90E-06	+
1686560	[fumA, manA]	1686483	1686497	ACACACTTTTTTAAC	5.77E-06	+
1717784	[anmK, slyB]	1717761	1717775	GTTTATAATTGGTTG	1.54E-05	-
1717784	[anmK, slyB]	1717895	1717909	TTTCAATGATTAAAC	8.66E-05	+
1753532	[pykF, ydhZ]	1753392	1753406	GTTAAATCTTTGATA	1.54E-05	-
1830074	[astA, astB, astC, astD, astE, xthA]	1830020	1830034	GTAAATAATTCGTTA	8.97E-07	-
1830074	[astA, astB, astC, astD, astE, xthA]	1830031	1830045	GTTATTTATATGTTA	6.01E-08	-
1830074	[astA, astB, astC, astD, astE, xthA]	1830042	1830056	GTTAATAATAAGTAA	6.08E-07	-
1860226	[gapA, msrB, yeaC, yeaD]	1860023	1860037	GTTGCTGAATCGTTT	5.71E-05	-
1860226	[gapA, msrB, yeaC, yeaD]	1860030	1860044	AATCGTTTTTTCAAC	6.36E-05	+
1860226	[gapA, msrB, yeaC, yeaD]	1860500	1860514	TAACAAAACATTAAC	8.97E-07	+
1860548	[gapA, msrB, yeaC, yeaD]	1860758	1860772	CAACCTTTTATTCAC	1.44E-05	+
1887810	[fadD]	1887829	1887843	GTTAACATAATATTA	2.16E-06	-
1887810	[fadD]	1887831	1887845	TAACATAATATTAAC	9.20E-08	+
1935504	[pykA]	1935657	1935671	GTCAACGGAGTATTA	8.88E-05	-
2238644	[mglA, mglB, mglC]	2238433	2238447	GTTAATGAAGTGTTA	2.63E-07	-
2238644	[mglA, mglB, mglC]	2238562	2238576	GTTACATACGGGTTA	6.36E-05	-
2238644	[mglA, mglB, mglC]	2238612	2238626	GTTAAGATACTGTGA	8.23E-05	-
2241641	[folE, yeiB, yeiG]	2241830	2241844	GTTAAAACATCGTTA	1.19E-06	-
2261573	[fruA, fruB, fruK, setB]	2259452	2259466	GTTAAAAGGTTGTAA	6.65E-06	-
2261573	[fruA, fruB, fruK, setB]	2260565	2260579	GTAATATCACTGTTA	5.71E-05	-
2264130	[yeiQ]	2264236	2264250	GTTAATTAATCGTTA	6.73E-08	-
2264130	[yeiQ]	2264247	2264261	GTTACTAAGACGTGA	3.66E-05	-
2276234	[yejG]	2276286	2276300	TCACAATTTGTTTAC	1.24E-06	+
2276234	[yejG]	2276309	2276323	TAACACATCATCAAC	6.22E-06	+
2276234	[yejG]	2276498	2276512	TTACATAAAATAAAC	5.77E-06	+
2284414	[proL]	2284397	2284411	GTTTATTTTCTGTTA	3.07E-05	-
2304795	[mqo]	2304837	2304851	TAACACTTAAGTAAC	4.86E-07	+
2311161	[micF, ompC, rcsB, rcsD]	2311266	2311280	TCACAAATAATTAAC	3.58E-07	+
2311161	[micF, ompC, rcsB, rcsD]	2311491	2311505	TAACAGGTCATAAAC	9.10E-05	+
2385014	[yfbM]	2384824	2384838	GTTATAACTTTGTTA	1.56E-06	-
2403302	[nuoA, nuoB, nuoC, nuoE, nuoF, nuoG, nuoH, nuoI, nuoJ, nuoK, nuoL, nuoM, nuoN]	2403244	2403258	TAACCTTTTGTCAAC	3.07E-06	+
2403302	[nuoA, nuoB, nuoC, nuoE, nuoF, nuoG, nuoH, nuoI, nuoJ, nuoK, nuoL, nuoM, nuoN]	2403285	2403299	TTACATAATATCAAC	4.95E-06	+
2403408	[nuoA, nuoB, nuoC, nuoE, nuoF, nuoG, nuoH, nuoI, nuoJ, nuoK, nuoL, nuoM, nuoN]	2403507	2403521	TTACGTATTTTTTAA	6.03E-05	+
2426013	[argT, hisJ, hisM, hisP, hisQ]	2425933	2425947	GTTAATAAGACGTTG	1.18E-05	-
2458642	[fadI, fadJ, yfcZ]	2458543	2458557	TAACCATTTTTTTAC	1.09E-06	+
2496513	[yfdZ, ypdA, ypdB, ypdC]	2496486	2496500	TAACAAGAAAATTAC	3.26E-05	+
2496513	[yfdZ, ypdA, ypdB, ypdC]	2496502	2496516	ACTCATTTTATTAAC	1.44E-05	+
2496513	[yfdZ, ypdA, ypdB, ypdC]	2496524	2496538	TTTCAATAAATTTAC	3.87E-05	+
2511206	[nupC]	2511149	2511163	GTATCCGTTATGTTA	9.56E-05	-
2511206	[nupC]	2511175	2511189	GTTATCGAAGTGTTA	2.21E-06	-
2531506	[crr, ptsH, ptsI]	2531412	2531426	GTTAAAAATGCGTAA	3.07E-06	-
2562498	[yffS]	2562259	2562273	TAACATCCTGGTAAA	3.97E-05	+
2562498	[yffS]	2562405	2562419	CAACAACTAAACAAC	7.43E-05	+
2642874	[ndk]	2642982	2642996	TAACATTTTTTTAAA	8.97E-07	+

2666938	[hcaB, hcaC, hcaD, hcaE, hcaF, hcaR]	2666971	2666985	TCACATATTAGCAAC	8.34E-06	+
2714567	[ung, yfiD]	2714526	2714540	GTAAAACCATTGTTG	2.98E-05	-
2716655	[trxC, yfiF]	2716693	2716707	TAACATATTAGAAAC	9.86E-07	+
2723722	[kgtP]	2723829	2723843	GTAAAGGAAATATTA	6.86E-05	-
2735059	[raiA]	2735075	2735089	GTTGACAAAAAGTGA	1.35E-05	-
2786926	[csiD, gabD, gabP, gabT, lhgO]	2786862	2786876	GTTACTAATTTGTTG	1.30E-06	-
2786926	[csiD, gabD, gabP, gabT, lhgO]	2786884	2786898	TCACAATAAGAAAAC	3.45E-05	+
2940695	[gcvA, gcvB]	2940619	2940633	TAACAATTAGATCAC	3.90E-06	+
2940695	[gcvA, gcvB]	2940639	2940653	GTAACCTATTAGTTT	7.43E-05	-
2945364	[metV, metW, metZ, mltA]	2945356	2945370	GTAAAAAAAAGGTTG	6.70E-06	-
3071826	[epd, fbaA, pgk]	3071213	3071227	TAACAATTTGATGAC	9.60E-06	+
3071826	[epd, fbaA, pgk]	3071971	3071985	TAACCCTACAATAAC	2.89E-05	+
3071826	[epd, fbaA, pgk]	3072247	3072261	TAACAACCAGTTTCC	4.78E-05	+
3071826	[epd, fbaA, pgk]	3072292	3072306	CAACGCATCTTCAAC	9.10E-05	+
3126199	[glcA, glcB, glcC, glcD, glcE, glcF, glcG]	3126190	3126204	GTTAACTCAATGTTA	4.39E-07	-
3126199	[glcA, glcB, glcC, glcD, glcE, glcF, glcG]	3126192	3126206	TAACTCAATGTTAAA	9.68E-05	+
3126199	[glcA, glcB, glcC, glcD, glcE, glcF, glcG]	3126201	3126215	GTTAAATTGATGTAA	1.44E-05	-
3229773	[fadH]	3229657	3229671	TAACAATTATTTTAC	1.69E-07	+
3237723	[sstT]	3237786	3237800	GTCAAAGAAATGTAA	1.76E-05	-
3237723	[sstT]	3237885	3237899	GCAAACACTTTGTTA	3.62E-06	-
3242778	[exuT, uxaA, uxaC]	3242617	3242631	GTTTTTAAAACGATA	2.13E-05	-
3265612	[tdcR, yhaB, yhaC]	3265507	3265521	TATCAATATTTTAAA	6.28E-05	+
3265612	[tdcR, yhaB, yhaC]	3265549	3265563	GAACAATTGATTAAC	6.86E-05	+
3273327	[garD, garK, garL, garP, garR, rnpB]	3273266	3273280	CCTCATTTTAATAAC	2.89E-05	+
3316404	[argG, infB, metY, nusA, pnp, rbfA, rimP, rpsO, truB]	3316420	3316434	CCACATTTTGTCAAC	1.35E-05	+
3382646	[argR, mdh]	3382529	3382543	CATCAATTTGATAAC	1.65E-05	+
3382646	[argR, mdh]	3382540	3382554	TAACAATTAATTTAC	1.05E-07	+
3492028	[cysG, nirB, nirC, nirD]	3491983	3491997	GTTGCTGAATCGTTA	1.35E-05	-
3492028	[cysG, nirB, nirC, nirD]	3492119	3492133	CAACTTTGATATTAC	8.23E-05	+
3524361	[nudE, yrfF]	3524238	3524252	TTGCATGTTATTTAC	9.10E-05	+
3524361	[nudE, yrfF]	3524248	3524262	TTTACATTTATGTAA	8.23E-05	-
3524361	[nudE, yrfF]	3524249	3524263	TTACATTTATGTAAC	4.04E-07	+
3530592	[pck, yhgE]	3530634	3530648	AAACCAAGATTTAAC	3.45E-05	+
3538015	[feoA, feoB, feoC]	3537892	3537906	TATCATTTCATTAAC	1.42E-06	+
3538015	[feoA, feoB, feoC]	3537912	3537926	AAACCTTAATTAAAC	4.59E-05	+
3559887	[glpD, glpE, glpG, glpR]	3559946	3559960	GTTTTTTCAATGTTA	4.59E-06	-
3590371	[ugpA, ugpB, ugpC, ugpE, ugpQ]	3590424	3590438	TTACAGAAAAATAAC	3.45E-05	+
3590371	[ugpA, ugpB, ugpC, ugpE, ugpQ]	3590434	3590448	ATAACTTTTTTGTTA	6.86E-05	-
3590371	[ugpA, ugpB, ugpC, ugpE, ugpQ]	3590435	3590449	TAACTTTTTTGTTAC	3.61E-06	+
3628925	[rbbA, yhhJ, yhiI]	3628932	3628946	TTACGTTTTATCAAC	2.02E-06	+
3663958	[gadW, gadX]	3664061	3664075	CTACATTTAATAAAC	3.90E-06	+
3663958	[gadW, gadX]	3664183	3664197	GTTTTTACAACGTTA	1.10E-05	-
3672576	[yhjE]	3672608	3672622	TAACTTTTATTTAAC	1.52E-07	+
3705901	[dppA, dppB, dppC, dppD, dppF]	3705906	3705920	GTCAACAGAATGTGA	8.15E-05	-
3710288	[yhjX]	3710227	3710241	GTTATTTAATGGTTT	5.77E-06	-
3710288	[yhjX]	3710247	3710261	GTGACTGTTACGTTA	8.66E-05	-
3769994	[mtlA, mtlD, mtlR, yibH, yibI]	3769995	3770009	TCACAAAGATTCAAC	3.45E-05	+
3775146	[lldD, lldP, lldR]	3775289	3775303	TAACATTTAGTTAAC	1.34E-08	+

3783117	[envC, gpmM, yibN, yibQ]	3783274	3783288	GTTGTCGCAATGTTG	9.10E-05	-
3839816	[nepI]	3839829	3839843	TAACGTTTTTGCAAC	3.90E-06	+
3891772	[yieE, yieF]	3891787	3891801	GTAAAAAAGTTGTAA	6.65E-06	-
3964051	[rhlB, rho, rhoL, trxA]	3964173	3964187	TAACTTAGTGTTGAC	7.47E-05	+
4001289	[yigF, yigG]	4001211	4001225	TAACATAAATTTTAC	1.85E-06	+
4002722	[pldA, yigI]	4002741	4002755	GTTAATGAAATGTTG	3.58E-07	-
4002722	[pldA, yigI]	4002855	4002869	ATCAATAATTCGTTA	6.86E-05	-
4029098	[fadA, fadB, hemG, pepQ, trkH, yigZ]	4029047	4029061	GTGTATTTTGTGTTA	7.43E-05	-
4029098	[fadA, fadB, hemG, pepQ, trkH, yigZ]	4029058	4029072	GTTAAAAATATGCAA	6.22E-06	-
4109884	[tpiA, yiiQ]	4109979	4109993	TCTCATTAATTTTAC	6.36E-05	+
4131612	[katG]	4131522	4131536	GTTTATAAATAGTGT	3.45E-05	-
4131612	[katG]	4131562	4131576	TCACAAATTTTAAAC	3.07E-06	+
4131612	[katG]	4131737	4131751	CTACATCTCTTTAAC	3.66E-05	+
4158805	[fabR, sthA, yijD]	4158690	4158704	CAACATTTTGATAAC	6.08E-07	+
4158805	[fabR, sthA, yijD]	4158834	4158848	ATACATAAAAGCAAC	9.10E-05	+
4158805	[fabR, sthA, yijD]	4158845	4158859	CAACAGAATGGTAAC	9.68E-05	+
4194378	[thiC, thiE, thiF, thiG, thiH, thiS]	4194328	4194342	GTAAATTACGCGTTA	3.45E-05	-
4213342	[aceA, aceB, aceK]	4213145	4213159	TAATTTACTGTTTAC	8.88E-05	+
4213342	[aceA, aceB, aceK]	4213345	4213359	GTTATCAACAAGTTA	1.18E-05	-
4285390	[acs, actP, nrfA, nrfB, nrfC, nrfD, nrfE, nrfF, nrfG, yjcH]	4285296	4285310	GTTAATAGATTGTTG	3.62E-06	-
4285390	[acs, actP, nrfA, nrfB, nrfC, nrfD, nrfE, nrfF, nrfG, yjcH]	4285298	4285312	TAATAGATTGTTGAT	8.15E-05	+
4285390	[acs, actP, nrfA, nrfB, nrfC, nrfD, nrfE, nrfF, nrfG, yjcH]	4285417	4285431	GTTAATAATATGTGG	1.42E-06	-
4285754	[acs, actP, nrfA, nrfB, nrfC, nrfD, nrfE, nrfF, nrfG, yjcH]	4285640	4285654	TTACAATTGATTAAA	2.70E-05	+
4323332	[phnC, phnD, phnE, phnF, phnG, phnH, phnI, phnJ, phnK, phnL, phnM, phnN, phnO, phnP]	4323244	4323258	GTTAATTAAAAGTGA	1.09E-06	-
4346914	[dcuB, fumB]	4346757	4346771	GTAAATAACATGTGT	4.59E-05	-
4346914	[dcuB, fumB]	4347010	4347024	GTTAATGTTTTGTTT	6.70E-06	-
4346914	[dcuB, fumB]	4347028	4347042	GTTATCTTATTGATA	1.87E-05	-
4364899	[dcuA]	4364814	4364828	TTTAACAAGTTGATA	2.70E-05	-
4364899	[dcuA]	4364836	4364850	GTTATTTTTAAGTTA	4.59E-06	-
4427622	[cycA]	4427735	4427749	AAACATAAAATTAAC	1.85E-06	+
4453635	[fbp, mpl]	4453621	4453635	TAACGTTTTCATAAA	5.41E-05	+
4560721	[yjiJ]	4560722	4560736	GTTGTTAACAAGTTA	3.45E-05	-
4560721	[yjiJ]	4560725	4560739	GTTAACAAGTTAATA	2.00E-05	-
4560721	[yjiJ]	4560727	4560741	TAACAAGTTAATAAC	1.19E-06	+
4560721	[yjiJ]	4560738	4560752	TAACAAAAAGGTGAA	8.15E-05	+
4569664	[yjiR, yjiS]	4569690	4569704	GTTAAATAAAAGTAA	6.63E-07	-
4609095	[osmY]	4609146	4609160	GTTTCAAAATTGTGA	6.70E-06	-
4609095	[osmY]	4609169	4609183	TAACAAAGTGATGAC	7.82E-05	+
4638669	[arcA, yjjY]	4638636	4638650	GTTAACAGTTTGATG	8.15E-05	-
4638669	[arcA, yjjY]	4638713	4638727	ATACAACTAATAAAC	7.43E-05	+
4638697	[arcA, yjjY]	4638881	4638895	GTTAAGAATTTGTAA	2.02E-06	-
