# Supplementary material for: Determining the Control Circuitry of Redox Metabolism at the Genome-Scale
Source: PLoS Genet. 2014 Apr 3;10(4):e1004264. doi: 10.1371/journal.pgen.1004264 (PMC3974632; doi:10.1371/journal.pgen.1004264)
Supplement: Table S7 — Mean flux values above .1 mmol/GDWH across all sampling points under anaerobic conditions. This table shows all reactions, whether or not they are directly regulated by ArcA or Fnr, their mean flux values, the percent of the total flux that this flux values corresponds too, and the list of genes associated with the reaction. For each reaction the regulation column is TRUE if at least one gene is directly regulated by ArcA or Fnr. The total percent of flux regulated can then be calculated by summing across all flux values which are regulated and dividing by the total. (PDF) [file pgen.1004264.s015.pdf]

Supplementary Table 7. Mean flux values above .1mmol/GDWH across all sampling points under anaerobic conditons. This table shows all reactions, whether or not they are direclty regulated by ArcA or Fnr, their mean flux values, the percent of the total flux that this flux values corresponds too, and the list of genes associated with the reaction. For each reaction the regulation column is TRUE if at least one gene is directly regulated by ArcA or Fnr. The total percent of flux regulated can then be calculated by summing across all flux values which are regulated and dividing by the total.

Reaction ID	Regulation	Flux	Percent total flux	Genes
Htex_reverse	TRUE	27.7808	8.4607%	[u'ompN', u'ompF', u'phoE', u'ompC']
PGK_reverse	FALSE	19.3293	5.8868%	[u'pgk']
GAPD	TRUE	19.3290	5.8867%	[u'gapA']
ENO	FALSE	18.9460	5.7701%	[u'eno']
PGM_reverse	TRUE	18.9443	5.7695%	[u'gpmM', u'ytjC', u'gpmA']
PFL	TRUE	17.2725	5.2604%	[u'pflD', u'pflC', u'pflA', u'pflB', u'tdcE', u'yfiD']
FORtex_reverse	TRUE	17.2493	5.2533%	[u'ompN', u'ompC', u'ompF', u'phoE']
FORtppi	TRUE	17.2453	5.2521%	[u'focA', u'focB']
GLCptspp	TRUE	10.0000	3.0455%	[u'ptsI', u'manZ', u'manY', u'ptsG', u'malX', u'manX', u'crr', u'ptsH']
GLCtexi	FALSE	9.9920	3.0431%	[u'lamB']
PGI	FALSE	9.9561	3.0321%	[u'pgi']
TPI	FALSE	9.7057	2.9559%	[u'tpiA']
ACtex_reverse	TRUE	8.2318	2.5070%	[u'ompN', u'ompC', u'phoE', u'ompF']
ACKr_reverse	TRUE	8.0978	2.4662%	[u'purT', u'ackA', u'tdcD']
PTAr	TRUE	8.0941	2.4651%	[u'pta', u'eutD']
ETOHtex_reverse	TRUE	8.0876	2.4631%	[u'ompN', u'ompF', u'ompC', u'phoE']
ALCD2x_reverse	TRUE	8.0874	2.4630%	[u'adhP', u'adhE', u'frmA']
ACALD_reverse	TRUE	8.0868	2.4629%	[u'adhE', u'mhpF']
F6PA	FALSE	7.8669	2.3959%	[u'fsaB', u'fsaA']
DHAPT	TRUE	7.8646	2.3952%	[u'dhaL', u'dhaK', u'dhaM', u'ptsI', u'ptsH']
ATPS4rpp_reverse	FALSE	6.4443	1.9626%	[u'atpG', u'atpB', u'atpI', u'atpH', u'atpF', u'atpE', u'atpC', u'atpD', u'atpA']
THD2pp	TRUE	2.9009	0.8835%	[u'pntA', u'pntB']
NH4tpp	FALSE	2.5488	0.7762%	[u'amtB', None]
NH4tex	TRUE	2.5481	0.7760%	[u'ompN', u'ompF', u'ompC', u'phoE']
GLUDy_reverse	FALSE	2.0117	0.6127%	[u'gdhA']
H2Otex	TRUE	1.8513	0.5638%	[u'ompN', None, u'ompA', u'ompF', u'phoE', u'ompC', u'ompG', u'ompL']
H2Otpp	FALSE	1.8476	0.5627%	[u'aqpZ', None]
FBA	FALSE	1.7950	0.5467%	[u'fbaB', u'fbaA', u'ydjI']
PFK	FALSE	1.7927	0.5460%	[u'pfkA', u'pfkB']
PPKr_reverse	FALSE	0.8995	0.2740%	[u'ppk']
PPC	FALSE	0.7866	0.2395%	[u'ppc']
ASPTA_reverse	FALSE	0.6965	0.2121%	[u'aspC']
ADK1	FALSE	0.5882	0.1791%	[u'adk']
FADRx	FALSE	0.4866	0.1482%	[u'fre']
GLNS	TRUE	0.4208	0.1282%	[u'glnA', u'puuA']
PGCD	FALSE	0.3839	0.1169%	[u'serA']
PSP_L	FALSE	0.3839	0.1169%	[u'serB']
PSERT	FALSE	0.3839	0.1169%	[u'serC']
ICDHyr	TRUE	0.2687	0.0818%	[u'icd']
ACONTb	TRUE	0.2676	0.0815%	[u'acnA', u'acnB']
ACONTa	TRUE	0.2653	0.0808%	[u'acnA', u'acnB']
CS	TRUE	0.2635	0.0803%	[u'gltA']
ASAD_reverse	FALSE	0.2559	0.0779%	[u'asd']
ASPK	FALSE	0.2558	0.0779%	[u'lysC', u'thrA', u'metL']
GHMT2r	FALSE	0.2532	0.0771%	[u'glyA']
PItex	TRUE	0.2319	0.0706%	[u'ompN', u'ompC', u'phoE', u'ompF']
PIt2rpp	TRUE	0.2294	0.0699%	[u'pitA', u'pitB']
NDPK1	TRUE	0.2138	0.0651%	[u'ndk', u'adk']
KARA1_reverse	FALSE	0.2090	0.0636%	[u'ilvC']
MTHFD	FALSE	0.2086	0.0635%	[u'folD']
MTHFC	FALSE	0.2081	0.0634%	[u'folD']
ACLS	FALSE	0.2058	0.0627%	[u'ilvB', u'ilvH', u'ilvN', u'ilvI']
DHAD1	FALSE	0.2058	0.0627%	[u'ilvD']
PRPPS	FALSE	0.1996	0.0608%	[u'prs']
RPE_reverse	FALSE	0.1863	0.0567%	[u'sgcE', u'rpe']
RPI_reverse	FALSE	0.1810	0.0551%	[u'rpiA', u'rpiB']
MDH	TRUE	0.1703	0.0519%	[u'mdh']
FUM	TRUE	0.1702	0.0518%	[u'fumA', u'fumB', u'fumC']

HSDy_reverse	FALSE	0.1679	0.0511%	[u'metL', u'thrA']
CBMKr	FALSE	0.1518	0.0462%	[u'yqeA', u'yahI', u'ybcF']
TKT2_reverse	FALSE	0.1442	0.0439%	[u'tktA', u'tktB']
ALATA_L_reverse	FALSE	0.1398	0.0426%	[u'yfdZ', u'yfbQ']
TALA_reverse	FALSE	0.1393	0.0424%	[u'talA', u'talB']
IMPC_reverse	FALSE	0.1300	0.0396%	[u'purH']
AICART	FALSE	0.1294	0.0394%	[u'purH']
THRS	TRUE	0.1281	0.0390%	[u'thrC']
HSK	TRUE	0.1281	0.0390%	[u'thrB']
SUCOAS	TRUE	0.1191	0.0363%	[u'sucD', u'sucC']
HCO3E	FALSE	0.1170	0.0356%	[u'can', u'cynT']
NDPK2	TRUE	0.1105	0.0336%	[u'ndk', u'adk']
AIRC3_reverse	FALSE	0.1102	0.0336%	[u'purE']
IPPMIb_reverse	FALSE	0.1098	0.0334%	[u'leuC', u'leuD']
PRAGSr	FALSE	0.1091	0.0332%	[u'purD']
ADSL2r	FALSE	0.1080	0.0329%	[u'purB']
IPPMIa_reverse	FALSE	0.1080	0.0329%	[u'leuC', u'leuD']
LEUTAi	FALSE	0.1060	0.0323%	[u'ilvE', u'tyrB']
IPPS	FALSE	0.1060	0.0323%	[u'leuA']
IPMD	FALSE	0.1060	0.0323%	[u'leuB']
GLUPRT	FALSE	0.1054	0.0321%	[u'purF']
PRFGS	FALSE	0.1054	0.0321%	[u'purL']
PRAIS	TRUE	0.1054	0.0321%	[u'purM']
AIRC2	FALSE	0.1053	0.0321%	[u'purK']
PRASCSi	FALSE	0.1053	0.0321%	[u'purC']
VALTA_reverse	FALSE	0.1016	0.0309%	[u'ilvE']
