# Supplementary material for: Determining the Control Circuitry of Redox Metabolism at the Genome-Scale
Source: PLoS Genet. 2014 Apr 3;10(4):e1004264. doi: 10.1371/journal.pgen.1004264 (PMC3974632; doi:10.1371/journal.pgen.1004264)
Supplement: Table S8 — Mean flux values above .1 mmol/GDWH across all sampling points under nitrate conditons. This table shows all reactions, whether or not they are directly regulated by ArcA or Fnr, their mean flux values, the percent of the total flux that this flux values corresponds too, and the list of genes associated with the reaction. For each reaction the regulation column is TRUE if at least one gene is directly regulated by ArcA or Fnr. The total percent of flux regulated can then be calculated by summing across all flux values which are regulated and dividing by the total. (PDF) [file pgen.1004264.s016.pdf]

Supplementary Table 8. Mean flux values above .1mmol/GDWH across all sampling points under nitrate conditons. This table shows all reactions, whether or not they are direclty regulated by ArcA or Fnr, their mean flux values, the percent of the total flux that this flux values corresponds too, and the list of genes associated with the reaction. For each reaction the regulation column is TRUE if at least one gene is directly regulated by ArcA or Fnr. The total percent of flux regulated can then be calculated by summing across all flux values which are regulated and dividing by the total.

Reaction ID	Regulation	Flux	Percent total flux	Genes
ATPS4rpp	FALSE	39.2837	6.5468%	[u'atpG', u'atpB', u'atpI', u'atpH', u'atpF', u'atpE', u'atpC', u'atpD', u'atpA']
H2Otex_reverse	TRUE	37.7842	6.2969%	[u'ompN', u'ompC', u'ompL', u'ompA', u'ompG', u'phoE', u'ompF', None]
CO2tex_reverse	TRUE	26.3699	4.3947%	[u'ompN', u'ompF', u'phoE', u'ompC']
NADH16pp	TRUE	24.2813	4.0466%	[u'nuoL', u'nuoG', u'nuoH', u'nuoC', u'nuoI', u'nuoB', u'nuoJ', u'nuoN', u'nuoM', u'nuoA', u'nuoE', u'nuoF', u'nuoK']
NO3tex	TRUE	20.0058	3.3341%	[u'ompN', u'ompC', u'ompF', u'phoE']
NO3t7pp	TRUE	20.0000	3.3331%	[u'narU', u'narK']
H2Otpp_reverse	FALSE	18.2260	3.0375%	[u'aqpZ', None]
NADH17pp	TRUE	17.5415	2.9234%	[u'nuoL', u'nuoH', u'nuoJ', u'nuoC', u'nuoA', u'nuoB', u'nuoG', u'nuoN', u'nuoM', u'nuoI', u'nuoE', u'nuoF', u'nuoK']
GAPD	TRUE	17.2939	2.8821%	[u'gapA']
PGK_reverse	FALSE	17.2916	2.8817%	[u'pgk']
ENO	TRUE	15.9449	2.6573%	[u'eno']
PGM_reverse	FALSE	15.9404	2.6566%	[u'gpmM', u'ytjC', u'gpmA']
Htex	TRUE	12.2853	2.0474%	[u'ompN', u'ompF', u'ompC', u'phoE']
NO3R1pp	TRUE	12.2762	2.0459%	[u'narH', u'narJ', u'narI', u'narW', u'narG', u'narY', u'narV', u'narZ']
PDH	TRUE	10.5333	1.7554%	[u'aceE', u'aceF', u'lpd']
NO2tex_reverse	TRUE	10.2110	1.7017%	[u'ompN', u'ompC', u'phoE', u'ompF']
GLCptspp	TRUE	10.0000	1.6666%	[u'ptsI', u'manZ', u'manY', u'ptsG', u'malX', u'manX', u'crr', u'ptsH']
GLCtexi	FALSE	9.9933	1.6654%	[u'lamB']
NH4tpp	FALSE	8.6518	1.4419%	[u'amtB', None]
PGI	FALSE	8.5779	1.4296%	[u'pgi']
TPI	FALSE	8.5738	1.4289%	[u'tpiA']
ICDHyr	TRUE	8.1690	1.3614%	[u'icd']
ACONTb	TRUE	8.1674	1.3611%	[u'acnA', u'acnB']
ACONTa	TRUE	8.1657	1.3609%	[u'acnA', u'acnB']
CS	TRUE	8.1616	1.3602%	[u'gltA']
FUM	TRUE	8.1056	1.3508%	[u'fumA', u'fumB', u'fumC']
MDH	TRUE	8.1051	1.3508%	[u'mdh']
NO3R2pp	TRUE	7.7238	1.2872%	[u'narH', u'narJ', u'narI', u'narW', u'narZ', u'narY', u'narV', u'narG']
SUCDi	TRUE	7.3756	1.2292%	[u'sdhD', u'sdhA', u'sdhC', u'sdhB']
AKGDH	TRUE	7.2667	1.2110%	[u'sucB', u'lpd', u'sucA']
GLUDy_reverse	FALSE	6.8745	1.1457%	[u'gdhA']
SUCOAS_reverse	TRUE	6.8473	1.1411%	[u'sucD', u'sucC']
NTRIR3pp	TRUE	6.4872	1.0811%	[u'nrfC', u'nrfD', u'nrfA', u'nrfB']
FBA	FALSE	5.6263	0.9377%	[u'fbaB', u'fbaA', u'ydjI']
PFK	FALSE	5.6219	0.9369%	[u'pfkA', u'pfkB']
NTRIR4pp	TRUE	3.3053	0.5509%	[u'nrfB', u'nrfD', u'nrfA', u'nrfC']
PPKr_reverse	FALSE	3.0481	0.5080%	[u'ppk']
F6PA	TRUE	2.6748	0.4458%	[u'fsaB', u'fsaA']
DHAPT	FALSE	2.6694	0.4449%	[u'dhaL', u'dhaK', u'dhaM', u'ptsI', u'ptsH']
PPC	FALSE	2.4057	0.4009%	[u'ppc']
ASPTA_reverse	FALSE	2.3540	0.3923%	[u'aspC']
ADK1	FALSE	2.0778	0.3463%	[u'adk']
FADRx	FALSE	1.6523	0.2754%	[u'fre']
GLNS	FALSE	1.4290	0.2381%	[u'glnA', u'puuA']
PGCD	FALSE	1.3498	0.2250%	[u'serA']
PSP_L	FALSE	1.3498	0.2250%	[u'serB']
PSERT	FALSE	1.3498	0.2250%	[u'serC']

G6PDH2r	FALSE	1.2723	0.2120%	[u'zwf']
GND	FALSE	1.2689	0.2115%	[u'gnd']
PGL	FALSE	1.2689	0.2115%	[u'pgl']
NH4tex_reverse	TRUE	1.1559	0.1926%	[u'ompN', u'ompF', u'phoE', u'ompC']
RPI_reverse	FALSE	1.0340	0.1723%	[u'rpiA', u'rpiB']
GHMT2r	FALSE	0.8977	0.1496%	[u'glyA']
ASAD_reverse	FALSE	0.8663	0.1444%	[u'asd']
ASPK	TRUE	0.8647	0.1441%	[u'lysC', u'thrA', u'metL']
MTHFD	FALSE	0.7940	0.1323%	[u'folD']
MTHFC	FALSE	0.7929	0.1321%	[u'folD']
PItex	TRUE	0.7752	0.1292%	[u'ompN', u'ompC', u'phoE', u'ompF']
PIt2rpp	TRUE	0.7743	0.1290%	[u'pitA', u'pitB']
PRPPS	FALSE	0.7148	0.1191%	[u'prs']
KARA1_reverse	FALSE	0.7071	0.1178%	[u'ilvC']
ACLS	FALSE	0.6987	0.1164%	[u'ilvB', u'ilvH', u'ilvN', u'ilvI']
DHAD1	FALSE	0.6987	0.1164%	[u'ilvD']
NDPK1	TRUE	0.6921	0.1153%	[u'ndk', u'adk']
HSDy_reverse	TRUE	0.5677	0.0946%	[u'metL', u'thrA']
CBMKr	FALSE	0.5041	0.0840%	[u'yqeA', u'yahI', u'ybcF']
ALATA_L_reverse	FALSE	0.4610	0.0768%	[u'yfdZ', u'yfbQ']
ACKr	TRUE	0.4449	0.0741%	[u'purT', u'ackA', u'tdcD']
PTAr_reverse	TRUE	0.4414	0.0736%	[u'pta', u'eutD']
AICART	FALSE	0.4411	0.0735%	[u'purH']
IMPC_reverse	FALSE	0.4370	0.0728%	[u'purH']
THRS	TRUE	0.4349	0.0725%	[u'thrC']
HSK	TRUE	0.4349	0.0725%	[u'thrB']
FBA3	FALSE	0.4178	0.0696%	[u'fbaA']
PFK_3	FALSE	0.4138	0.0690%	[u'pfkA']
HCO3E	FALSE	0.3890	0.0648%	[u'can', u'cynT']
IPPMIb_reverse	FALSE	0.3664	0.0611%	[u'leuC', u'leuD']
PRAGSr	FALSE	0.3652	0.0609%	[u'purD']
IPPMIa_reverse	FALSE	0.3632	0.0605%	[u'leuC', u'leuD']
AIRC3_reverse	FALSE	0.3610	0.0602%	[u'purE']
ADSL2r	FALSE	0.3610	0.0602%	[u'purB']
GARFT	TRUE	0.3602	0.0600%	[u'purN']
LEUTAi	FALSE	0.3600	0.0600%	[u'ilvE', u'tyrB']
IPPS	FALSE	0.3600	0.0600%	[u'leuA']
IPMD	FALSE	0.3600	0.0600%	[u'leuB']
NDPK2	TRUE	0.3586	0.0598%	[u'ndk', u'adk']
GLUPRT	FALSE	0.3578	0.0596%	[u'purF']
PRFGS	FALSE	0.3578	0.0596%	[u'purL']
PRAIS	FALSE	0.3578	0.0596%	[u'purM']
AIRC2	FALSE	0.3576	0.0596%	[u'purK']
PRASCSi	FALSE	0.3576	0.0596%	[u'purC']
VALTA_reverse	FALSE	0.3402	0.0567%	[u'ilvE']
SHK3Dr	FALSE	0.3100	0.0517%	[u'aroE', u'ydiB']
PSCVT	FALSE	0.3096	0.0516%	[u'aroA']
DDPA	FALSE	0.3059	0.0510%	[u'aroH', u'aroG', u'aroF']
DHQTi	FALSE	0.3059	0.0510%	[u'aroD']
CHORS	FALSE	0.3059	0.0510%	[u'aroC']
DHQS	FALSE	0.3059	0.0510%	[u'aroB']
SHKK	FALSE	0.3059	0.0510%	[u'aroK', u'aroL']
SDPTA_reverse	FALSE	0.3026	0.0504%	[u'argD']
DAPE	FALSE	0.3016	0.0503%	[u'dapF']
UMPK	FALSE	0.3009	0.0501%	[u'cmk', u'pyrH']
DHDPS	FALSE	0.2969	0.0495%	[u'dapA']
SDPDS	FALSE	0.2969	0.0495%	[u'dapE']
THDPS	FALSE	0.2969	0.0495%	[u'dapD']
DHDPRy	FALSE	0.2969	0.0495%	[u'dapB']
TKT1	FALSE	0.2801	0.0467%	[u'tktA', u'tktB']
DAPDC	FALSE	0.2742	0.0457%	[u'lysA']
AGPR_reverse	FALSE	0.2733	0.0455%	[u'argC']
ACOTA_reverse	TRUE	0.2721	0.0454%	[u'astC', u'argD']

DHORTS_reverse	FALSE	0.2707	0.0451%	[u'pyrC']
ACODA	FALSE	0.2693	0.0449%	[u'argE']
ACGS	FALSE	0.2693	0.0449%	[u'argA']
ACGK	FALSE	0.2693	0.0449%	[u'argB']
ORPT_reverse	FALSE	0.2670	0.0445%	[u'pyrE']
ECOAH3	TRUE	0.2638	0.0440%	[u'fadB', u'fadJ']
OMPDC	FALSE	0.2634	0.0439%	[u'pyrF']
ASPCT	FALSE	0.2634	0.0439%	[u'pyrB', u'pyrI']
HACD2	TRUE	0.2629	0.0438%	[u'fadB', u'fadJ']
ECOAH5	TRUE	0.2626	0.0438%	[u'fadB', u'fadJ']
HACD4	TRUE	0.2621	0.0437%	[u'fadB', u'fadJ']
ECOAH4	TRUE	0.2618	0.0436%	[u'fadB', u'fadJ']
ECOAH2	TRUE	0.2616	0.0436%	[u'fadB', u'fadJ']
HACD3	TRUE	0.2615	0.0436%	[u'fadB', u'fadJ']
ACOAD4f_reverse	TRUE	0.2613	0.0435%	[u'fadE']
HACD5	TRUE	0.2612	0.0435%	[u'fadJ', u'fadB']
ACACT1r	TRUE	0.2610	0.0435%	[u'atoB', u'fadA', u'fadI']
ACACT3r	TRUE	0.2610	0.0435%	[u'fadA', u'fadI']
ACOAD2f_reverse	TRUE	0.2609	0.0435%	[u'fadE']
ECOAH1	TRUE	0.2609	0.0435%	[u'fadB', u'fadJ']
ACOAD5f_reverse	TRUE	0.2609	0.0435%	[u'fadE']
HACD1	TRUE	0.2607	0.0435%	[u'fadB', u'fadJ']
ACACT4r	TRUE	0.2606	0.0434%	[u'fadA', u'fadI']
ACACT5r	TRUE	0.2605	0.0434%	[u'fadA', u'fadI']
ACOAD1f_reverse	TRUE	0.2604	0.0434%	[u'fadE']
ACOAD3f_reverse	TRUE	0.2603	0.0434%	[u'fadE']
ACACT2r	TRUE	0.2601	0.0434%	[u'fadA', u'fadI']
CHORM	FALSE	0.2584	0.0431%	[u'pheA', u'tyrA']
ADSL1r	FALSE	0.2421	0.0404%	[u'purB']
OCBT	FALSE	0.2404	0.0401%	[u'argF', u'argI']
ARGSL	FALSE	0.2396	0.0399%	[u'argH']
ADSS	TRUE	0.2388	0.0398%	[u'purA']
ARGSS	FALSE	0.2364	0.0394%	[u'argG']
KARA2	FALSE	0.2362	0.0394%	[u'ilvC']
ILETA_reverse	FALSE	0.2359	0.0393%	[u'ilvE']
ACHBS	FALSE	0.2322	0.0387%	[u'ilvI', u'ilvH', u'ilvN', u'ilvB']
DHAD2	FALSE	0.2322	0.0387%	[u'ilvD']
THRD_L	FALSE	0.2322	0.0387%	[u'ilvA', u'tdcB']
ECOAH6	TRUE	0.2289	0.0381%	[u'fadJ', u'fadB']
HACD6	TRUE	0.2278	0.0380%	[u'fadB', u'fadJ']
ACOAD6f_reverse	TRUE	0.2273	0.0379%	[u'fadE']
ACACT6r	TRUE	0.2273	0.0379%	[u'fadA', u'fadI']
HACD7	TRUE	0.2262	0.0377%	[u'fadB', u'fadJ']
RPE	FALSE	0.2237	0.0373%	[u'sgcE', u'rpe']
ECOAH7	TRUE	0.2217	0.0369%	[u'fadB', u'fadJ']
ACACT7r	TRUE	0.2201	0.0367%	[u'fadA', u'fadI']
SO4tex	TRUE	0.2125	0.0354%	[u'ompN', u'ompF', u'phoE', u'ompC']
SERAT	FALSE	0.2077	0.0346%	[u'cysE']
SO4t2pp	FALSE	0.2072	0.0345%	[u'ychM']
PYK	TRUE	0.2059	0.0343%	[u'pykA', u'pykF']
SADT2	FALSE	0.2038	0.0340%	[u'cysN', u'cysD']
SULRi	FALSE	0.2038	0.0340%	[u'cysJ', u'cysI']
ADSK	FALSE	0.2038	0.0340%	[u'cysC']
CYSS	FALSE	0.2038	0.0340%	[u'cysK', u'cysM']
BPNT	FALSE	0.2038	0.0340%	[u'cysQ']
GK1	FALSE	0.2009	0.0335%	[u'gmk']
GMPS2	FALSE	0.1946	0.0324%	[u'guaA']
IMPD	TRUE	0.1946	0.0324%	[u'guaB']
ASNS2	FALSE	0.1926	0.0321%	[u'asnA']
TALA_reverse	FALSE	0.1794	0.0299%	[u'talA', u'talB']
TRDR	FALSE	0.1788	0.0298%	[u'trxB', u'trxA', u'trxC']
PAPSR	FALSE	0.1788	0.0298%	[u'cysH', u'trxC', u'trxA']
P5CR	FALSE	0.1767	0.0294%	[u'proC']

G5SD	FALSE	0.1767	0.0294%	[u'proA']
GLU5K	FALSE	0.1767	0.0294%	[u'proB']
PGMT_reverse	FALSE	0.1642	0.0274%	[u'yqaB', u'pgm']
DHORD5	TRUE	0.1624	0.0271%	[u'pyrD']
Ktex	TRUE	0.1567	0.0261%	[u'ompN', u'ompC', u'phoE', u'ompF']
Kt2pp	FALSE	0.1527	0.0255%	[u'kch', u'trkG', u'trkA', u'kup', u'sapD', u'trkH']
PHETA1_reverse	FALSE	0.1520	0.0253%	[u'aspC', u'tyrB', u'ilvE']
PPNDH	FALSE	0.1481	0.0247%	[u'pheA']
NDPK3	TRUE	0.1474	0.0246%	[u'ndk', u'adk']
ACOAD7f_reverse	TRUE	0.1429	0.0238%	[u'fadE']
CYTK1	FALSE	0.1397	0.0233%	[u'cmk']
G3PD2_reverse	FALSE	0.1368	0.0228%	[u'gpsA']
GLCS1	FALSE	0.1324	0.0221%	[u'glgA']
GLGC	FALSE	0.1324	0.0221%	[u'glgC']
MTHFR2	FALSE	0.1298	0.0216%	[u'metF']
METS	FALSE	0.1297	0.0216%	[u'metE', u'metH']
CYSTL	FALSE	0.1286	0.0214%	[u'metC', u'malY']
SHSL1	FALSE	0.1286	0.0214%	[u'metB']
HSST	FALSE	0.1286	0.0214%	[u'metA']
CTPS2	FALSE	0.1278	0.0213%	[u'pyrG']
TYRTA_reverse	FALSE	0.1136	0.0189%	[u'aspC', u'tyrB']
PPND	FALSE	0.1104	0.0184%	[u'tyrA']
