# Supplementary material for: Determining the Control Circuitry of Redox Metabolism at the Genome-Scale
Source: PLoS Genet. 2014 Apr 3;10(4):e1004264. doi: 10.1371/journal.pgen.1004264 (PMC3974632; doi:10.1371/journal.pgen.1004264)
Supplement: Table S9 — Regulation of fluxes around key metabolites in fermentative conditions. This table shows all of the biomass precursor, electron donor, and electron carrier molecules along with the associated flux amounts in which they are produced or consumed and the amount of this flux which is activated or repressed by ArcA and Fnr. (PDF) [file pgen.1004264.s017.pdf]

Supplementary Table 9. Regulation of fluxes around key metabolites in fermentative conditions. This table shows all of the biomass precursor, electron donor, and electron carrier molecules along with the associated flux amounts in which they are produced or consumed and the amount of this flux which is activated or repressed by ArcA and Fnr.

Metabolite ID	Direction	Flux	Percent activated	Percent repressed
h_c	producing	29.88	0.7694	0.02965
h_c	consuming	28.11	0.5807	0.01952
h_e	producing	27.78	1	0
nadh_c	consuming	20.2	0.9447	0.02695
nadh_c	producing	20.18	0.9612	0.009489
g3p_c	producing	19.53	0.0001866	0
g3p_c	consuming	19.53	0.9895	0
13dpg_c	producing	19.33	0.9998	0
13dpg_c	consuming	19.33	0.0001886	0
3pg_c	producing	19.33	0.0001422	0
3pg_c	consuming	19.33	0.9799	0
pep_c	producing	18.95	0	0
pep_c	consuming	18.95	0.9479	0
pyr_c	producing	18.04	0.9955	0
pyr_c	consuming	17.84	0.9683	0
accoa_c	producing	17.34	0.9968	0.001034
for_c	producing	17.28	0.9997	0
for_c	consuming	17.28	0.9983	0
accoa_c	consuming	17.26	0.9374	0.0463
g6p_c	producing	10.01	0.999	0
g6p_c	consuming	10.01	0	0
f6p_c	producing	9.974	0.0002192	0
f6p_c	consuming	9.974	0.0002192	0
ac_c	producing	8.246	0.982	0
etoh_c	producing	8.087	1	0
nadph_c	producing	3.444	0.8423	0.07803
nadph_c	consuming	3.324	0	0.001561
akg_c	producing	2.05	0	0.1717
akg_c	consuming	2.05	0	0.004515
oaa_c	producing	0.963	0	0.1768
oaa_c	consuming	0.9625	0	0.2763
fad_c	consuming	0.5031	0	0.03281
fadh2_c	producing	0.5031	0	0.03281
fad_c	producing	0.5029	0	1
fadh2_c	consuming	0.5029	0	1
fum_c	producing	0.2548	0	0.01115
e4p_c	producing	0.2375	0.00925	0
e4p_c	consuming	0.2375	0.009478	0
r5p_c	producing	0.2374	0	0
r5p_c	consuming	0.2374	0	0
fum_c	consuming	0.1877	0.05769	0.9065
mal__L_c	consuming	0.1731	0	1
mal__L_c	producing	0.1726	0	1
succ_c	producing	0.1435	0.0755	0.01942
succ_c	consuming	0.1396	0	0.8531
succoa_c	producing	0.1281	0	0.9298
succoa_c	consuming	0.1281	0	0.02175
glyc3p_c	producing	0.04302	0	0
glyc3p_c	consuming	0.04293	0	0
ac_c	consuming	0.0154	0.7876	0.2124
gthrd_c	producing	0.009242	0	0
gthrd_c	consuming	0.009242	0	0
amet_c	producing	0.004715	0	0
amet_c	consuming	0.004265	0	0
lac__D_c	producing	0.003533	0	0
lac__D_c	consuming	0.003533	0	0
h_e	consuming	0.003098	1	0

etoh_c	consuming	0.002348	1	0
gthrd_e	producing	0.002033	1	0
gthrd_e	consuming	0.002033	1	0
gthrd_p	producing	0.002033	1	0
gthrd_p	consuming	0.002033	1	0
h2_p	producing	0.002021	1	0
h2_p	consuming	0.002021	1	0
lac__L_c	producing	0.001994	0	0
lac__L_c	consuming	0.001994	0	0
h2_c	producing			
h2_c	consuming			
mal__D_c	producing			
mal__D_c	consuming			
