# Supplementary material for: Determining the Control Circuitry of Redox Metabolism at the Genome-Scale
Source: PLoS Genet. 2014 Apr 3;10(4):e1004264. doi: 10.1371/journal.pgen.1004264 (PMC3974632; doi:10.1371/journal.pgen.1004264)
Supplement: Table S10 — Regulation of fluxes around key metabolites in nitrate respiratory conditions. This table shows all of the biomass precursor, electron donor, and electron carrier molecules along with the associated flux amounts in which they are produced or consumed and the amount of this flux which is activated or repressed by ArcA and Fnr. (PDF) [file pgen.1004264.s018.pdf]

Supplementary Table 10. Regulation of fluxes around key metabolites in nitrate respiratory conditions. This table shows all of the biomass precursor, electron donor, and electron carrier molecules along with the associated flux amounts in which they are produced or consumed and the amount of this flux which is activated or repressed by ArcA and Fnr.

Metabolite ID	Direction	Flux	Percent activated	Percent repressed
13dpg_c	producing	17.3	0	0.9996
13dpg_c	consuming	17.3	0	0.0004859
3pg_c	producing	17.3	0	0
3pg_c	consuming	17.3	0	0
ac_c	producing	0.5107	0.03734	0
ac_c	consuming	0.5056	0.8799	0.1201
accoa_c	producing	11.17	0.03983	0.9508
accoa_c	consuming	10.91	0.001756	0.9126
akg_c	producing	14.2	0	0.5944
akg_c	consuming	14.2	0	0.5124
amet_c	producing	0.01225	0	0
amet_c	consuming	0.01161	0	0.0158
e4p_c	producing	0.4981	0	0.00715
e4p_c	consuming	0.4981	0	0.007518
etoh_c	producing	0.004112	0	1
etoh_c	consuming	0.004112	0	1
f6p_c	producing	8.623	0.0006181	0
f6p_c	consuming	8.623	0.3102	0
fad_c	producing	1.678	0	0.9995
fad_c	consuming	1.678	0	0.01519
fadh2_c	producing	1.678	0	0.01519
fadh2_c	consuming	1.678	0	0.9996
for_c	producing	0.008313	0	0.1103
for_c	consuming	0.008319	0	0
fum_c	producing	8.224	0	0.8975
fum_c	consuming	8.203	0	0.9881
g3p_c	producing	17.4	0.1537	0.0004831
g3p_c	consuming	17.4	0.0003063	0.9939
g6p_c	producing	10.02	0.9983	0
g6p_c	consuming	10.02	0	0
glyc3p_c	producing	0.1421	0	0
glyc3p_c	consuming	0.142	0.7626	0
gthrd_c	producing	0.03054	0	0
gthrd_c	consuming	0.03036	0	0
gthrd_e	producing	0.003648	1	0
gthrd_e	consuming	0.003648	1	0
gthrd_p	producing	0.003648	1	0
gthrd_p	consuming	0.003648	1	0
h2_c	producing			
h2_c	consuming			
h2_p	producing	0.003497	1	0
h2_p	consuming	0.003497	1	0
h_c	producing	98.86	0.01623	0.3449
h_c	consuming	79.82	0.7782	0.03026
h_e	producing	0.003595	1	0
h_e	consuming	12.29	1	0
lac__D_c	producing	0.005908	0	0.4837
lac__D_c	consuming	0.005908	0	0.4837
lac__L_c	producing	0.00352	0	1
lac__L_c	consuming	0.00352	0	1
mal__D_c	producing			
mal__D_c	consuming			
mal__L_c	producing	8.109	0	1
mal__L_c	consuming	8.111	0	1
nadh_c	producing	45.47	0.004279	0.9523
nadh_c	consuming	45.54	0.9189	0.0402
nadph_c	producing	11.61	0	0.704

nadph_c	consuming	11.2	0	0.05136
oaa_c	producing	10.52	0	0.7704
oaa_c	consuming	10.52	0	0.7762
pep_c	producing	15.95	0	0.9998
pep_c	consuming	15.95	0.6399	0.0005827
pyr_c	producing	13.11	0.7786	0
pyr_c	consuming	12.41	0	0.8488
r5p_c	producing	1.059	0	0
r5p_c	consuming	1.059	0	0
succ_c	producing	7.369	0	0.9292
succ_c	consuming	7.391	0	0.9986
succoa_c	producing	7.273	0	0.9998
succoa_c	consuming	7.273	0	0.9415
