# Supplementary material for: Determining the Control Circuitry of Redox Metabolism at the Genome-Scale
Source: PLoS Genet. 2014 Apr 3;10(4):e1004264. doi: 10.1371/journal.pgen.1004264 (PMC3974632; doi:10.1371/journal.pgen.1004264)
Supplement: Table S11 — This table shows the relative flux levels and differential transcriptional regulation values for reactions that differed by at least .25 mmol/GDWh mean sampled flux units between fermentative and nitrate respiratory conditions. This table contains 91 gene-associated reactions of which 89 are regulated by ArcA or Fnr. The first column is the COBRA reaction ID followed by the mean flux value in fermentative (fermen.) and nitrate respiratory (nitrate) conditions. The columns for Fermentative regulation and Nitrate regulation are the max absolute value levels of regulation (Fig. 3) cause by ArcA or Fnr under that condition across all genes associated with the metabolic reaction. The flux difference and regulation difference is always the value of the nitrate condition minus the value of the fermentation condition. The plot in figure 5c is between the last two columns of this table. (PDF) [file pgen.1004264.s019.pdf]

Supplementary Table 11. This table shows the relative flux levels and differential transcriptional regulation values for reactions that differed by at least .25 mmol/GDWh mean sampled flux units between fermentative and nitrate respiratory conditions. This table contains 91 gene associated reactions of which 89 are regulated by ArcA or Fnr. The first column is the COBRA reaction ID followed by the mean flux value in fermentative (fermen.) and nitrate respiratory (nitrate) conditions. The columns for Fermen. regulation and Nitrate regulation are the max absolute value levels of regulation (Fig 3) cause by ArcA or Fnr under that condtion across all genes associated with the metabolic reaction. The flux difference and regulation difference is always the value of the nitrate condition minus the value of the fermentation condition. The plot in figure 5c is between the the last two columns of this table.

Reaction ID	Fermen. flux	Nitrate respiratory flux	Genes	Fermen. regulation	Fermen. indirect regualtion	Nitrate regulation	Nitrate indirect regulation	Flux difference (Fermen-nitrate)	Regulation difference (Fermen. - nitrate)
PGM_reverse	18.94	15.94	[u'gpmM', u'ytjC', u'gpmA']	1.108	-3.79	0	-0.7715	3.004	1.108
ACLS	0.2058	0.6987	[u'ilvH', u'ilvN', u'ilvB', u'ilvI']	0	0.7276	0	4.523	-0.4929	0
ADSL2r	0.108	0.361	[u'purB']	0	0	0	0.2172	-0.253	0
LEUTAi	0.106	0.36	[u'ilvE', u'tyrB']	0	0.8977	0	1.852	-0.254	0
PRPPS	0.1996	0.7148	[u'prs']	0	1.132	0	0.6465	-0.5152	0
GLUPRT	0.1054	0.3578	[u'purF']	0	0.7997	0	0.5744	-0.2525	0
ACONTa	0.2653	8.166	[u'acnA', u'acnB']	-3.916	-3.916	-2.651	-2.651	-7.9	-1.265
PTAr_reverse	0.008366	0.4414	[u'eutD', u'pta']	2.708	2.708	0.9757	0.9757	-0.433	1.732
ACONTb	0.2676	8.167	[u'acnA', u'acnB']	-3.916	-3.916	-2.651	-2.651	-7.9	-1.265
NO3R2pp	2.787E-13	7.724	[u'narY', u'narG', u'narV', u'narZ', u'narH', u'narJ', u'narI', u'narW']	4.02	4.02	10.61	10.61	-7.724	-6.592
PFK_3	0.08597	0.4138	[u'pfkA']	0	0.8878	0	0	-0.3278	0
THRS	0.1281	0.4349	[u'thrC']	0.5021	0.5021	0.3755	0.3755	-0.3068	0.1266
ADK1	0.5882	2.078	[u'adk']	0	0.7634	0	0	-1.49	0
CS	0.2635	8.162	[u'gltA']	-3.396	-3.396	-2.718	-2.718	-7.898	-0.6781
THD2pp	2.901	1.608E-13	[u'pntB', u'pntA']	0.72	1.283	0.822	0.822	2.901	-0.102
ACALD_reverse	8.087	0.003197	[u'mhpF', u'adhE']	0.5875	0.5875	-3.749	-3.749	8.084	4.336
GLNS	0.4208	1.429	[u'puuA', u'glnA']	-3.3	-3.3	0	0	-1.008	-3.3
RPI_reverse	0.181	1.034	[u'rpiB', u'rpiA']	0	-0.5984	0	-0.8368	-0.853	0
GLUDy_reverse	2.012	6.874	[u'gdhA']	0	-0.9882	0	0	-4.863	0
G6PDH2r	0.002477	1.272	[u'zwf']	0	-0.8385	0	0.1566	-1.27	0
HSK	0.1281	0.4349	[u'thrB']	0.3449	0.3449	-0.262	-0.262	-0.3068	0.6068
IPPMIa_reverse	0.108	0.3632	[u'leuD', u'leuC']	0	1.107	0	1.544	-0.2552	0
NH4tpp	2.549	8.652	[u'amtB', None]	0	-1.648	0	-5.893	-6.103	0
ASAD_reverse	0.2559	0.8663	[u'asd']	0	-0.4876	0	-0.4261	-0.6104	0
PGCD	0.3839	1.35	[u'serA']	0	0.6601	0	0	-0.9659	0
ENO	18.95	15.94	[u'eno']	0	0.1571	-0.7088	-0.7088	3.001	0.7088
IPPMIb_reverse	0.1098	0.3664	[u'leuD', u'leuC']	0	1.107	0	1.544	-0.2566	0
PDH	-7.209E-14	10.53	[u'aceE', u'aceF', u'lpd']	-2.346	-2.346	-1.264	-1.264	-10.53	-1.083
ASPTA_reverse	0.6965	2.354	[u'aspC']	0	0	0	-0.3074	-1.657	0

NADH17pp	3.625E-12	17.54	[u'nuoB', u'nuoN', u'nuoC', u'nuoM', u'nuoH', u'nuoA', u'nuoE', u'nuoG', u'nuoJ', u'nuoK', u'nuoF', u'nuoL',	3.419	3.419	2.833	2.833	-17.54	0.5856
FORtppi	17.25	1.336E-11	[u'focA', u'focB']	0.6645	0.6645	-0.6248	-0.6248	17.25	1.289
AKGDH	-1.529E-13	7.267	[u'sucB', u'sucA', u'lpd']	-4.779	-4.779	-3.497	-3.497	-7.267	-1.283
TPI	9.706	8.574	[u'tpiA']	0	0.3547	0	-0.124	1.132	0
GARFT	0.08194	0.3602	[u'purN']	0.7729	1.386	0.2716	0.4197	-0.2782	0.5014
GND	3.054E-13	1.269	[u'gnd']	0	0	0	0	-1.269	0
PRFGS	0.1054	0.3578	[u'purL']	0	1.177	0	0.3985	-0.2525	0
HCO3E	0.117	0.389	[u'can', u'cynT']	0	-1.428	0	-1.624	-0.272	0
ICDHyr	0.2687	8.169	[u'icd']	-2.117	-2.117	-1.345	-1.345	-7.9	-0.7716
FBA	1.795	5.626	[u'fbaA', u'fbaB', u'ydjI']	0	-1.548	0	-0.5799	-3.831	0
PFK	1.793	5.622	[u'pfkA', u'pfkB']	0	0.8878	0	-0.3968	-3.829	0
PFL	17.27	7.66E-12	[u'pflC', u'pflD', u'pflB', u'tdcE', u'pflA', u'yfiD']	3.578	3.578	4.726	4.726	17.27	-1.149
PGK_reverse	19.33	17.29	[u'pgk']	0	0.3855	0	-0.3484	2.038	0
DHAD1	0.2058	0.6987	[u'ilvD']	0	1.599	0	1.772	-0.4929	0
KARA1_reverse	0.209	0.7071	[u'ilvC']	0	0.2638	0	0	-0.4981	0
PGL	1.929E-11	1.269	[u'pgl']	0	-2.06	0	-0.424	-1.269	0
PGI	9.956	8.578	[u'pgi']	0	-0.3461	0	-0.767	1.378	0
FADRx	0.4866	1.652	[u'fre']	0	0	0	-0.3758	-1.166	0
ALATA_L_rever	0.1398	0.461	[u'yfbQ', u'yfdZ']	0	0	0	1.968	-0.3212	0
PRAIS	0.1054	0.3578	[u'purM']	0.3123	0.8902	0	0.3384	-0.2525	0.3123
NTRIR3pp	8.022E-14	6.487	[u'nrfB', u'nrfA', u'nrfC', u'nrfD']	5.259	5.259	6.174	6.174	-6.487	-0.9149
PSP_L	0.3839	1.35	[u'serB']	0	0.497	0	0.2683	-0.9659	0
AIRC3_reverse	0.1102	0.361	[u'purE']	0	0.9526	0	-0.3078	-0.2508	0
PPKr_reverse	0.8995	3.048	[u'ppk']	0	-0.3632	0	-0.6181	-2.149	0
AIRC2	0.1053	0.3576	[u'purK']	0	0.7131	0	0.2822	-0.2523	0
PRASCSi	0.1053	0.3576	[u'purC']	0	0.6648	0	0	-0.2523	0
HSDy_reverse	0.1679	0.5677	[u'metL', u'thrA']	0	0	-0.283	-0.283	-0.3998	0.283
NADH16pp	3.585E-12	24.28	[u'nuoB', u'nuoN', u'nuoK', u'nuoM', u'nuoH', u'nuoA', u'nuoE', u'nuoG', u'nuoJ', u'nuoC', u'nuoF', u'nuoL',	3.419	3.419	2.833	2.833	-24.28	0.5856
IPPS	0.106	0.36	[u'leuA']	0	0.6384	0	1.097	-0.254	0
PSERT	0.3839	1.35	[u'serC']	0	[illegible]	0	0.3502	-0.9659	0

H2Otpp	1.848	0.01737	[u'aqpZ', None]	0	0	0	-0.7617	1.83	0
AICART	0.1294	0.4411	[u'purH']	0	1.457	0	0.3499	-0.3118	0
TKT1	0.002543	0.2801	[u'tktB', u'tktA']	0	-4.138	0	-1.084	-0.2775	0
NTRIR4pp	5.517E-13	3.305	[u'nrfD', u'nrfA', u'nrfC', u'nrfB']	5.259	5.259	6.174	6.174	-3.305	-0.9149
PTAr	8.094	0.01554	[u'eutD', u'pta']	2.708	2.708	0.9757	0.9757	8.079	1.732
MTHFC	0.2081	0.7929	[u'folD']	0	0	0	-0.4093	-0.5849	0
MTHFD	0.2086	0.794	[u'folD']	0	0	0	-0.4093	-0.5854	0
SUCOAS_rever	0.002786	6.847	[u'sucD', u'sucC']	-5.539	-5.539	-2.969	-2.969	-6.844	-2.571
ATPS4rpp_reve	6.444	0.004692	[u'atpB', u'atpH', u'atpF', u'atpE', u'atpC', u'atpD', u'atpG', u'atpA', u'atpI']	0	1.449	0	0.4722	6.44	0
PIt2rpp	0.2294	0.7743	[u'pitA', u'pitB']	2.765	2.765	1.241	1.355	-0.5449	1.523
MDH	0.1703	8.105	[u'mdh']	-3.83	-3.83	-1.735	-1.735	-7.935	-2.094
ASPK	0.2558	0.8647	[u'lysC', u'metL', u'thrA']	0	-2.525	-0.283	-1.032	-0.6089	0.283
FBA3	0.0888	0.4178	[u'fbaA']	0	0.2582	0	0	-0.329	0
ACKr_reverse	8.098	0.01907	[u'tdcD', u'purT', u'ackA']	1.147	1.147	0.7709	0.8997	8.079	0.3761
CBMKr	0.1518	0.5041	[u'yahI', u'yqeA', u'ybcF']	0	0	0	1.047	-0.3523	0
ATPS4rpp	0.004155	39.28	[u'atpB', u'atpH', u'atpF', u'atpE', u'atpC', u'atpD', u'atpG', u'atpA', u'atpI']	0	1.449	0	0.4722	-39.28	0
FUM	0.1702	8.106	[u'fumA', u'fumB', u'fumC']	-7.374	-7.374	-3.164	-3.818	-7.935	-4.211
ALCD2x_revers	8.087	0.004112	[u'frmA', u'adhE', u'adhP']	0.5875	-7.416	-1.103	-1.103	8.083	1.691
H2Otpp_revers	0.006718	18.23	[u'aqpZ', None]	0	0	0	-0.7617	-18.22	0
NDPK1	0.2138	0.6921	[u'adk', u'ndk']	-3.066	-3.066	-2.573	-2.573	-0.4783	-0.4922
GHMT2r	0.2532	0.8977	[u'glyA']	0	0.7057	0	0.4058	-0.6446	0
IPMD	0.106	0.36	[u'leuB']	0	1.151	0	1.733	-0.254	0
GAPD	19.33	17.29	[u'gapA']	0.2259	0.2259	-0.1395	-0.1395	2.035	0.3655
NO3R1pp	3.521E-14	12.28	[u'narY', u'narG', u'narV', u'narZ', u'narH', u'narJ', u'narI', u'narW']	4.02	4.02	10.61	10.61	-12.28	-6.592

ACKr	0.01213	0.4449	[u'tdcD', u'purT', u'ackA']	1.147	1.147	0.7709	0.8997	-0.4328	0.3761
PPC	0.7866	2.406	[u'ppc']	0	0	0	0	-1.619	0
PRAGSr	0.1091	0.3652	[u'purD']	0	0.7674	0	0.873	-0.2561	0
SUCDi	1.581E-12	7.376	[u'sdhA', u'sdhD', u'sdhC', u'sdhB']	-10.01	-10.01	-6.876	-6.876	-7.376	-3.136
IMPC_reverse	0.13	0.437	[u'purH']	0	1.457	0	0.3499	-0.307	0
DHAPT	7.865	2.669	[u'dhaK', u'dhaM', u'ptsH', u'dhaL', u'ptsI']	2.049	2.049	0	0.8706	5.195	2.049
F6PA	7.867	2.675	[u'fsaA', u'fsaB']	0	0	0.35	0.35	5.192	-0.35
NO3t7pp	5.017E-13	20	[u'narK', u'narU']	0	0	8.447	8.447	-20	-8.447
