# Supplementary material for: Determining the Control Circuitry of Redox Metabolism at the Genome-Scale
Source: PLoS Genet. 2014 Apr 3;10(4):e1004264. doi: 10.1371/journal.pgen.1004264 (PMC3974632; doi:10.1371/journal.pgen.1004264)
Supplement: Table S12 — Regulation of altered reactions based on sampling of flux solutions between fermentative and nitrate respiratory conditions. After calculating the set of reactions which differ in their flux values between fermentative and nitrate respiratory conditions we sought to understand how many of these reactions were regulated by ArcA and Fnr. Altered reactions describes the total number of reactions which differ between the conditions by the flux cutoff (e.g. 91 reactions differ between the two conditions by at least .25 mmol/GDW-h). Of these 91 reactions, 40 are directly regulated by ArcA or Fnr and another 49 are indirectly regulated. (PDF) [file pgen.1004264.s020.pdf]

Supplementary Table 12. Regulation of altered reactions based on sampling of flux solutions between fermentative and nitrate respiratory conditions. After calculating the set of reactions which differ in their flux values between fermentative and nitrate respiratory conditions we sought to understand how many of these reactions were regulated by ArcA and Fnr. Altered reactions describes the total number of reactions which differ between the conditions by the flux cutoff (e.g. 91 reactions differ between the two conditions by at least .25 mmol/GDW-h). Of these 91 reactions, 40 are directly regulated by ArcA or Fnr and another 49 are indirectly regulated.

Flux cutoff (mmol/GDW-h)	Altered reactions	Directly regulated reactions	Indirectly regulated reactions	Total reactions regulated	Percent reactions regulated
0.25	91	40	49	89	0.978021978
0.1	180	75	98	173	0.961111111
0.05	213	83	121	204	0.957746479
0.01	303	105	176	281	0.927392739
0.005	332	109	197	306	0.921686747
0.001	801	213	426	639	0.797752809
