# Supplementary material for: Determining the Control Circuitry of Redox Metabolism at the Genome-Scale
Source: PLoS Genet. 2014 Apr 3;10(4):e1004264. doi: 10.1371/journal.pgen.1004264 (PMC3974632; doi:10.1371/journal.pgen.1004264)
Supplement: Table S13 — List of all transcription factors found in RegulonDB that map to metabolites in the iJO1366 metabolic model. (PDF) [file pgen.1004264.s021.pdf]

| Transcription factor gene name | Metabolite ID from iJO1366 | Metabolite name |
|---|---|---|
| appY | o2_c | |
| narL | no3_c | |
| dcuR | fum_c | |
| gadX | glu-L_c | |
| gadW | glu-L_c | |
| gadE | glu-L_c | |
| birA | btn_c biotin | |
| caiF | crnDcoa_c carnitine | |
| betI | g3pc_c choline | |
| cysB | acser_c O-Acetyl-L-serine | |
| dhaR | dha_c dihydroxyacetone | |
| fabR | actACP_c,aacoa_c acetyl-phosphate,acetyl-CoA | |
| fadR | actACP_c acetyl-phosphate | |
| fhlA | for_c formate | |
| glcC | glcn_c gluconate | |
| lldR | lac-D_c,lac-L_c D-lactate,L-lactate | |
| metJ | hcys-L_c L-Homocysteine | |
| metR | amet_c S-Adenosylmethionine | |
| mhpR | 3hpppn_c 3-(3-hydroxy-phenyl)propionate | |
| modE | moco_c | molybdenum |
| nac | nh4_c | low_nitrogen |
| nikR | ni2_c | nickel |
| crp | camp_c | |
| oxyR | o2s_c | superoxide anion |
| pdhR | pyr_c | pyruvate |
| putA | pro-L_c proline | |
| rbsR | rib-D_c | ribose |
| fur | fe2_c | Fe_2+ |
| iclR | pep_c,pyr_c,glx_c | phosphoenolpyruvate |
| kdgR | 2ddglcn_c | 2-Dehydro-3-deoxy-D- |
| marA | 4hbz_c | 4-hydroxybenzoate/2- |
| marR | 23dhb_c | 2,3-Dihydroxybenzoat |
| nhaR | na1_c | Sodium |
| zraR | zn2_c | Zinc |
| argP | arg-L_c,lys-L_c | arginine,lysine |
| argR | arg-L_c | arginine |
| asnC | asn-L_c | asparaginine |
| cueR | cu_c copper | |
| cusR | cu2_c copper2 | |
| cynR | cynt_c cyanate | |
| cytR | cytd_c cytidine | |
| dnaA | atp_c ATP | |

| | | |
|---|---|---|
| dsdC | ser-D_c D-serine | |
| galR | gal_c galactose | |
| galS | gal_c galactose | |
| gcvA | gly-L_c L-glycine | |
| hcaR | 3hpppn_c 3-phenylpropionate | |
| iscR | fe2_c Fe_2+ | |
| lysR | lys-L_c L-lysine | |
| prpR | micit_c methylisocitrate | |
| malT | malt_c maltose | |
| rpoS | stress | |
| trpR | trp__L_c | |
| tyrR | tyr__L_c | |
| rutR | thym_c | ura_c |
| alaS | ala__D_c | |
| allR | glx_c | |
| allS | alltn_c | |
| alsR | all__D_c | |
| araC | arab__L_c | |
| asnC | asn__L_c | |
| cdaR | glyc__R_c | |
| deoR | r5p_c | |
| dpiA | cit_c | |
| xylR | xyl__D_c | |
| xapR | xtsn_c | |
| uxuR | fruur_c | |
| ulaR | ascb6p_c | |
| uhpA | g6p_e | |
| treR | tre6p_c | |
| soxR | o2s_c | |
| rhaR | rmn_c | |
| rhaS | rmn_c | |
| puuR | ptrc_c | |
| purR | hxan_c | |
| paaX | phaccoa_c | |
| nsrR | no_c | |
| nemR | mthgxl_c | |
| narP | no3_c | |
| nanR | acnam_c | |
| nagC | acgam6p_c | |
| murR | acmum6p_c | |
| mtlR | mnl_e | |
| melR | melib_c | |
| idnR | 5dglcn_c | |

| | |
|---|---|
| gntR | glcn_c |
| glpR | glyc3p_c |
| galR | gal_c |
| galS | gal_c |

,pyruvate,glyoxylate
-gluconate
-Hydroxybenzoic_acid
te
