# Supplementary material for: Determining the Control Circuitry of Redox Metabolism at the Genome-Scale
Source: PLoS Genet. 2014 Apr 3;10(4):e1004264. doi: 10.1371/journal.pgen.1004264 (PMC3974632; doi:10.1371/journal.pgen.1004264)
Supplement: Text S1 — Transport coupled redox balancing as shown in Fig. S4 is explained in greater detail. Briefly, only 5 genes are found that encode for reactions which produce NAD(P)H and are not regulated by ArcA or Fnr. Interestingly, 4/5 of these genes are amino acid biosynthetic enzymes. Two of these enzymes in particular, serA and tyrA, are feedback inhibited by serine and tyrosine respectively. Thus, as shown in Figure S4 we are able to corroborate dramatic regulation of the sstT serine transporter and the aroP tyrosine transporter with feedback inhibition of these critical biosynthetic enzymes. Under this regulatory scheme, serine and tyrosine would be produced at the expense of critical redox potential but immediately shut down if any serine or tyrosine can be scavenged exogenously. (DOC) [file pgen.1004264.s022.doc]

Text S1.

Transport coupled redox balancing

The NADH/NAD+ redox pair is critical because a high concentration of NAD+ is absolutely necessary to run glyceraldehyde 3-phosphate dehydrogenase and allow glycolysis to proceed. If the ratio of the NADH/NAD+ becomes too high then the cell will not be able to run glycolysis and perish. Thus a careful system of redox balancing has evolved in which respiratory metabolism is focused on the transfer of reducing equivalents through NADH and under conditions in which respiration becomes impossible the reducing equivalents are dumped onto glycolytic intermediates in the process of fermentation. While the general principle of redox balancing has been widely disseminated and utilized[1] and even hypothesized to be mediated by ArcA or Fnr it was not previously possible to mechanistically determine how this phenomena proceeds at the systems level. We took a genome-scale model of E. coli metabolism and sampled it using Monte Carlo methods to determine flux range distributions for all reactions under both fully anaerobic and anaerobic with the addition of nitrate. We then looked at every reaction which utilized NADH/NAD+ and found that 14/19, and 30/35 genes encoding reactions under anaerobic and nitrate conditions were directly regulated by ArcA or Fnr. Drilling down into the 5 unregulated genes (same in both conditions) we found that one encoded *fre*, a constitutively expressed NAD generation enzyme, and the other four, *serA, tyrA, metF*, and *hisD* all encoded amino acid metabolic enzymes. We then took into consideration a puzzling finding of newly discovered and highly significant (fold change regulation of amino acid transporters for serine, tyrosine, methionine and histidine. We noticed that for *serA* and *tyrA* in particular, the NADH generating reactions were the subject of end product inhibition by serine and tyrosine. Thus we can hypothesize that activation of the uptake transporters for these amino acids will cause feedback inhibition of the enzymes and thus maintain the expression of critical metabolic enzymes while simultaneously modulating their redox related contributions (Fig. S4).

1. Clark D (1989) The fermentation pathways of Escherichia coli. FEMS microbiology reviews 63: 223–234.
